# GWAS of allometric body-shape indices in UK Biobank identifies loci suggesting associations with morphogenesis, organogenesis, adrenal cell renewal and cancer

**DOI:** 10.1038/s41598-021-89176-6

**Published:** 2021-05-21

**Authors:** Sofia Christakoudi, Evangelos Evangelou, Elio Riboli, Konstantinos K. Tsilidis

**Affiliations:** 1 Department of Epidemiology and Biostatistics, School of Public Health, Imperial College London, St Mary’s Campus, Norfolk Place, London, W2 1PG UK; 2 MRC Centre for Transplantation, King’s College London, Great Maze Pond, London, SE1 9RT UK; 3 Department of Hygiene and Epidemiology, University of Ioannina School of Medicine, Ioannina, Greece

**Keywords:** Genome-wide association studies, Obesity

## Abstract

Genetic studies have examined body-shape measures adjusted for body mass index (BMI), while allometric indices are additionally adjusted for height. We performed the first genome-wide association study of A Body Shape Index (ABSI), Hip Index (HI) and the new Waist-to-Hip Index and compared these with traditional indices, using data from the UK Biobank Resource for 219,872 women and 186,825 men with white British ancestry and Bayesian linear mixed-models (BOLT-LMM). One to two thirds of the loci identified for allometric body-shape indices were novel. Most prominent was *rs72959041* variant in *RSPO3* gene, expressed in visceral adipose tissue and regulating adrenal cell renewal. Highly ranked were genes related to morphogenesis and organogenesis, previously additionally linked to cancer development and progression. Genetic associations were fewer in men compared to women. Prominent region-specific associations showed variants in loci *VEGFA* and *HMGA1* for ABSI and *KLF14* for HI in women, and *C5orf67* and *HOXC4/5* for ABSI and *RSPO3, VEGFA* and *SLC30A10* for HI in men. Although more variants were associated with waist and hip circumference adjusted for BMI compared to ABSI and HI, associations with height had previously been reported for many of the additional variants, illustrating the importance of adjusting correctly for height.

## Introduction

The cardiometabolic complications of obesity are influenced by body shape, showing a positive association with abdominal size and an inverse association with gluteofemoral size^1^. The waist-to-hip ratio (WHR) and body mass index (BMI) are used correspondingly as an index of body shape and an index of general obesity^2^. The WHR, however, is moderately correlated with BMI and cannot differentiate abdominal from gluteofemoral size. Although waist (WC) and hip circumference (HC) are measures of specific body regions, they are both strongly correlated with BMI. To account for the correlation with BMI, genome-wide association studies (GWAS) have used traditionally residuals from linear models regressing each of WHR, WC, or HC on BMI, thus creating body-shape indices independent from BMI^3,4^. Nevertheless, the adjustment of WC or HC for BMI introduces a positive correlation with height, which is stronger than the association of WC or HC with height^4^.

An alternative approach to creating body-shape indices independent from body size and general obesity has been implemented in the development of A Body Shape Index (ABSI) and Hip Index (HI)^5,6^. Similarly to BMI, ABSI and HI are based on the principle of allometry, which accounts for the expansion of the dimensions of individual body parts relative to the total body size with log-linear rather than linear models^7^. Similar to the scaling of log-transformed weight to log-transformed height used for the development of BMI, log-transformed WC and HC have each been scaled to log-transformed weight and log-transformed height to develop ABSI and HI^5,6^. This scaling accounts for the expansion of body circumferences proportional to body size, as reflected in height, and additionally accounts for the proportional expansion of body circumferences with general adiposity, as reflected in body weight. Consequently, the allometric body-shape indices are independent by design from height, as well as from BMI. Strong associations of ABSI with mortality and cardio-metabolic risk factors have been reported^5,8^ and we have demonstrated that ABSI achieves better mortality risk stratification than alternative body-shape indices, which are correlated with BMI^9^.

There are, however, no studies to date examining the genetic associations of ABSI and HI, no allometric counterpart of WHR and no insight into the influence of the correlation of traditional body-shape indices with height. The aims of our study, therefore, were to perform the first GWAS of allometric body-shape indices and to compare allometric and traditional body-shape indices with respect to their genetic associations. Our GWAS provides novel information for unbiased genetic associations of body-shape indices.

## Results

We used data from the UK Biobank for 219,872 women and 186,825 men with white British ancestry (Supplementary Table S1). For allometric body-shape indices, we used ABSI, HI and a waist-to-hip index (WHI) calibrated for UK Biobank participants (ABSI_UKB_, HI_UKB_ and WHI_UKB_, see details in Methods). For traditional body-shape indices, we used WC, HC and WHR adjusted for BMI in linear models (WC_adj_BMI, HC_adj_BMI and WHR_adj_BMI). We examined women and men separately, as pronounced sexual dimorphisms have been reported for the genetic associations of traditional body-shape indices^10^.

### Genetic variants associated with allometric body-shape indices

We determined independent significant single nucleotide polymorphisms (SNPs) and consolidated them in independent genomic risk loci, represented by a locus lead SNP, with Functional Mapping and Annotation (FUMA) (see details in Methods).

A larger number of independent genomic risk loci were associated with WHI_UKB_ (282 in women, 97 in men) compared to ABSI_UKB_ (200 in women, 65 in men) and HI_UKB_ (171 in women, 75 in men) (Table 1). The highest-ranked lead SNPs associated with WHI_UKB_ (*rs72959041* in women and *rs577721086* in men) were both in the *RSPO3* locus and in very strong linkage disequilibrium (r^2^ > 0.99). In addition, *rs72959041* was the second highest-ranked lead SNP for ABSI_UKB_ in women and was the highest-ranked lead SNP for HI_UKB_ in both sexes (Supplementary Table S2). This variant stood out with some of the largest effect sizes, with a positive sign of the regression coefficients for ABSI_UKB_ and WHI_UKB_ and a negative sign for HI_UKB_, reflecting the phenotype of the minor allele. Most of the independent variants associated with WHI_UKB_ similarly showed regression coefficients with opposite signs for ABSI_UKB_ and HI_UKB_ (Supplementary Fig. S1). Nevertheless, some variants were associated preferentially with ABSI_UKB_ or HI_UKB_. In women, the most outstanding examples of loci associated exclusively with ABSI_UKB_ were *VEGFA* and *HMGA1*, while the most prominent locus associated exclusively with HI_UKB_ was *KLF14*. In men, prominent loci with variants associated exclusively with ABSI_UKB_ were *AC022431.2 (C5orf67), RP11-115J16.1, RP5-859D4.3 (CASC20)* and the region including loci *HOXC4*, *RP11-834C11.14* and *HOXC5*, while the most prominent loci associated exclusively with HI_UKB_ were *RSPO3, VEGFA* and *RP11-95P13.2* (*SLC30A10*) (Supplementary Fig. S2).

**Table 1:**
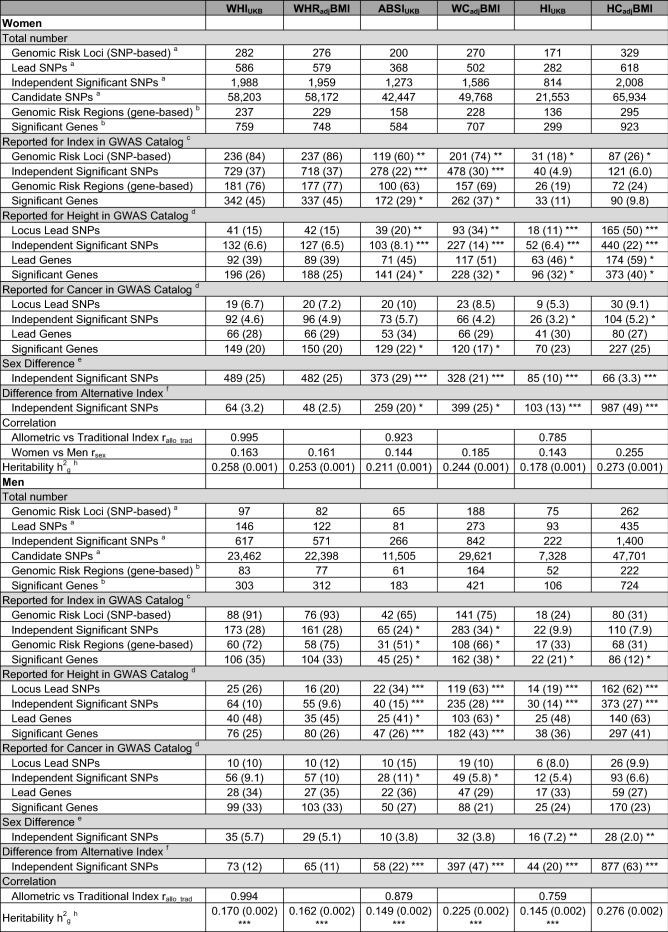
Counts and overlaps of independent genetic variants and loci associated with allometric and traditional body-shape indices in women and men.

^a^Based on FUMA; ^b^Based on MAGMA; ^c^Number (percentage from total per index) reported in the NHGRI-EBI GWAS Catalog^11^ (https://www.ebi.ac.uk/gwas/home, accessed on 07/04/2021) in association with the corresponding traditional body-shape index (with or without adjustment for body mass index, BMI), i.e. the waist-to-hip ratio for WHI_UKB_ and WHR_adj_BMI (catalogue sets EFO_0004343, EFO_0007788, EFO_0004302), waist circumference for ABSI_UKB_ and WC_adj_BMI (EFO_0004342, EFO_0007789, EFO_0004302), hip circumference for HI_UKB_ and HC_adj_BMI (EFO_0005093, EFO_0008039, EFO_0004302). To ensure novelty, a match was counted for an independent significant SNP if any of the candidate SNPs in the LD block was reported and for a genomic risk locus (or region) if any of the corresponding independent significant SNPs (or genes) was reported; ^d^Number (percentage from total per index), counting indirect matches only for SNPs in strong LD, i.e. candidate SNPs within the corresponding LD block for independent significant SNPs and for a genomic risk locus (or region) only a match of the independent significant SNP (or gene) promoted to a locus lead SNP (or lead gene) (EFO_0004339, EFO_0004302 for height; EFO_0000311 for cancer); ^e^Number (percentage from total per index) showing sex difference (p_difference_ < 5*10^–6^ for any candidate SNP within the corresponding LD block); ^f^As for ^e^but reflecting difference from the corresponding alternative body-shape index, i.e. WHR_adj_BMI for WHI_UKB_, WC_adj_BMI for ABSI_UKB_, HC_adj_BMI for HI_UKB_ and vice versa; ABSI_UKB_-a body shape index calibrated for UK Biobank participants; HC_adj_BMI-hip circumference adjusted for BMI; HI_UKB-_hip index calibrated for UK Biobank participants; WC_adj_BMI-waist circumference adjusted for BMI; WHI_UKB_-waist-to-hip index calibrated for UK Biobank participants; WHR_adj_BMI-waist-to-hip ratio adjusted BMI; h^2^_g_-estimated (pseudo-) heritability, based on the genetic relationship matrix in BOLT-LMM (approximate standard error, 316/number of individuals), comparison between men and women; r_sex_-Spearman’s rank correlation coefficient between regression coefficients in women and men across all examined genetic variants (used in the test for difference of effect size or heritability); r_allo-trad_-correlation coefficient as for r_sex_ but between the corresponding allometric and traditional index; Fisher’s exact test was used to compare percentages between allometric and traditional body-shape indices: *P < 0.05; **P < 0.001; ***P < 0.0001.

**Figure 1:**
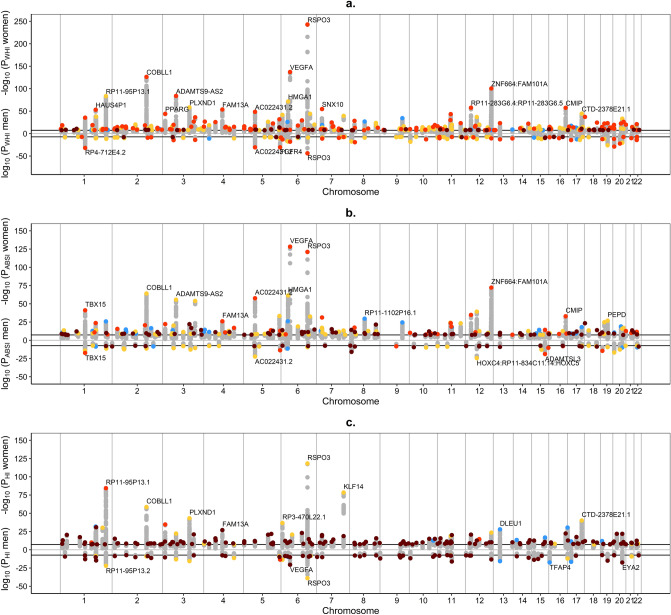
Miami plots of candidate SNPs identified for allometric body-shape indices in women and men. (**a**) GWAS of waist-to-hip index calibrated for UK Biobank participants (WHI_UKB_); (**b**) GWAS of a body shape index calibrated for UK Biobank participants (ABSI_UKB_); (**c**) GWAS of hip index calibrated for UK Biobank participants (HI_UKB_); P-*P*-values were derived from BOLT-LMM infinitesimal models; SNP-single nucleotide polymorphism; horizontal lines correspond to the genome-wide significance cut-off *P* = 5*10^–8^. Genomic risk loci with their corresponding locus lead SNPs were identified with FUMA v1.3.6a. All candidate SNPs are shown in grey, locus lead SNPs are colour-coded as follows: (grey circle) candidate SNPs; (dark red circle) novel genomic risk locus identified in the current study, with no previously reported candidate SNPs; (orange circle) genomic risk locus with a previously reported locus lead SNP; (yellow circle) genomic risk locus with a previously reported SNP in strong linkage disequilibrium (LD) with the locus lead SNP at r^2^ ≥ 0.6; (cyan circle) genomic risk locus with previously reported other candidate SNP. SNPs identified for allometric body-shape indices in the current study were matched against SNPs reported in the NHGRI-EBI GWAS Catalog^11^ (https://www.ebi.ac.uk/gwas/home, accessed on 07/04/2021) in association with the corresponding traditional body-shape index (with or without adjustment for body mass index, BMI), i.e. the waist-to-hip ratio for WHI_UKB_ (catalogue sets EFO_0004343, EFO_0007788, EFO_0004302); waist circumference for ABSI_UKB_ (EFO_0004342, EFO_0007789, EFO_0004302); hip circumference for HI_UKB_ (EFO_0005093, EFO_0008039, EFO_0004302).

Approximately two thirds of the independent significant SNPs but less than one fifth of the corresponding genomic risk loci associated in our study with WHI_UKB_ were novel, i.e. they have not been reported in the NHGRI-EBI GWAS Catalog^11^ in association with WHR or WHR_adj_BMI (Table 1, Supplementary Table S3). Nevertheless, over two thirds of the independent significant SNPs and one third of the corresponding genomic risk loci associated with ABSI_UKB_ were novel, i.e. not previously reported in association with WC or WC_adj_BMI. Further, most of the independent significant SNPs and over two thirds of the corresponding genomic risk loci associated with HI_UKB_ were novel, i.e. not previously reported in association with HC or HC_adj_BMI. It was notable, however, that many of the novel highest-ranked lead SNPs associated with allometric body-shape indices were in strong linkage disequilibrium (LD at r^2^ ≥ 0.6) with variants previously reported in association with the corresponding traditional body-shape index, which in our study showed lower significance (Fig. 1). Exceptions were the novel highly ranked lead SNPs associated with HI_UKB_ in men, *rs998584* in the *VEGFA* locus and *rs6066114* in the *EYA2* locus, which did not include in their clumps variants previously reported in association with HC or HC_adj_BMI, while for *rs113733630* in the *TFAP4* locus, the previously reported variant was not in the same LD block (Supplementary Table S2).

**Figure 2:**
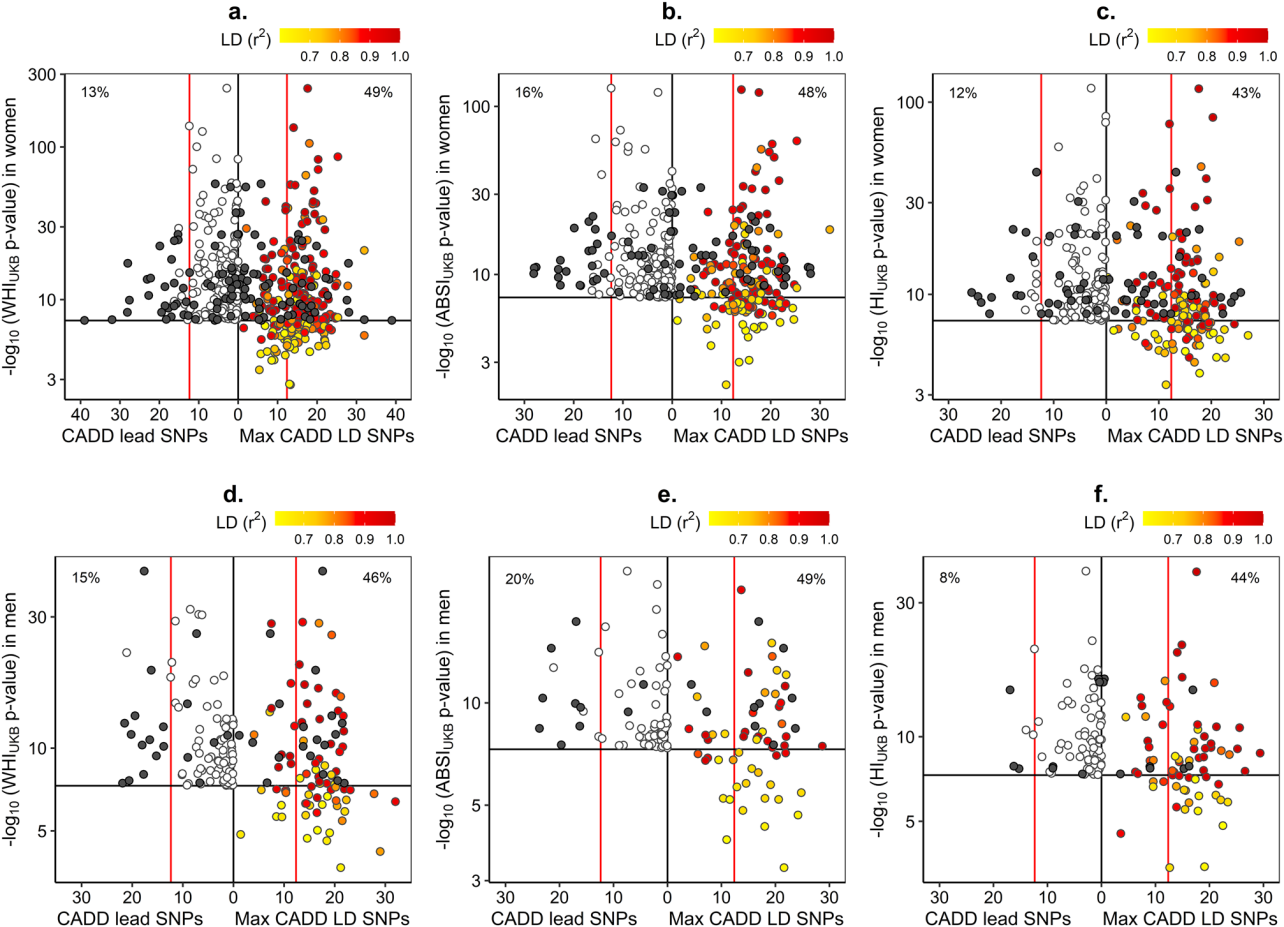
Deleteriousness (CADD) score of locus lead SNPs identified for allometric body-shape indices. (**a**) waist-to-hip index (WHI_UKB_) calibrated for UK Biobank women (n = 282 genomic risk loci with the corresponding locus lead SNPs); (**b**) a body shape index (ABSI_UKB_) calibrated for UK Biobank women (n = 200); (**c**) hip index (HI_UKB_) calibrated for UK Biobank women (n = 171); (**d**) WHI_UKB_ for UK Biobank men (n = 97); (**e**) ABSI_UKB_ for UK Biobank men (n = 65); (**f**) HI_UKB_ for UK Biobank men (n = 75). CADD-Combined Annotation Dependent Depletion; LD-linkage disequilibrium; SNP-single nucleotide polymorphism; red vertical lines-recommended cut-off 12.37 for CADD (the higher the more deleterious)^12^; horizontal line-genome-wide significance cut-off (*P* = 5*10^–8^); left-hand side-CADD for the locus lead SNP of each genomic risk locus, with the proportion above the cut-off; right-hand side-candidate SNPs in strong LD with the locus lead SNP (colour-coded according to r^2^ ≥ 0.6), showing the maximum CADD within the LD block, plotted with the corresponding significance on the y-axis; P_WHI / ABSI / HI_-*P*-values for body-shape indices, derived from BOLT-LMM infinitesimal models; (black circle)-marks the locus lead SNP when this is showing the maximum CADD within the corresponding LD block; percentages (top corners)-percentage above the cut-offs for both, CADD and genome-wide significance (all differences between left-hand side and right-hand side proportions were significant at *P* < 0.0001 when compared with Fisher’s exact test, except for ABSI_UKB_ in men (*P* = 0.0008).

The deleteriousness Combined Annotation Dependent Depletion (CADD) score was above the recommended cut-off 12.37^12^ for 8% to 20% of the locus lead SNPs associated with allometric body-shape indices (Fig. 2). Nevertheless, almost half of all locus lead SNPs were in strong LD with a variant with genome-wide significance showing higher CADD than the cut-off.

Following recent reports of a fine-scale population structure in UK Biobank^13^, we checked matches with the SNPs reported in association with birth location. Only *rs1805007* (*MC1R*), reported in association with height, was identified in our study as a candidate SNP and was included in the LD block of locus lead SNPs for WC_adj_BMI and HC_adj_BMI in women and men (lowest *P* = 4.4*10^–11^). Although *rs9268556* (MHC region), reported in association with forced vital capacity, was additionally identified as a candidate SNP for WHI_UKB_, ABSI_UKB_ and WHR_adj_BMI in women and HC_adj_BMI in men (lowest *P* = 2.4*10^–9^), this was not included in the LD block of a locus lead SNP.

### Sexual dimorphism in the genetic associations of allometric body-shape indices

The genetic association patterns of allometric body-shape indices differed considerably between women and men. The heritability was larger in women compared to men, with up to three times more independent significant SNPs identified in women (Table 1), when the excess of women vs men in the dataset was only approximately 20%. Some 30% of the independent significant SNPs for ABSI_UKB_ but 10% or less for HI_UKB_ showed sex differences in effect size at p_sex_ < 5*10^–6^ (Table 1). Several highly ranked variants, however, were particularly affected (Supplementary Fig. S3). Thus, prominent associations with WHI_UKB_ and ABSI_UKB_ in women but not in men showed variants in loci *COBLL1, RP11-95P13.1*, *ADAMTS9-AS2*, *CMIP* and *AC022431.2* (*C5orf67*) and variants in the region including loci *ZNF664 and FAM101*, while *KLF14* was the most prominent locus associated with HI_UKB_ in women but not in men (p_sex_ < 5*10^–28^ for all independent significant SNPs in these loci). In addition, although variants in *RSPO3* locus were the highest-ranked associated with WHI_UKB_ and HI_UKB_ in both sexes, variants in the *VEGFA* and *RSPO3* loci were associated with ABSI_UKB_ almost exclusively only in women, while variants in the *VEGFA* locus were associated with HI_UKB_ mainly in men (Supplementary Fig. S3).

### Gene-level associations with allometric body-shape indices

We examined gene-level associations with Multi-marker Analysis of GenoMic Annotation (MAGMA) employed in FUMA, which uses the association statistics of all SNPs included in a gene (see Methods for details).

**Figure 3:**
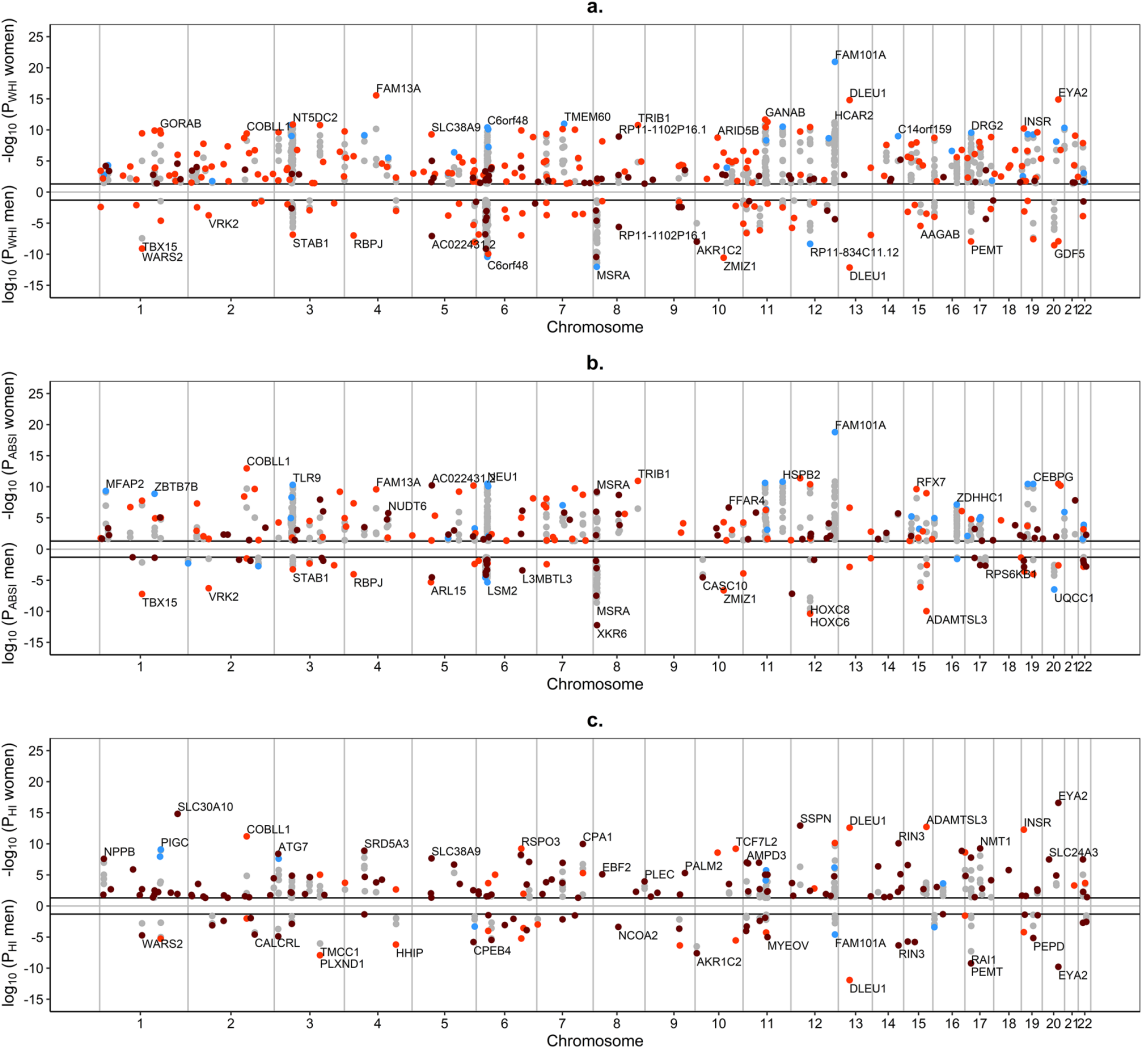
Miami plots of significant genes identified for allometric body-shape indices in women and men. (**a**) waist-to-hip index calibrated for UK Biobank participants (**WHI**_**UKB**_); (**b**) a body shape index calibrated for UK Biobank participants (ABSI_UKB_); (**c**) hip index calibrated for UK Biobank participants (HI_UKB_); P-*P*-values were derived from MAGMA v1.08 employed in FUMA v1.3.6a and were adjusted with Bonferroni correction for 19,088 protein-coding genes; SNP-single nucleotide polymorphism; horizontal lines-correspond to *P* = 0.05 after Bonferroni correction. Significant genes within 250 kb window were consolidated in genomic risk regions represented by a lead gene. All significant genes are shown in grey, lead genes are colour-coded as follows: (grey circle)-significant genes; (dark red circle)-novel genomic risk region identified in the current study, with no previously reported significant gene; (orange circle)-genomic risk region with a previously reported lead gene; (cyan circle)-genomic risk region including a previously reported significant gene (not the lead gene). Genes identified for allometric body-shape indices in the current study were matched against genes reported in the NHGRI-EBI GWAS Catalog^11^ (https://www.ebi.ac.uk/gwas/home, accessed on 07/04/2021) in association with the corresponding traditional body-shape index (with or without adjustment for body mass index, BMI), i.e. the waist-to-hip ratio for WHI_UKB_ (catalogue sets EFO_0004343, EFO_0007788, EFO_0004302); waist circumference for ABSI_UKB_ (EFO_0004342, EFO_0007789, EFO_0004302); hip circumference for HI_UKB_ (EFO_0005093, EFO_0008039, EFO_0004302).

Similar to SNP-based analyses, a larger number of non-overlapping genomic risk regions were associated with WHI_UKB_ (237 in women, 83 in men) compared to ABSI_UKB_ (158 in women, 61 in men) and HI_UKB_ (136 in women, 52 in men) and the proportions of novel gene-based regions were comparable to the proportions of novel SNP-based loci (Table 1). There were, however, differences in the lead genes (Fig. 3). The highest-ranked region for WHI_UKB_ and ABSI_UKB_ in women was *FAM101A* and for WHI_UKB_ and HI_UKB_ in men was *DLEU1*. Prominent novel genes with their corresponding genomic risk regions were *XKR6* for ABSI_UKB_ and *ERI1* for WHI_UKB_ and ABSI_UKB_ in men, *EYA2* for HI_UKB_ in both women and men, *SLC30A10* and *SSPN* for HI_UKB_ in women and *PEMP* and *AKR1C2* for HI_UKB_ in men (Supplementary Table S4). Although few independent significant SNPs had previously been reported in association with cancer (or were in strong LD with previously reported SNPs, Supplementary Fig. S4), one third of the lead genes had previously been reported in association with cancer (Table 1), including the prominent lead genes *DLEU1 and ZMIZ1* and a notable cluster of significant genes in the region of *NEU1* on chromosome 6 (Supplementary Fig. S5).

**Figure 4:**
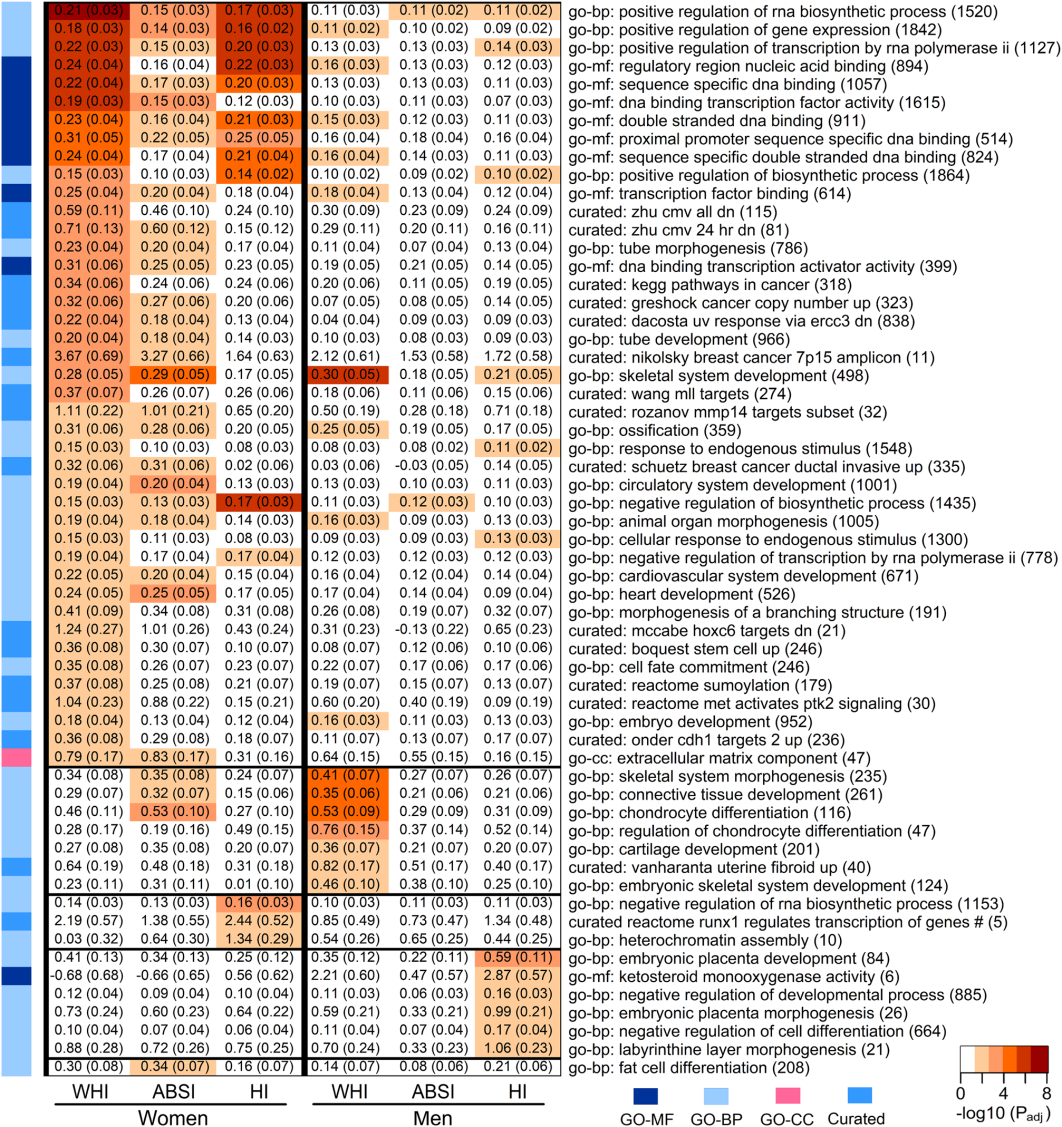
Gene sets associated with allometric body-shape indices. ABSI-a body shape index calibrated for UK Biobank participants (ABSI_UKB_); Currated-curated gene set; GO-BP-gene ontology biological process; GO-CC-gene ontology cellular component; GO-MM-gene ontology molecular function; HI-hip index calibrated for UK Biobank participants (HI_UKB_); WHI-waist-to-hip index calibrated for UK Biobank participants (WHI_UKB_); #-reactome runx1 regulates transcription of genes involved in wnt signaling. The figure includes gene sets identified using competitive gene-set analysis with MAGMA v1.08 employed in FUMA v1.3.6a as significant for at least one of the allometric indices in women or men with adjusted P_adj_ < 0.05, incorporating Bonferroni correction for multiple comparisons for 15,485 gene sets. Gene sets with P_adj_ < 0.05 for WHI_UKB_ in women were sorted in descending order of P_adj_, for WHI_UKB_ in women, then the remaining gene sets with P_adj_ < 0.05 for WHI_UKB_ in men were sorted in descending order of P_adj_ for WHI_UKB_ in men, then the remaining gene sets with P_adj_ < 0.05 for HI_UKB_ in women were sorted in descending order of P_adj_ for HI_UKB_ in women and last, the remaining gene sets with P_adj_ < 0.05 for HI_UKB_ in men were sorted in descending order of P_adj_ for HI_UKB_ in men, leaving a single gene set associated only with ABSI_UKB_ in women. The horizontal lines mark the start of the next sorting criterion. White cells are gene sets with P_adj_ ≥ 0.05 for the corresponding body-shape index.

Competitive gene-set analysis showed associations of allometric body-shape indices with gene sets related to embryonal morphogenesis and organogenesis, regulation of DNA binding, gene expression, biosynthetic processes and cell differentiation, transcription factor complexes, extracellular matrix component, circulatory system development, skeletal system development, ossification and chondrocyte differentiation and several cancers, with only a single gene set related to fat cell differentiation, which was associated with ABSI_UKB_ in women (Fig. 4). A larger number of gene sets showed significant associations with WHI_UKB_ and ABSI_UKB_ in women (42 and 29, correspondingly) compared to men (16 and 2) but a similar number were associated with HI_UKB_ (14 in women, 12 in men), although with only three overlapping.

**Figure 5:**
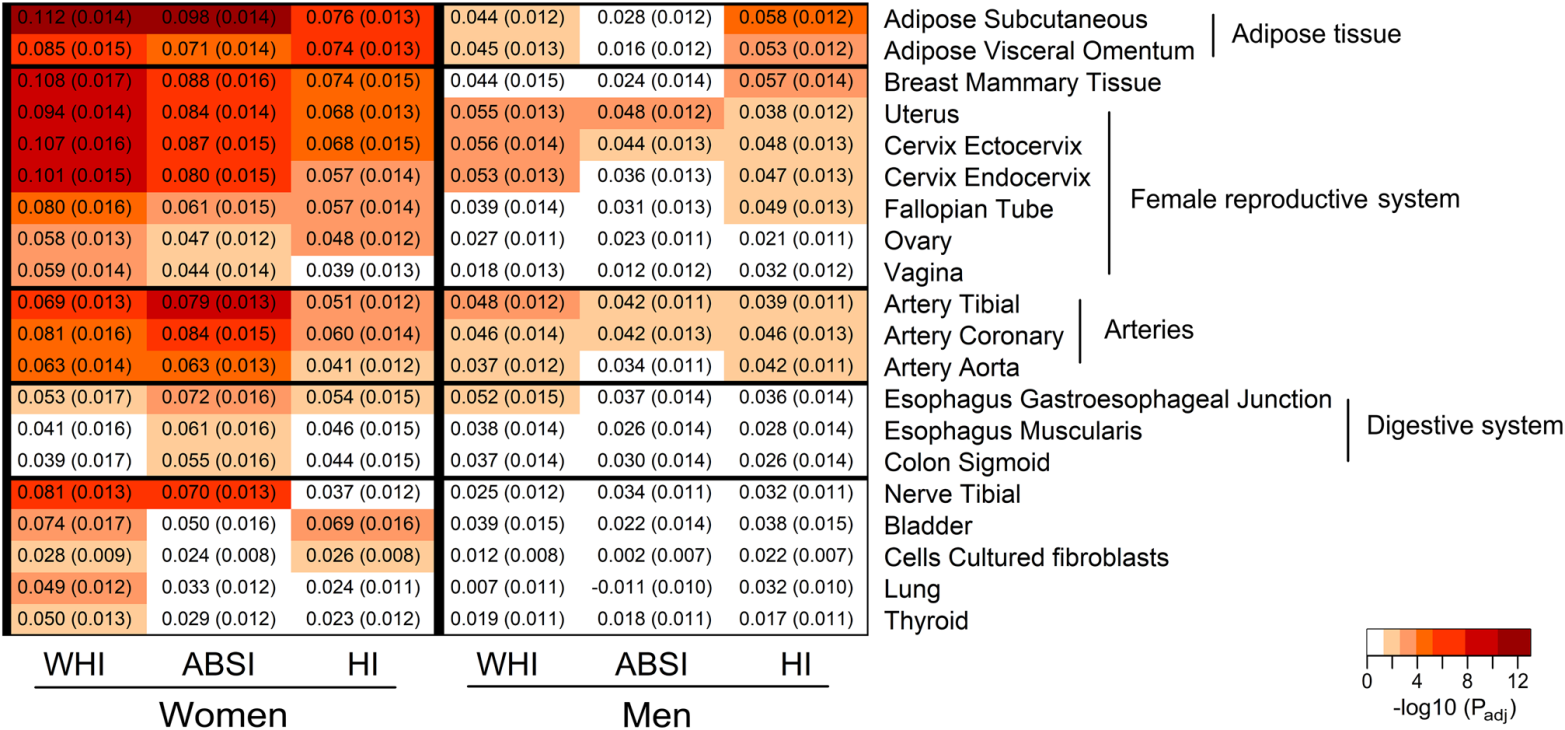
eQTLs associated with allometric body-shape indices. ABSI-a body shape index calibrated for UK Biobank participants (ABSI_UKB_); eQTLs-expression Quantitative Trait Loci; HI-hip index calibrated for UK Biobank participants (HI_UKB_); WHI-waist-to-hip index calibrated for UK Biobank participants (WHI_UKB_). The figure includes specific tissues from the Genotype-Tissue Expression (GTEx) v8.0 database, queried for associations with eQTLs with gene-property analysis, which were identified by MAGMA v1.08 employed in FUMA v1.3.6a as significant for at least one of the allometric indices in women or men, i.e. with adjusted P_adj_ < 0.05, incorporating Bonferroni correction for multiple comparisons for 54 tissues. White cells are tissues with P_adj_ ≥ 0.05 for the corresponding body-shape index.

Associations with expression Quantitative Trait Loci (eQTL) were significant in both women and men for adipose tissue (subcutaneous and visceral omentum), arteries (tibial, coronary and aorta) and, unexpectedly, for female reproductive organs (breast mammary tissue, uterus, ectocervix, endocervix and fallopian tube) and, in women only, also for ovary and vagina (Fig. 5).

### Comparison between allometric and traditional body-shape indices

**Figure 6:**
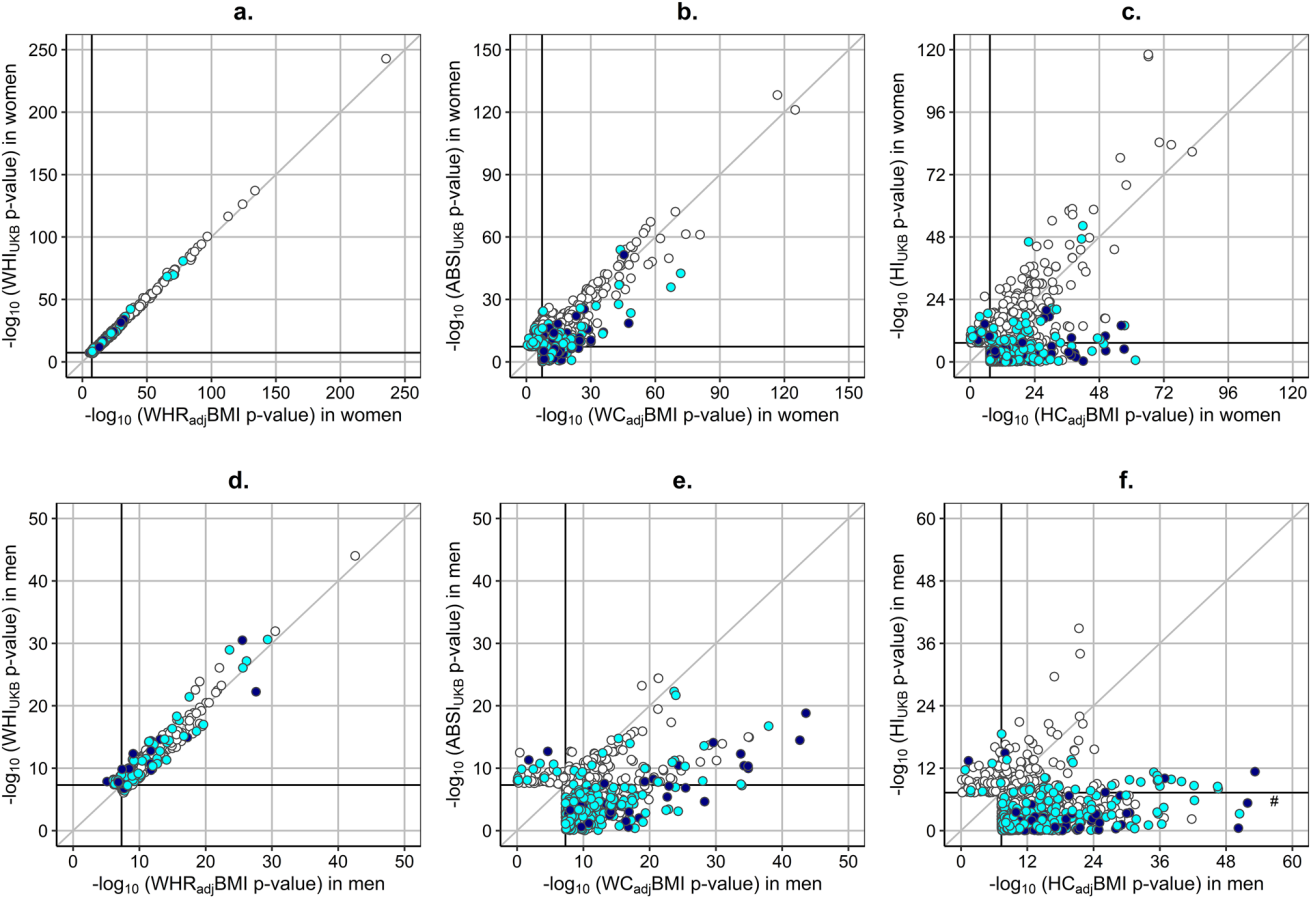
Significance ranking of independent significant SNPs: comparison between pairs of traditional and allometric body-shape indices. (**a**) independent significant SNPs (at r^2^ < 0.6 within 1 Mb window) for WHI_UKB_ (n = 1,988) or WHR_adj_BMI (n = 1,959) in women; (**b**) independent significant SNPs for ABSI_UKB_ (n = 1,273) or WC_adj_BMI (n = 1,586) in women; (**c**) independent significant SNPs for HI_UKB_ (n = 814) or HC_adj_BMI (n = 2,008) in women; (**d**) independent significant SNPs for WHI_UKB_ (n = 617) or WHR_adj_BMI (n = 571) in men; (**e**) independent significant SNPs for ABSI_UKB_ (n = 266) or WC_adj_BMI (n = 842) in men; (**f**) independent significant SNPs for HI_UKB_ (n = 222) or HC_adj_BMI (1,400) in men. Association statistics p-values were derived from BOLT-LMM infinitesimal models. ABSI_UKB_-a body shape index calibrated for UK Biobank participants; BMI-body mass index; HC_adj_BMI-hip circumference adjusted for BMI; HI_UKB_-hip index calibrated for UK Biobank participants; SNP-single nucleotide polymorphism; WC_adj_BMI-waist circumference adjusted for BMI; WHR_adj_BMI-waist-to-hip ratio adjusted for BMI; WHI_UKB_-waist-to-hip index calibrated for UK Biobank participants. Colour scale-colour-marked were only SNPs reported as associated with height in the NHGRI-EBI GWAS Catalog^11^ (https://www.ebi.ac.uk/gwas/home, accessed on 07/04/2021), i.e. included in catalogue sets EFO_0004339 or EFO_0004302; (navy circle)-independent significant SNP reported in association with height; (cyan circle)-independent significant SNP in strong LD (at r^2^ ≥ 0.6) with a SNP reported in association with height.

On the one hand, WHI_UKB_ and WHR_adj_BMI were very strongly phenotypically correlated with each other and both were uncorrelated with height or BMI (Supplementary Table S5). Correspondingly, the ranking of independent significant SNPs associated with them was in excellent agreement, especially in women (Fig. 6a,d, Supplementary Fig. S6), with only some 5% showing differences in effect size at p_difference_ < 5 * 10^–6^ (Table 1, Supplementary Fig. S7 for women, Supplementary Fig. S8 for men), mainly for variants previously reported in association with height (Supplementary Fig. S9 for women, Supplementary Fig. S10 for men).

On the other hand, while the allometric indices ABSI_UKB_ and HI_UKB_ were phenotypically uncorrelated with height and BMI, the traditional indices WC_adj_BMI and HC_adj_BMI were uncorrelated only with BMI. The adjustment for BMI was apparently introducing a moderate positive correlation with height, as height was only weakly correlated with unadjusted WC and HC (Supplementary Table S5). Notably, a larger number of independent significant SNPs were associated with WC_adj_BMI and HC_adj_BMI, with half or more of them showing significant differences in effect size compared to their allometric counterparts ABSI_UKB_ and HI_UKB_ (Table 1, Supplementary Fig. S7, Supplementary Fig. S8), except for WC_adj_BMI and ABSI_UKB_ in women, which showed more similarity (Fig. 6b). The main differences between WC_adj_BMI and ABSI_UKB_ (Fig. 6b,e) and between HC_adj_BMI and HI_UKB_ (Fig. 6c,f) concerned independent variants which were either previously reported in association with height or were in strong LD with variants reported in association with height (Supplementary Fig. S9, Supplementary Fig. S10). Half or more of the locus lead SNPs for WC_adj_BMI in men and for HC_adj_BMI in men and women had previously been associated with height, compared to less than one third for ABSI_UKB_ and HI_UKB_ (Table 1). More comparable proportions of lead genes (with gene-level significance) had previously been reported in association with height (less than half for allometric indices, Supplementary Fig. S11, and more than half for traditional indices), but the absolute number of lead genes identified for traditional indices was considerably larger (Table 1).

## Discussion

Our study presents the first GWAS of allometric body-shape indices, performed separately in women and men with white British ancestry, and a comparison with traditional body-shape indices. One third of the genomic risk loci associated with ABSI_UKB_ and over two thirds of the genomic risk loci associated with HI_UKB_ in our study were novel. Genetic associations were sexually dimorphic, with fewer independent variants identified in men compared to women. The highest-ranked independent variant for WHI_UKB_, ABSI_UKB_ in women and HI_UKB_ was *rs72959041* in the *RSPO3* locus. WHI_UKB_, combining WC and HC, showed higher sensitivity to detect genetic associations compared to the regional indices ABSI_UKB_ and HI_UKB_, based individually on WC or HC. The genetic association patterns of WHI_UKB_ and WHR_adj_BMI were very similar. ABSI_UKB_ and HI_UKB_ showed fewer genetic associations compared correspondingly to WC_adj_BMI and HC_adj_BMI, which were associated with a larger proportion of variants previously reported in association with height.

Our study highlights the importance of the *RSPO3* locus for body shape and fat distribution in individuals with white British ancestry and potentially in individuals with white ethnic background. RSPO3 protein, together with wingless-type WNT proteins, activate the canonical WNT/β-catenin pathway, which plays a depo-specific role in regulating locally adipocyte hyperplasia, hypertrophy and browning^14^. *RSPO3* shows differential expression in adipose depots, with the highest expression in visceral, intermediate in abdominal subcutaneous and lowest in gluteal subcutaneous adipose tissue^15^. *RSPO3* additionally promotes angioblast specification and vascular development^16^, plays a key role throughout life in maintaining the structural zonation and the replenishment of damaged cells in the adrenal glands^17^, regulates the renewal and differentiation of stem cells^18^, and contributes to cancer development and progression^19,20^. Genetic variants in the *RSPO3* locus associated with abdominal obesity have additionally been associated with dyslipidaemia^21^, thus relating the *RSPO3* gene to the metabolic syndrome. The highest-ranked *RSPO3* variants, *rs72959041* (in women) and *rs577721086* (in men), which are in strong linkage disequilibrium, are specific to European populations^22^ and have previously been reported in association with WHR_adj_BMI^3,4,23^. The active variant, however, may be *rs577721086*, as this is located in the attachment site of CCCTC‑binding factor, which acts as a gene repressor, insulator or activator, but also regulates the chromatin structure and enables inter-chromosomal interactions^24^.

Although the genetic associations of body-shape indices are interpreted traditionally from the perspective of the adipose tissue and insulin resistance^25^, highly ranked variants associated with body shape are located in genes coding transcription factors, receptors and enzymes involved in morphogenesis, embryonal developmental, cell proliferation and cell survival, which have additionally been linked to various cancers. Thus, in addition to the RSPO3 proteins, *HMGA1* and *TFAP4* activate the WNT/β-catenin pathway, stimulate cell migration and invasion and promote cancer progression^26,27^. *VEGFs,* which are upregulated by hypoxia, are key factors for tumour-associated angiogenesis, tissue infiltration and metastasis^28^. *PLXND1*, which is involved in angiogenesis and is upregulated by *VEGFs*, has a constitutively low expression in adult tissues but is overexpressed in cancer tissues and their vasculature^29^. *KLF14* is associated with insulin resistance, dyslipidaemia, type 2 diabetes and a female-specific shift of body fat from gynoid to abdominal stores, in agreement with the hip-specific association in women found in our study. In mice, adipocyte-specific deletion of *Klf14* results in similar metabolic effects, while *Klf14* knockout results in spontaneous tumorigenesis^30^. *TBX15* belongs to a family of transcription factors regulating differentiation, proliferation, tissue integrity and epithelial-mesenchymal transition, which are relevant to cancer development and metastasis^31^. Overexpression, mutations and epigenetic silencing of *ADAMTS* genes have been reported in different tumours^32^. *SLC30A10* is a zinc transporter maintaining zinc homeostasis, which when dysregulated can result in cancer initiation and progression^33^. *FGFR4* plays a critical role in embryonic development, tissue repair, tumour angiogenesis and progression^34^. EYA1-4 proteins can influence tumour progression through several mechanisms^35^. Homeobox (*HOX*) genes code transcription factors with DNA-binding activity, which regulate the formation of axial patterns and body shape during embryogenesis and have been implicated in cancer development^36^. *COBLL1* is the ancestor of a family of proteins involved in morphogenesis and embryonal patterning in organisms with axial symmetry and is upregulated in castrate-resistant prostate cancer with poor prognosis^37^. Upregulation of *DLEU1* can also promote tumorigenesis^38^. A question, therefore, emerges whether excess abdominal fat mediates the development of cancers associated with abdominal obesity^39^, or abdominal obesity and cancer are parallel outcomes with shared genetic predisposition. Although studies exploring a causal relationship between body shape and cancer are limited, Mendelian randomisation based on traditional body-shape indices has already provided evidence for causal positive associations of WHR or WHR_adj_BMI with colorectal cancer^40,41^ and renal cell carcinoma^42^ but an inverse association with breast cancer^43^.

Allometric body-shape indices showed sexually dimorphic genetic association patterns, like traditional body-shape indices^3,4,10,44^, with an overall lower heritability in men. There were, however, fewer genetic associations and with lower significance in men then would correspond proportionally to the slightly smaller sample size compared to women, as noted for traditional indices^3^. In contrast height, which is also phenotypically dimorphic between sexes, shows considerably larger heritability than body-shape indices and a closer agreement of the genetic association patterns between women and men^45^. This raises the question, how women and men differ with respect to the regulatory factors determining fat distribution and body shape.

Sex steroid hormones play a central role in the regulation of fat distribution. Oestrogens counter fat accumulation in the abdominal area and favour fat accumulation in the gluteofemoral area^46^. Oestrogen levels in blood^47^ and the expression of ERα in the abdominal area^48^ decrease after the menopause, which permits a functional androgen dominance and the development of android type obesity in post-menopausal women^49^. Nevertheless, age-related differences in the genetic associations have been reported only for BMI and not for WHR_adj_BMI^10^. In contrast, testosterone levels in women remain lower than in men at all ages^50^ and are comparable between pre- and post-menopausal women, as ovarian androgen production is largely maintained long after the menopause^51^. The stress hormone cortisol, regulated by the hypothalamic–pituitary–adrenal (HPA) axis, is also closely involved in the regulation of fat distribution, with chronic cortisol excess resulting in the development of visceral adiposity and the metabolic syndrome^52^. Notably, the HPA axis shows a sexually dimorphic activity, with stronger responses to stimulation and suppression in women compared to men^53,54^ and increased responses to HPA stimulation in men after suppression of gonadal testosterone production^55^. Abdominal obesity also influences HPA responsiveness, with larger WHR associated with a stronger response to HPA stimulation^56^ and a weaker response to HPA suppression^53^. Animal studies provide further evidence for a sexually dimorphic adrenal function, suggesting that androgens rather than oestrogens are important for adrenal regulation^57^. Furthermore, absence of the androgen receptor in mice results in downregulation of the glucocorticoid receptor in the pituitary gland, adrenal hypertrophy and glucocorticoid overproduction^58^. Thus, the higher HPA responsiveness in women, enabled by constitutively lower testosterone levels, may engage a more complex regulatory network and present more opportunities for genetic polymorphisms to act as rate-limiting steps in fat distribution, potentially explaining the pronounced sexual dimorphism in the heritability and the genetic association patterns of body-shape indices^4,45^.

Despite the genetic and phenotypic differences in body-shape between women and men, the eQTL associations of genes associated with body-shape indices were in line with an involvement of adipose and vascular tissues, but also intriguingly of female reproductive organs in men as well as in women. This apparently paradoxical association in men may not be completely illogical, as female reproductive organs would be responsive to oestrogen-activated pathways and the formation of the Mullerian duct, from which the uterus and its adnexa are derived, is regulated by WNT signalling pathways^59^, which may have pleiotropic roles.

We have previously demonstrated the need to adjust body-shape indices for BMI prior to using them in statistical models examining phenotypic associations^9^. We have now shown that it is also essential to account correctly for height, in order to avoid an “over-adjustment” arising from constraining the relationship between weight and height to a fixed proportion in BMI. In the case of WC_adj_BMI and HC_adj_BMI, the adjustment for BMI does not reflect correctly the scaling of WC or HC with height and introduces phenotypic and genetic associations with height. When WC and HC are adjusted for height, in addition to BMI or weight, as in the allometric indices ABSI_UKB_ and HI_UKB_, the relationship between weight and height is unconstrained and the correlation of WC and HC with height, as well as with BMI, is minimised. In the case of WHR_adj_BMI, the phenotypic and genetic association patterns were very similar to the allometric counterpart WHI_UKB_, despite the lack of additional adjustment for height, because the scaling coefficients for weight and height in individuals with white British ancestry were in proportion 1:2, similar to BMI. Nevertheless, given the large ethnic variability in body shape, this may not be universally applicable to other ethnicities. It would thus be advisable to use body-shape indices adjusted for height, as well as for BMI or weight, in order to minimise correlations with body size. Evaluating correctly the pleiotropic contributions is particularly important as many variants and genes associated with allometric body-shape indices are apparently related to growth and regulatory factors and a sizeable proportion of them have previously been reported in association with height. A mechanistic parallel with a pathway affecting either height or regional size when dysregulated in different periods of life could be drawn with growth hormone signalling. Growth hormone excess in adolescents is associated with increased height but an excess in adults, after epiphyseal closure, is associated with regional enlargement of the hands, feet and the face, i.e. acromegaly. Growth hormone signalling is further related to metabolic alterations and some cancers^60^. A similar principle could be relevant to other growth or regulatory factors.

Our study benefited from a large sample size, anthropometric measurements performed by trained personnel according to standardised protocols and access to established bioinformatics pipelines. There were, however, several limitations. It should first be acknowledged, that the UK Biobank cohort is not representative of the UK population at the time of recruitment and is subject to a healthy volunteer bias which should be considered when interpreting the findings^61^. In addition, we lacked a validation cohort of similar ethnicity to the discovery dataset, although this would affect mainly the validity of associations with lower and borderline significance, while the highest-ranked genetic polymorphisms showed very strong and convincing associations. We also lacked datasets of comparable size including individuals with different ethnic backgrounds, as less than 5% of UK Biobank participants reported black or Asian ethnic background. Further, there were no reliable imaging measures of body composition (i.e. from dual-emission X-ray absorptiometry scans or magnetic resonance imaging) with comparable sample size. Furthermore, we did not perform a meta-analysis of waist and hip indices, since there are no other GWAS of ABSI and HI and we have already argued that WC_adj_BMI and HC_adj_BMI are not reliable body-shape indices. It would be useful, however, to consider a multi-trait meta-analysis approach^62^ when studies of other ethnicities are available. We did not specifically adjust our analyses for geographical location, which has been highlighted as a source of residual confounding of associations with BMI, bioelectric impedance fat mass measurements and height in UK Biobank^13^, as we minimised the number of adjustment variables to avoid introducing collider bias. There were, however, no matches of the reported variants related to geographical location with the main variants identified in our study, hence a confounding from fine-scale population structure is unlikely. Further, we could not perform mechanistic investigations linking genetic polymorphisms to adrenal function or cancer, which were beyond the scope of our study. Lack of Mendelian randomisation analysis, which may support a causal effect of the identified variants and genes on cancer and may further clarify the relationships in the network of mediators^63^, is clearly a limitation. This, however, was also beyond the scope of the current study. Due to the large heterogeneity of cancers, associations would need to be consider separately for each individual cancer location and for each major histological type and the question of potential pleiotropy, which is very likely, would need to be addressed in detail. It should last be noted, that although examining ABSI_UKB_ and HI_UKB_ enabled the identification of variants associated exclusively with the abdominal or the gluteofemoral regions, these associations may be determined by features other than fat accumulation, e.g. the status of lean mass in the gluteofemoral region. It would therefore be important to examine the association patterns of genetic variants identified for anthropometric indices with measures of body compositions, which would become available in the future for a larger part of UK Biobank participants.

In conclusion, in the first GWAS of allometric body-shape indices, we have identified novel genetic variants and genes and have obtained unbiased association statistics for individuals with white British ancestry, which would inform future studies of fat distribution, body shape and the disorders associated with them. The highest-ranked genes associated with body-shape indices have previously been associated in mechanistic studies with adrenal cell renewal, vascularisation and cancer development and progression, in addition to their functions in adipose tissue. The comparison of allometric and traditional body-shape indices demonstrated that adjustment of body-shape indices for height, as well as for BMI or weight, is warranted to avoid associations with height, which were more pronounced for WC_adj_BMI and HC_adj_BMI. Differences between allometric and traditional body-shape indices with respect to their phenotypic and genetic associations were minimal when accounting correctly for height.

## Methods

### Study participants

UK Biobank is a prospective cohort with an ongoing follow-up, including 502,543 participants. Recruitment and data collection have previously been described^64,65^. We excluded in total 95,846 participants. Exclusions were determined by a lack of genetic data or withdrawn consent (n = 15,229); outliers for heterozygosity or missing genotyping rate, or sex chromosome aneuploidy, or a mismatch between genetic and self-reported sex (n = 843); age below 40 or above 70 years when attending an assessment centre at baseline (n = 14); missing weight, height, waist or hip circumference measurements (n = 2,097); or pregnancy at baseline (n = 115). We further restricted the selection to participants with white British ancestry, defined by UK Biobank according to their genetic characteristics (excluded n = 77,548). The final dataset comprised 406,697 participants.

### Genotyping and imputation

Genotyping, imputation and quality control were performed centrally by UK Biobank and have previously been described^65^. Two genotyping arrays were used: Applied Biosystems UK BiLEVE Axiom Array (~ 50,000 participants) and a closely related Applied Biosystems UK Biobank Axiom Array (~ 450,000 participants).

### Outcome measures and indices

We converted all anthropometric measurements to body-shape indices with adjustments either for BMI in linear models, or for weight and height in log-linear models. BMI was calculated by dividing weight (kg) by squared height (m).

For traditional body-shape indices, we used residuals of linear models regressing each of WC (cm), HC (cm) or WHR on BMI (WHR_adj_BMI, WC_adj_BMI, HC_adj_BMI). For the corresponding allometric body-shape indices, we used ABSI and HI, and created a new waist-to-hip index (WHI), in order to complete the set. The published formulas for ABSI and HI have previously been derived for participants in the National Health and Nutrition Examination Survey (NHANES)^5,6^:

$${\mathbf{ABSI}} = {\text{WC}}*{\text{Weight}}^{{{-}2/3}} *{\text{Height}}^{5/6}$$

$${\mathbf{HI}} = {\text{HC}}*{\text{Weight}}^{{{-}0.482}} *{\text{Height}}^{0.310}$$

To avoid phenotypic correlations between anthropometric indices arising from differences in the anthropometric characteristics of UK Biobank and NHANES participants and to enable comparability with traditional residual methods, we calibrated ABSI and HI, as well as WHI, for UK Biobank participants (ABSI_UKB_, HI_UKB_ and WHI_UKB_). We derived the power coefficients from linear models regressing each of log-transformed WC (cm), HC (cm) or WHR on log-transformed weight (kg) and height (m) measured at baseline:

$$\log \left( {Measure} \right) \sim \beta*\log \left( {Weight} \right) + \gamma*\log \left( {Height} \right)$$

and generated allometric body-shape indices according to the general formula:

$$Index = Measure*Weight^{ - \beta } *Height^{ - \gamma }$$

where *β* and *γ* are the regression coefficients for weight and height (Supplementary Table S6). We included ABSI and HI calculated with the published regression coefficients from NHANES only for the phenotypic comparisons.

The absolute values of the power coefficients for weight and height for WHR_UKB_ were close to a ratio of 1:2 in both men and women, which corresponds to the relationship weight/height^2^ in BMI (Supplementary Table S6). Therefore, in analogy to ABSI, for which the published coefficients were derived by rounding the power coefficients obtained in NHANES to simple fractions^5^, we generated for comparison a simplified version of WHI with the formula:

$${\text{WHI}} = {\text{WHR}}*\left[ {{\text{Weight}}\left( {{\text{kg}}} \right)/{\text{Height}}^{2} \left( {{\text{cm}}} \right)} \right]^{ - 1/4}$$

As body-shape patterns show distinct differences between sexes^10^, we generated all indices and performed all statistical analyses separately for men and women. We used Blom’s method for inverse normal transformation of anthropometric indices (package ***RNOmni*** in R), as in^66^.

### Association testing

We obtained Pearson’s coefficient for partial phenotypic correlation between anthropometric indices with function ***pcor*** (package ***ppcor*** in R), adjusting for age at baseline.

We used Bayesian linear mixed-model analysis BOLT-LMM v2.3 for genome-wide association testing^67,68^, which incorporates in the statistical algorithm a correction for population stratification and thus accounts for the relatedness between UK Biobank participants. To estimate the parameters of the LMM, we used linkage disequilibrium (LD) scores from the 1000 Genomes EUR samples from individuals with European ancestry^69^ and selected a coreset with high-quality genetic variants by restricting the list of variants released by UK Biobank after the centrally performed quality control to variants with missingness < 0.015, minor allele frequency MAF > 5% and Hardy–Weinberg exact test *P* > 1*10^–6^ (Supplementary Table S7). We performed the main analyses with variants with MAF ≥ 1% and imputation quality factor INFO > 0.1. We adjusted all models for age at baseline, age squared and a binary indicator of genotyping array. Supplementary Table S8 includes quality control parameters for BOLT-LMM. We obtained heritability estimates (h^2^_g_) from BOLT-LMM, based on the genetic relationship matrix. We report association statistics based on the BOLT-LMM infinitesimal models, which use a Gaussian single nucleotide polymorphism (SNP) effect prior.

### Mapping, annotation and prioritisation of genetic variants

Individual genetic variants were defined with the chromosome, the genomic position in base pairs, the minor (alternative) allele and the major (reference) allele. For some SNPs there were more than one alternative alleles, hence the total number of genetic variants associated with a given body-shape index could be higher than the total number of SNPs. We used the SNP2GENE process of the web application Functional Mapping and Annotation (FUMA) v1.3.6a^70^ to perform positional mapping, clumping and annotation of genetic variants according to the p-values of the association statistics obtained from the corresponding BOLT-LMM infinitesimal model for variants with high imputation quality (INFO > 0.9). Independent significant SNP were defined as variants with genome-wide significance (*P* ≤ 5*10^–8^), which were in approximate linkage equilibrium with each other, at r^2^ < 0.6 within a 1 Mb window. Variants with nominal significance (*P* < 0.05) in LD with an independent significant SNP (r^2^ ≥ 0.6 within a 1 Mb window) formed the corresponding LD block of candidate SNPs. To map LD, we used the “*UKB release2b 10 k White British*” panel in FUMA. Independent significant SNPs in LD with each other (r^2^ ≥ 0.1 within 1 Mb window) were consolidated in a clump, represented by a lead SNP with the lowest p-value. Lead SNPs with corresponding clump boundaries less than 250 kb apart were merged into a genetic risk locus, represented by the locus lead SNP with the lowest p-value. All candidate SNPs were mapped to genes within a maximum distance of 1 kb (based on their genomic position) and for functional consequences (based on Ensemble genes v92) with ANNOVAR employed in FUMA^71^. We used as a measure of pathogenicity of a given variant the deleteriousness score (Combined Annotation Dependent Depletion (CADD) score) calculated in FUMA and evaluated this against the recommended cut-off of 12.37^12^. We additionally annotated the functionality of candidate SNPs with genome-wide significance with Ensembl Variant Effect Predictor (VEP) v90^72^. This maps each variant to the gene or location with the nearest protein-coding transcription start site within a window of 200 kb from the position of the examined variant.

### Gene-based association analysis

For gene-level, gene-set and gene-property analysis, we used Multi-marker Analysis of GenoMic Annotation (MAGMA) v.1.08^73^, which is employed in FUMA v1.3.6a with default settings. The reference panel for LD and the Ensemble version for assigning SNPs to genes (within a symmetric window of 1 kb from both sides) were set as described for SNP annotation and prioritisation above.

Gene analysis was based on the summary SNP statistics of all variants with INFO > 0.9 (i.e. including the complete distribution). A SNP-wide mean model was used to calculate an association statistic and p-value for each gene. Significant genes were considered those with *P* < 0.05, after applying Bonferroni correction for multiple comparisons for 19,088 identified protein-coding genes. Significant genes with boundaries within 250 kb of each other were clumped in genomic risk regions, represented by a lead gene with the lowest *P*-value.

Competitive gene-set analysis was performed as a gene-level linear regression model to test whether the genes included in each gene-set (a binary indicator) showed stronger (positive) associations with the phenotype than other genes, generating a one-sided p-value. The model was conditioned by default on gene size, gene density (reflecting LD between SNPs in the gene), the inverse of the mean MAF in the gene (to account for potential power loss in very low MAF SNPs), and the log values of the three variables^73^. FUMA v1.3.6a uses gene sets obtained from the Molecular Signatures Database (MSigDB) v7.0, including curated gene sets from online pathway databases and gene ontology (GO) terms. The significance of associations with gene sets was evaluated at *P* < 0.05, after Bonferroni correction for 15,485 examined gene sets.

The Genotype-Tissue Expression (GTEx) v8.0 database was used to perform gene-property analysis, as implemented in FUMA v1.3.6a, which examines associations with expression Quantitative Trait Loci (eQTL). This uses a similar linear regression model to gene-set analysis, but with the average log_2_ transformed gene expression values per tissue (a continuous variable), to test the (positive) relationship between highly expressed genes in a specific tissue and genetic associations represented by gene-level statistics, generating a two-sided p-value. The significance of associations with eQTLs was evaluated at *P* < 0.05, after Bonferroni correction for 54 individual tissue types.

### Match against published reports

We matched all candidate SNPs and the significant genes identified by MAGMA in FUMA against SNPs and genes previously reported in the NHGRI-EBI GWAS Catalog^11^ (accessed on 07/04/2021, https://www.ebi.ac.uk/gwas/home) in association with anthropometric indices. Novelty for a genomic risk locus was concluded if there was no match of any candidate SNP included in the locus with a SNP reported in the NHGRI-EBI GWAS Catalog in association with the corresponding traditional index. Similarly, novelty for a genomic risk region was concluded if there was no match of any significant gene included in the region with a gene reported in the NHGRI-EBI GWAS Catalog in association with the corresponding traditional index. Match with reported associations with height or cancer were compared at the level of independent significant SNPs (i.e. a match with any candidate SNP in the LD block) or at the level of an individual significant gene.

### Difference analysis

To test for sex-dimorphic effects, we calculated a t-statistic as follows:

$${\text{t}}_{{{\text{sex}}}} = \frac{{\beta_{{{\text{men}}}} - \beta_{{{\text{women}}}} }}{{\sqrt {{\text{SE}}^{2}_{{{\text{men}}}} + {\text{SE}}^{2}_{{{\text{women}}}} {-} \, 2*r_{{{\text{sex}}}} *{\text{SE}}_{{{\text{men}}}} *{\text{SE}}_{{{\text{women}}}} } }}$$

where β are the regression coefficients for a given variant in men or women, SE are the corresponding standard errors and r_sex_ is the Spearman rank correlation coefficient between the regression coefficients in men and women for all examined genome-wide variants^3,10^. We extracted the corresponding p-values (p_sex_) from a t-distribution with function ***pt*** in R. We used a similar formula to compare heritability between sexes, replacing the regression coefficients with heritability estimates in men and women.

To test for difference in effect sizes between allometric and traditional indices, we calculated, separately in women and men, t-statistics and the corresponding p_difference_ with a similar formula to the one used for sex dimorphisms:

$${\text{t}}_{{\text{allo - trad}}} = \frac{{\beta_{{{\text{allo}}}} - \beta_{{{\text{trad}}}} }}{{\sqrt {{\text{SE}}^{2}_{{{\text{allo}}}} + {\text{SE}}^{2}_{{{\text{trad}}}} {-} \, 2*r_{{\text{allo - trad}}} *{\text{SE}}_{{{\text{allo}}}} *{\text{SE}}_{{{\text{trad}}}} } }}$$

where β are the regression coefficients for a given variant for the corresponding allometric and traditional index in a pair (i.e. WHI_UKB_ and WHR_adj_BMI, ABSI_UKB_ and WC_adj_BMI, HI_UKB_ and HC_adj_BMI), SE are the corresponding standard errors and r_allo-trad_ is the Spearman rank correlation coefficient between the regression coefficients for the allometric and traditional index for all examined genome-wide variants. To test for difference in effect sizes between the allometric waist and hip indices, the parameters for allometric and traditional indices were replaced with the corresponding parameters for ABSI_UKB_ and HI_UKB_ and r_ABSI-HI_ was calculated as above.

As differences in effect sizes were considered only for independent significant SNPs identified for at least one of the two compared sexes or indices and this would vary between comparisons, we evaluated differences relative to a single universal conservative cut-off *P* < 5 × 10^–6^, which is equivalent to a Bonferroni correction for 10,000 comparisons and reflects strong evidence for association.

We used R version 3.6.1. for the management of data and results^74^.

### Ethical approval and consent to participate

This research was conducted according to the principles expressed in the Declaration of Helsinki. The UK Biobank cohort has been approved by the North West Multicenter Research Ethics Committee, UK (Ref: 16/NW/0274). Written informed consent has been obtained from all study participants. The current study was approved by the UK Biobank access management board under application 41952. Participants who had withdrawn consent by the time of the analysis were excluded from the dataset.

## Supplementary Information

Supplementary Figures and Tables.

Supplementary Table S9.

Supplementary Table S10.

Supplementary Table S11.

## Data Availability

The data supporting the findings of the study are available *to bona fide* researchers upon approval of an application to the UK Biobank (https://www.ukbiobank.ac.uk/researchers/) and a material transfer agreement. The results from FUMA with additional annotations are included in Supplementary Table S9 (candidate SNPs) and Supplementary Table S10 (all other analyses) and the gene-level analysis from MAGMA and a list of the sets and traits from the NHGRI-EBI GWAS Catalog used for matching are included in Supplementary Table S10. Summary statistics from BOLT-LMM infinitesimal models for all candidate SNPs identified in our study are included in Supplementary Table S11. The SNP2GENE output will also be made available upon publication on the FUMA website (https://fuma.ctglab.nl/) and locus lead SNPs will be uploaded to the NHGRI-EBI GWAS Catalog (https://www.ebi.ac.uk/gwas/home).

## Abbreviations

ABSI: A body shape index
ABSI_UKB_: A body shape index calibrated for UK Biobank participants
ADAMTS: A disintegrin and metalloprotease domains with thrombospondins motifs
AKR1C2: Aldo-keto reductase family 1 member C2
BMI: Body mass index
BOLT-LMM: Bayesian mixed-model association method
C5orf67: Chromosome 5 open reading frame 67
CADD: Combined Annotation Dependent Depletion
CASC20: Cancer susceptibility 20
CMIP: C-Maf inducing protein
COBLL1: Cordon-bleu WH2 repeat protein like 1
DLEU1: Deleted in lymphocytic leukemia 1
eQTL: Expression Quantitative Trait Loci
ERα: Oestrogen receptor α
ERI1: Exoribonuclease 1
EYA: Eyes absent homolog
FAM101A: Filamin-interacting protein (readthrough region with *ZNF664*)
FGFR4: Fibroblast growth factor receptor 4
FUMA: Functional Mapping and Annotation
GO: Gene ontology
GWAS: Genome-wide association study
HC: Hip circumference
HC_adj_BMI: Hip circumference adjusted for body mass index
HI: Hip index
HI_UKB_: Hip index calibrated for UK Biobank participants
HMGA1: High mobility group AT-hook 1
HPA: Hypothalamic-pituitary-adrenal axis
KLF14: Kruppel-like factor 14
LD: Linkage disequilibrium
MAF: Minor allele frequency
MAGMA: Multi-marker Analysis of GenoMic Annotation
MC1R: Melanocortin 1 receptor
NEU1: Neutraminidase 1
NHANES: National Health and Nutrition Examination Survey
PEMP: Palmitoylated erythrocyte membrane protein
PLXND1: Plexin D1
SLC30A10: Solute carrier family 30 member 10
SNP: Single nucleotide polymorphism
SSPN: Sarcospan
TBX15: T-box transcription factor 15
TFAP4: Transcription factor AP-4
VEGFA: Vascular endothelial growth factor-A
VEP: Ensembl Variant Effect Predictor
WC: Waist circumference
WC_adj_BMI: Waist circumference adjusted for body mass index
WHI: Waist-to-hip index
WHI_UKB_: Waist-to-hip index calibrated for UK Biobank participants
WHR: Waist-to-hip ratio
WHR_adj_BMI: Waist-to-hip ratio adjusted for body mass index
WNT: Wingless-type
XKR6: XK related 6
ZMIZ1: Zinc-finger MIZ-type containing 1
ZNF664: Zinc finger protein 664

## References

[1] Hill JH, Solt C, Foster MT (2018). Obesity associated disease risk: the role of inherent differences and location of adipose depots. Horm. Mol. Biol. Clin. Investig..

[2] Carmienke S (2013). General and abdominal obesity parameters and their combination in relation to mortality: a systematic review and meta-regression analysis. Eur. J. Clin. Nutr..

[3] Pulit SL (2019). Meta-analysis of genome-wide association studies for body fat distribution in 694 649 individuals of European ancestry. Hum. Mol. Genet..

[4] Shungin D (2015). New genetic loci link adipose and insulin biology to body fat distribution. Nature.

[5] Krakauer NY, Krakauer JC (2012). A new body shape index predicts mortality hazard independently of body mass index. PLoS One.

[6] Krakauer NY, Krakauer JC (2016). An anthropometric risk index based on combining height, weight, waist, and hip measurements. J. Obes..

[7] Stevens CF (2009). Darwin and Huxley revisited: the origin of allometry. J. Biol..

[8] Bertoli S (2017). Association of Body Shape Index (ABSI) with cardio-metabolic risk factors: A cross-sectional study of 6081 Caucasian adults. PLoS One.

[9] Christakoudi S (2020). A Body Shape Index (ABSI) achieves better mortality risk stratification than alternative indices of abdominal obesity: results from a large European cohort. Sci. Rep..

[10] Winkler TW (2015). The influence of age and sex on genetic associations with adult body size and shape: a large-scale genome-wide interaction study. PLoS Genet..

[11] Buniello A (2019). The NHGRI-EBI GWAS Catalog of published genome-wide association studies, targeted arrays and summary statistics 2019. Nucleic Acids Res..

[12] Kircher M (2014). A general framework for estimating the relative pathogenicity of human genetic variants. Nat. Genet..

[13] Cook JP, Mahajan A, Morris AP (2020). Fine-scale population structure in the UK Biobank: implications for genome-wide association studies. Hum. Mol. Genet..

[14] Chen N, Wang J (2018). Wnt/beta-catenin signaling and obesity. Front. Physiol..

[15] Loh NY (2015). LRP5 regulates human body fat distribution by modulating adipose progenitor biology in a dose- and depot-specific fashion. Cell. Metab..

[16] Kazanskaya O (2008). The Wnt signaling regulator R-spondin 3 promotes angioblast and vascular development. Development.

[17] Vidal V (2016). The adrenal capsule is a signaling center controlling cell renewal and zonation through Rspo3. Genes. Dev..

[18] Raslan AA, Yoon JK (2019). R-spondins: multi-mode WNT signaling regulators in adult stem cells. Int. J. Biochem. Cell. Biol..

[19] Chen Z (2019). RSPO3 promotes the aggressiveness of bladder cancer via Wnt/beta-catenin and Hedgehog signaling pathways. Carcinogenesis.

[20] Tocci JM, Felcher CM, Garcia SME, Kordon EC (2020). R-spondin-mediated WNT signaling potentiation in mammary and breast cancer development. IUBMB Life.

[21] Hoffmann TJ (2018). A large electronic-health-record-based genome-wide study of serum lipids. Nat. Genet..

[22] Hunt SE (2018). Ensembl variation resources. Database.

[23] Tachmazidou I (2017). Whole-genome sequencing coupled to imputation discovers genetic signals for anthropometric traits. Am. J. Hum. Genet..

[24] Kim S, Yu NK, Kaang BK (2015). CTCF as a multifunctional protein in genome regulation and gene expression. Exp. Mol. Med..

[25] Sulc J, Winkler TW, Heid IM, Kutalik Z (2020). Heterogeneity in obesity: genetic basis and metabolic consequences. Curr. Diab. Rep..

[26] Han X, Cao Y, Wang K, Zhu G (2016). HMGA1 facilitates tumor progression through regulating Wnt/beta-catenin pathway in endometrial cancer. Biomed. Pharmacother..

[27] Song J (2018). Transcription factor AP-4 promotes tumorigenic capability and activates the Wnt/β-catenin pathway in hepatocellular carcinoma. Theranostics.

[28] Ceci C, Atzori MG, Lacal PM, Graziani G (2020). Role of VEGFs/VEGFR-1 signaling and its inhibition in modulating tumor invasion: experimental evidence in different metastatic cancer models. Int. J. Mol. Sci..

[29] Vivekanadhan S, Mukhopadhyay D (2019). Divergent roles of Plexin D1 in cancer. Biochim. Biophys. Acta Rev. Cancer.

[30] Chen X, Shi W, Zhang H (2020). The role of KLF14 in multiple disease processes. BioFactors.

[31] Papaioannou VE (2014). The T-box gene family: emerging roles in development, stem cells and cancer. Development.

[32] Cal S, López-Otín C (2015). ADAMTS proteases and cancer. Matrix Biol..

[33] Wang J, Zhao H, Xu Z, Cheng X (2020). Zinc dysregulation in cancers and its potential as a therapeutic target. Cancer Biol. Med..

[34] Liu Y (2020). Dissecting the role of the FGF19-FGFR4 signaling pathway in cancer development and progression. Front. Cell Dev. Biol..

[35] Zhou H, Zhang L, Vartuli RL, Ford HL, Zhao R (2018). The Eya phosphatase: its unique role in cancer. Int. J. Biochem. Cell. Biol..

[36] Brotto DB (2020). Contributions of HOX genes to cancer hallmarks: enrichment pathway analysis and review. Tumour Biol..

[37] Takayama KI, Suzuki T, Fujimura T, Takahashi S, Inoue S (2018). COBLL1 modulates cell morphology and facilitates androgen receptor genomic binding in advanced prostate cancer. Proc. Natl. Acad. Sci U S A.

[38] Pang B (2019). Upregulation of DLEU1 expression by epigenetic modification promotes tumorigenesis in human cancer. J. Cell Physiol..

[39] Silveira EA, Kliemann N, Noll M, Sarrafzadegan N, De Oliveira C (2020). Visceral obesity and incident cancer and cardiovascular disease: an integrative review of the epidemiological evidence. Obes. Rev..

[40] Bull CJ (2020). Adiposity, metabolites, and colorectal cancer risk: mendelian randomization study. BMC Med..

[41] Jarvis D (2016). Mendelian randomisation analysis strongly implicates adiposity with risk of developing colorectal cancer. Br. J. Cancer.

[42] Johansson M (2019). The influence of obesity-related factors in the etiology of renal cell carcinoma-a mendelian randomization study. PLoS Med.

[43] Shu X (2019). Associations of obesity and circulating insulin and glucose with breast cancer risk: a Mendelian randomization analysis. Int. J. Epidemiol..

[44] Heid IM (2010). Meta-analysis identifies 13 new loci associated with waist-hip ratio and reveals sexual dimorphism in the genetic basis of fat distribution. Nat. Genet..

[45] Zillikens MC (2008). Sex-specific genetic effects influence variation in body composition. Diabetologia.

[46] Palmer BF, Clegg DJ (2015). The sexual dimorphism of obesity. Mol. Cell. Endocrinol..

[47] Coburn SB (2019). Comparability of serum, plasma, and urinary estrogen and estrogen metabolite measurements by sex and menopausal status. Cancer Causes Control.

[48] Park Y-M, Erickson C, Bessesen D, Van Pelt RE, Cox-York K (2017). Age- and menopause-related differences in subcutaneous adipose tissue estrogen receptor mRNA expression. Steroids.

[49] Lizcano F, Guzman G (2014). Estrogen deficiency and the origin of obesity during menopause. Biomed. Res. Int..

[50] Fanelli F (2013). Revisiting hyper- and hypo-androgenism by tandem mass spectrometry. Rev. Endocr. Metab. Disord..

[51] Fogle RH, Stanczyk FZ, Zhang X, Paulson RJ (2007). Ovarian androgen production in postmenopausal women. J. Clin. Endocrinol. Metab..

[52] Pasquali, R., Vicennati, V., Cacciari, M. & Pagotto, U. The hypothalamic-pituitary-adrenal axis activity in obesity and the metabolic syndrome *Ann. N.Y. Acad. Sci.* **1083**, 111–128 (2006). 10.1196/annals.1367.009 17148736

[53] Rutters F, Nieuwenhuizen AG, Lemmens SG, Born JM, Westerterp-Plantenga MS (2010). Hypothalamic-pituitary-adrenal (HPA) axis functioning in relation to body fat distribution. Clin. Endocrinol. (Oxf.).

[54] Vicennati V (2006). Sex difference in the relationship between the hypothalamic-pituitary-adrenal axis and sex hormones in obesity. Obesity (Silver Spring).

[55] Rubinow DR (2005). Testosterone suppression of CRH-stimulated cortisol in men. Neuropsychopharmacology.

[56] Pasquali R (1993). The hypothalamic-pituitary-adrenal axis in obese women with different patterns of body fat distribution. J. Clin. Endocrinol. Metab..

[57] Grabek A (2019). The adult adrenal cortex undergoes rapid tissue renewal in a sex-specific manner. Cell Stem Cell.

[58] Miyamoto J (2007). The pituitary function of androgen receptor constitutes a glucocorticoid production circuit. Mol. Cell. Biol..

[59] Vainio S, Heikkilä M, Kispert A, Chin N, Mcmahon AP (1999). Female development in mammals is regulated by Wnt-4 signalling. Nature.

[60] Strous GJ (2020). Growth hormone receptor regulation in cancer and chronic diseases. Front. Endocrinol (Lausanne).

[61] Keyes KM, Westreich D (2019). UK Biobank, big data, and the consequences of non-representativeness. The Lancet.

[62] Wu Y (2020). Multi-trait analysis for genome-wide association study of five psychiatric disorders. Transl. Psychiatry.

[63] Hou L (2020). Exploring the causal pathway from ischemic stroke to atrial fibrillation: a network Mendelian randomization study. Mol. Med..

[64] UK Biobank Coordinating Centre: UK Biobank: Protocol for a large-scale prospective epidemiological resource. Protocol No: UKBB-PROT-09-06 (main phase, amendment one final). http://www.ukbiobank.ac.uk/wp-content/uploads/2011/11/UK-Biobank-Protocol.pdf (2007)

[65] Bycroft C (2017). Genome-wide genetic data on ~ 500,000 UK Biobank participants. bioRxiv.

[66] Sinnott-Armstrong N (2019). Genetics of 38 blood and urine biomarkers in the UK Biobank. bioRxiv.

[67] Loh PR, Kichaev G, Gazal S, Schoech AP, Price AL (2018). Mixed-model association for biobank-scale datasets. Nat. Genet..

[68] Loh P-R (2015). Efficient Bayesian mixed-model analysis increases association power in large cohorts. Nat. Genet..

[69] Abecasis GR (2012). An integrated map of genetic variation from 1,092 human genomes. Nature.

[70] Watanabe K, Taskesen E, Van Bochoven A, Posthuma D (2017). Functional mapping and annotation of genetic associations with FUMA. Nat. Commun..

[71] Wang K, Li M, Hakonarson H (2010). ANNOVAR: functional annotation of genetic variants from high-throughput sequencing data. Nucleic Acids Res..

[72] Mclaren W (2016). The ensembl variant effect predictor. Genome Biol..

[73] De Leeuw CA, Mooij JM, Heskes T, Posthuma D (2015). MAGMA: generalized gene-set analysis of GWAS data. PLoS Comput. Biol..

[74] R Core Team. R: A language and environment for statistical computing. R Foundation for Statistical Computing, Vienna, Austria. https://www.R-project.org/. (2017).

