# Supplementary material for: GWAS of allometric body-shape indices in UK Biobank identifies loci suggesting associations with morphogenesis, organogenesis, adrenal cell renewal and cancer
Source: Sci Rep. 2021 May 21;11:10688. doi: 10.1038/s41598-021-89176-6 (PMC8139988; doi:10.1038/s41598-021-89176-6)
Supplement: Supplementary file 1 — Supplementary Figures and Tables. [file 41598_2021_89176_MOESM1_ESM.pdf]

Supplementary Material

GWAS of allometric body-shape indices identifies loci suggesting associations with morphogenesis, organogenesis, adrenal cell renewal and cancer

Christakoudi S., Evangelou E., Riboli E., Tsilidis K.K.

Supplementary Figures

Supplementary Fig. S1 Regression coefficients for independent significant SNPs associated with $ABSI_{UKB}$ or HI_{UKB} 3

Supplementary Fig. S2 Differences between independent significant SNPs associated with $ABSI_{UKB}$ and HI_{UKB} 4

Supplementary Fig. S3 Sex differences between independent significant SNPs associated with allometric body-shape indices 5

Supplementary Fig. S4 Independent significant SNPs previously reported in association with cancer 6

Supplementary Fig. S5 Significant genes identified for allometric body shape indices previously reported in association with cancer 7

Supplementary Fig. S6 Regression coefficient ranking of independent significant SNPs: comparison between pairs of traditional and allometric body-shape indices 8

Supplementary Fig. S7 Differences between independent significant SNPs associated with allometric and traditional body-shape indices in women 9

Supplementary Fig. S8 Differences between independent significant SNPs associated with allometric and traditional body-shape indices in men 10

Supplementary Fig. S9 Independent significant SNPs in women previously reported in association with height 11

Supplementary Fig. S10 Independent significant SNPs in men previously reported in association with height 12

Supplementary Fig. S11 Significant genes identified for allometric body shape indices previously reported in association with height 13

Supplementary Tables

Supplementary Table S1 Demographics and anthropometry of study participants........ 14

Supplementary Table S2 Top lead SNPs associated with allometric body-shape indices and corresponding candidate SNPs reported in the NHGRI-EBI GWAS Catalog 15

Supplementary Table S3 Match of SNPs and genes identified in the current study against the NHGRI-EBI GWAS Catalog 18

Supplementary Table S4 Highest ranked lead genes associated with allometric body-shape indices........ 20

Supplementary Table S5 Pearson's partial correlation coefficients between anthropometric measures and indices 22

Supplementary Table S6 Regression coefficients of models used to calibrate allometric body-shape indices for UK Biobank participants 23

Supplementary Table S7 Genetic variant exclusions defining the genetic relationship matrix 24

Supplementary Table S8 Bayesian mixed-model analysis BOLT-LMM quality control........ 25

Supplementary Table S9 Candidate SNPs identified with FUMA........ 27

Supplementary Table S10 Results from FUMA and MAGMA 29

Supplementary Table S11 GWAS Summary statistics for all candidate SNPs........ 36

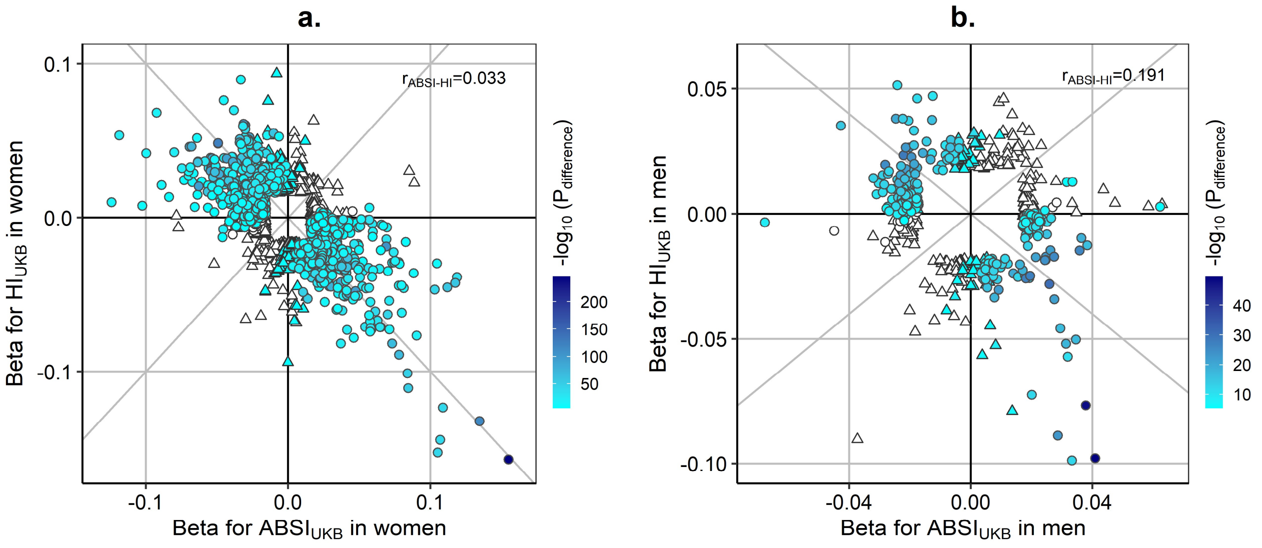

Supplementary Fig. S1 Regression coefficients for independent significant SNPs associated with ABSI$_{UKB}$ or HI$_{UKB}$

(a) – 1,273 independent significant SNPs (with genome-wide significance ($p<5*10^{-8}$) and in approximate linkage equilibrium with each other at $r^2<0.6$) associated with ABSI$_{UKB}$ and 814 associated with HI$_{UKB}$ in women (1,969 unique SNPs, with 1,496 (76%, colour-coded) showing a difference in effect size between ABSI$_{UKB}$ and HI$_{UKB}$ at $P_{difference}<5*10^{-6}$); **(b)** – 266 independent significant SNPs associated with ABSI$_{UKB}$ and 222 associated with HI$_{UKB}$ in men (484 unique SNPs, with 306 (63%, colour-coded) showing significant differences); **ABSI$_{UKB}$** – a body shape index calibrated for UK Biobank participants; **HI$_{UKB}$** – hip index calibrated for UK Biobank participants; **SNPs** – single nucleotide polymorphisms; **WHI$_{UKB}$** – waist-to-hip index calibrated for UK Biobank participants; **$r_{ABSI-HI}$** – Spearman's rank correlation coefficient of the association between regression coefficients for ABSI$_{UKB}$ and HI$_{UKB}$ across all examined genetic variants (used in the test for difference of effect size); **circles** – SNPs with genome-wide significance for WHI$_{UKB}$; **triangles** – SNPs with genome-wide significance only for ABSI$_{UKB}$ or HI$_{UKB}$. Association statistics were derived from BOLT-LMM infinitesimal models.

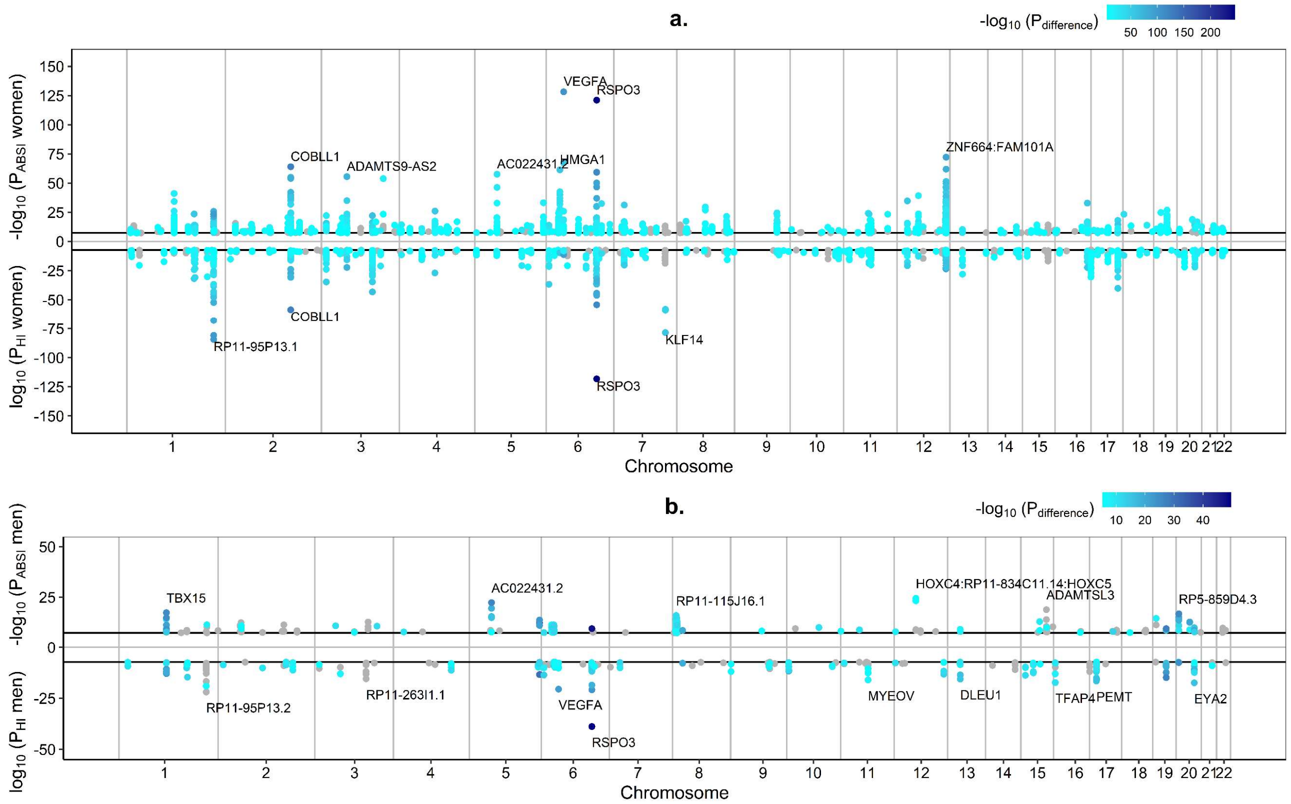

Supplementary Fig. S2 Differences between independent significant SNPs associated with ABSI$_{UKB}$ and HI$_{UKB}$

(a) – 1,273 independent significant SNPs (with genome-wide significance and in approximate linkage equilibrium with each other at r^2<0.6) associated with ABSI$_{UKB}$ and 814 associated with HI$_{UKB}$ in women; **(b)** – 266 independent significant SNPs associated with ABSI$_{UKB}$ and 222 associated with HI$_{UKB}$ in men; **ABSI$_{UKB}$** – a body shape index calibrated for UK Biobank participants; **HI$_{UKB}$** – hip index calibrated for UK Biobank participants; **SNP** – single nucleotide polymorphism; **P$_{WHI / ABSI / HI}$** – p-values for body-shape indices, derived from BOLT-LMM infinitesimal models; **horizontal lines** – genome-wide significance cut-off ($p=5*10^{-8}$); **colour-scale** – independent significant SNPs showing difference in effect size between ABSI$_{UKB}$ and HI$_{UKB}$ at $P_{difference}<5*10^{-6}$.

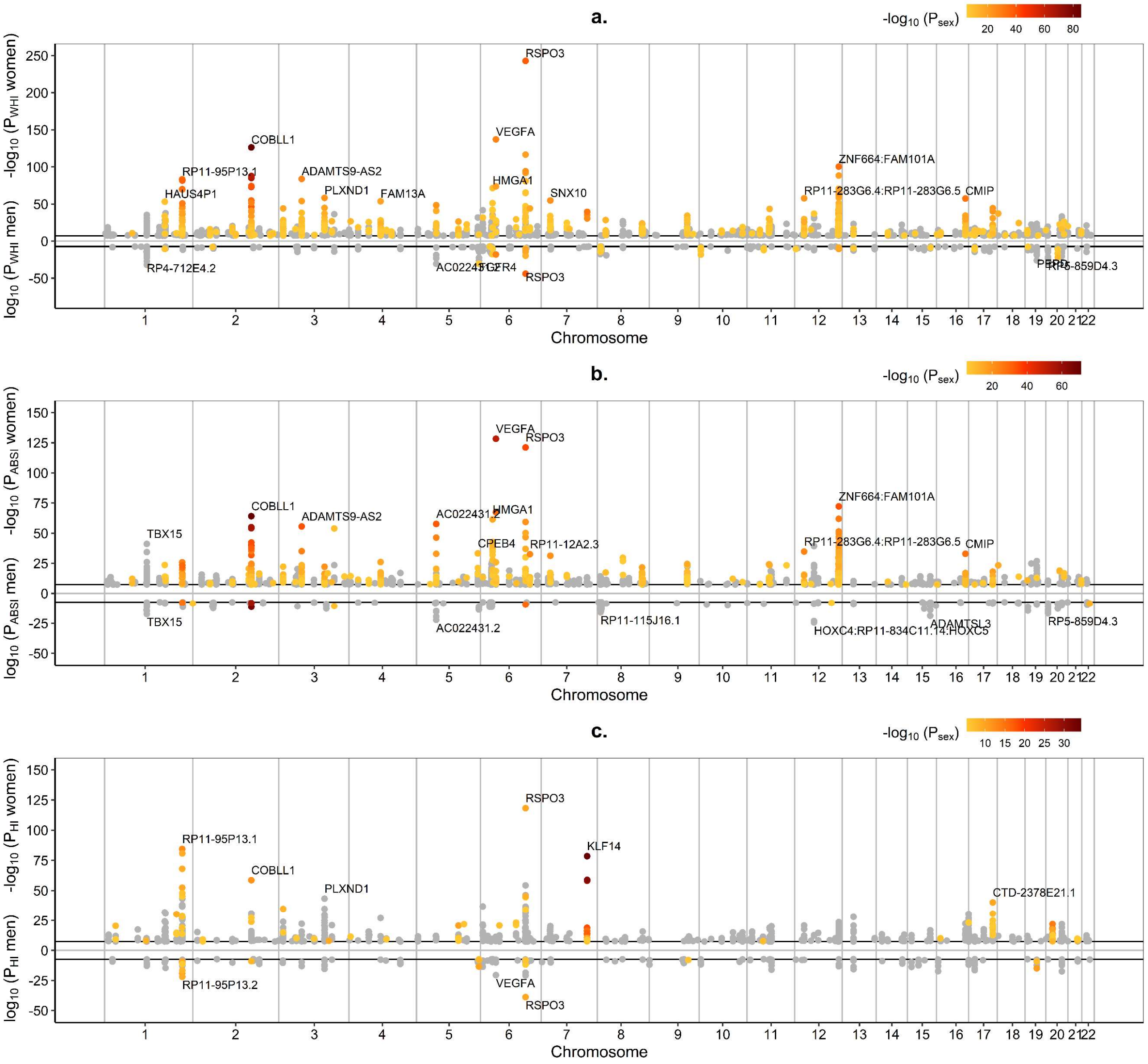

Supplementary Fig. S3 Sex differences between independent significant SNPs associated with allometric body-shape indices

(a) – independent significant single nucleotide polymorphisms (SNPs) identified in this study with FUMA v.1.3.6a (with genome-wide significance and in approximate linkage equilibrium with each other at $r^2<0.6$) for the waist-to-hip index (**WHI**) calibrated for UK Biobank participants (1,988 in women; 617 in men); **(b)** – independent significant SNPs for a body shape index (**ABSI**) calibrated for UK Biobank participants (1,273 in women; 266 in men); **(c)** – independent significant SNPs for hip index (**HI**) calibrated for UK Biobank participants (814 in women; 222 in men); $\mathbf{P_{WHI\,/\,ABSI\,/\,HI}}$ – p-values for body-shape indices derived from BOLT-LMM infinitesimal models; **horizontal lines** – genome-wide significance cut-off ($p=5*10^{-8}$); $\mathbf{P_{sex}}$ – p-values for sex difference in effect size; **Colour scale** – colour-marked were independent significant SNPs with $P_{sex}<5*10^{-6}$.

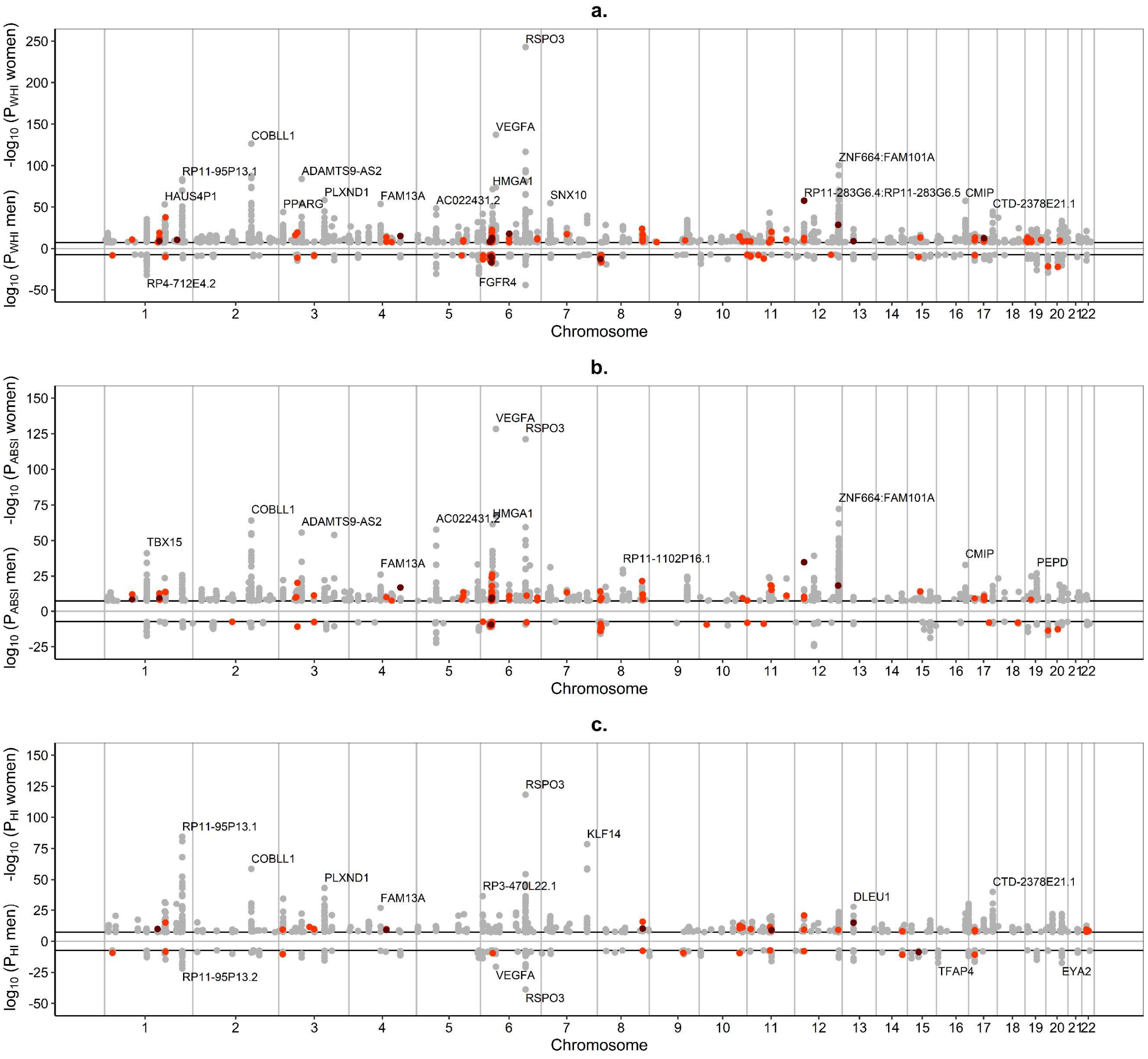

Supplementary Fig. S4 Independent significant SNPs previously reported in association with cancer

(a) – independent significant single nucleotide polymorphisms (SNPs) identified with FUMA v.1.3.6a for waist-to-hip index (**WHI**) calibrated for UK Biobank participants (1,988 in women; 617 in men); **(b)** – independent significant SNPs for a body shape index (**ABSI**) calibrated for UK Biobank participants (1,273 in women; 266 in men); **(c)** – independent significant SNPs for hip index (**HI**) calibrated for UK Biobank participants (814 in women; 222 in men); **$P_{WHI / ABSI / HI}$**– p-values for body-shape indices derived from BOLT -LMM infinitesimal models; **horizontal lines** – genome-wide significance cut-off ($p=5*10^{-8}$). Independent significant SNPs identified in this study with FUMA v.1.3.6a (with genome-wide significance and in approximate linkage equilibrium with each other at $r^2<0.6$) were matched against SNPs reported in the NHGRI-EBI GWAS Catalog [11] in association with cancer (https://www.ebi.ac.uk/gwas/home, accessed on 07/04/2021, catalogue set EFO_0000311).

● (grey) – no reported association with cancer; ● (orange) – reported association with cancer for a candidate SNP in high linkage disequilibrium with the independent significant SNP at $r^2≥0.6$;
● (dark red) – reported association with cancer for the independent significant SNP.

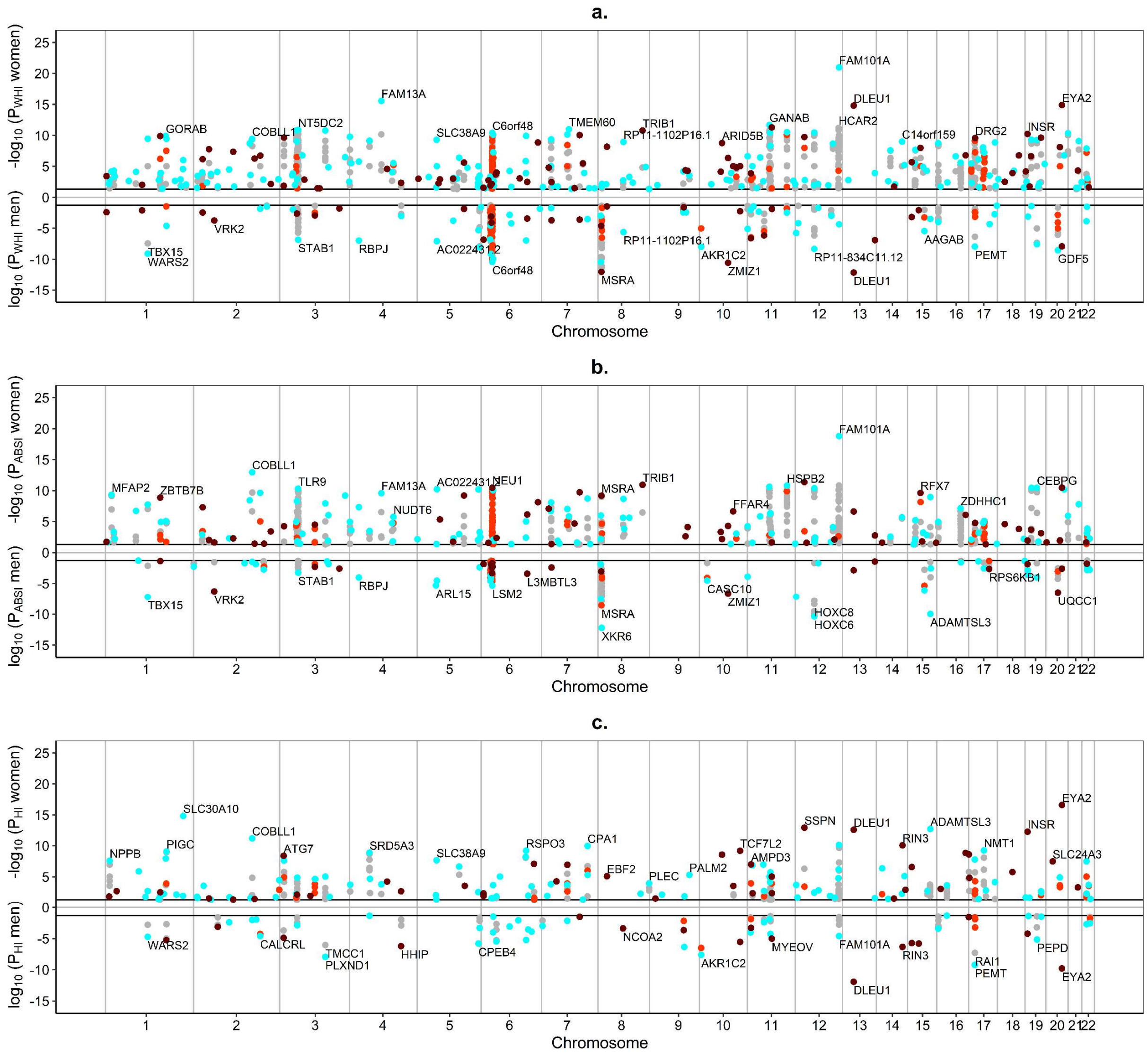

Supplementary Fig. S5 Significant genes identified for allometric body shape indices previously reported in association with cancer

(a) – significant genes for waist-to-hip index (**WHI**) calibrated for UK Biobank participants (759 in women, 303 in men); **(b)** – significant genes for a body shape index (**ABSI**) calibrated for UK Biobank participants (584 in women, 183 in men); **(c)** – significant genes for hip index (**HI**) calibrated for UK Biobank participants (299 for women, 106 for men); $\mathbf{P_{WHI / ABSI / HI}}$ – p-values for body-shape indices derived from MAGMA v1.08 employed in FUMA v1.3.6a, with Bonferroni correction for multiple comparisons of 19,088 genes; **horizontal lines** – nominal significance cut-off (p=0.05) for the adjusted p-values. Significant genes identified in this study within 250 kb of each other were consolidated in a genomic risk region represented by a lead gene (lowest p-value) and were matched against genes reported in the NHGRI-EBI GWAS Catalog [11] in association with cancer (catalogue set EFO_0000311) (https://www.ebi.ac.uk/gwas/home, accessed on 07/04/2021).

● (grey) – significant gene with no reported association with cancer; ● (orange) – significant gene with reported association with cancer; ● (cyan) – lead gene with no reported association with cancer; ● (dark red) – lead gene with reported association with cancer.

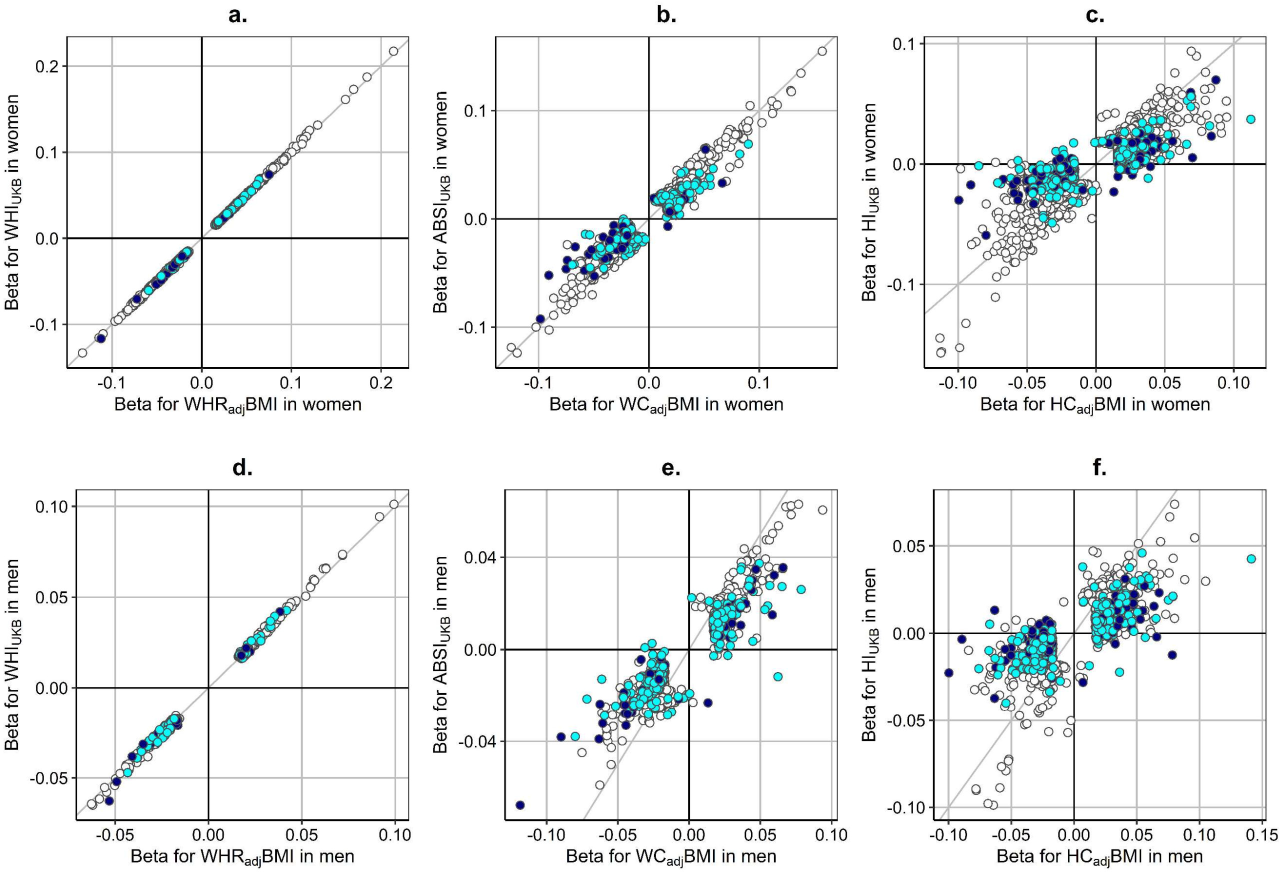

Supplementary Fig. S6 Regression coefficient ranking of independent significant SNPs: comparison between pairs of traditional and allometric body-shape indices

(a) – independent significant SNPs (at r^2<0.6 within 1 Mb window) for WHI$_{UKB}$ (n=1,988) or WHR$_{adj}$BMI (n=1,959) in women; **(b)** – independent significant SNPs for ABSI$_{UKB}$ (n=1,273) or WC$_{adj}$BMI (n=1,586) in women; **(c)** – independent significant SNPs for HI$_{UKB}$ (n=814) or HC$_{adj}$BMI (n=2,008) in women; **(d)** – independent significant SNPs for WHI$_{UKB}$ (n=617) or WHR$_{adj}$BMI (n=571) in men; **(e)** – independent significant SNPs for ABSI$_{UKB}$ (n=266) or WC$_{adj}$BMI (n=842) in men; **(f)** – independent significant SNPs for HI$_{UKB}$ (n=222) or HC$_{adj}$BMI (1,400) in men. Regression coefficients (Beta) were derived from BOLT-LMM infinitesimal models.

ABSI$_{UKB}$ – a body shape index calibrated for UK Biobank participants; **BMI** – body mass index; **HC$_{adj}$BMI** – hip circumference adjusted for BMI; **HI$_{UKB}$** – hip index calibrated for UK Biobank participants; **SNP** – single nucleotide polymorphism; **WC$_{adj}$BMI** – waist circumference adjusted for BMI; **WHR$_{adj}$BMI** – waist-to-hip ratio adjusted for BMI; **WHI$_{UKB}$** – waist-to-hip index calibrated for UK Biobank participants. **Colour scale** – colour-marked were only SNPs reported as associated with height in the NHGRI-EBI GWAS Catalog [11] (https://www.ebi.ac.uk/gwas/home, accessed on 07/04/2021), i.e. included in catalogue sets EFO_0004339 or EFO_0004302; ● (navy) – independent significant SNP reported in association with height; ● (cyan) – independent significant SNP in strong LD (at r^2≥0.6) with a SNP reported in association with height.

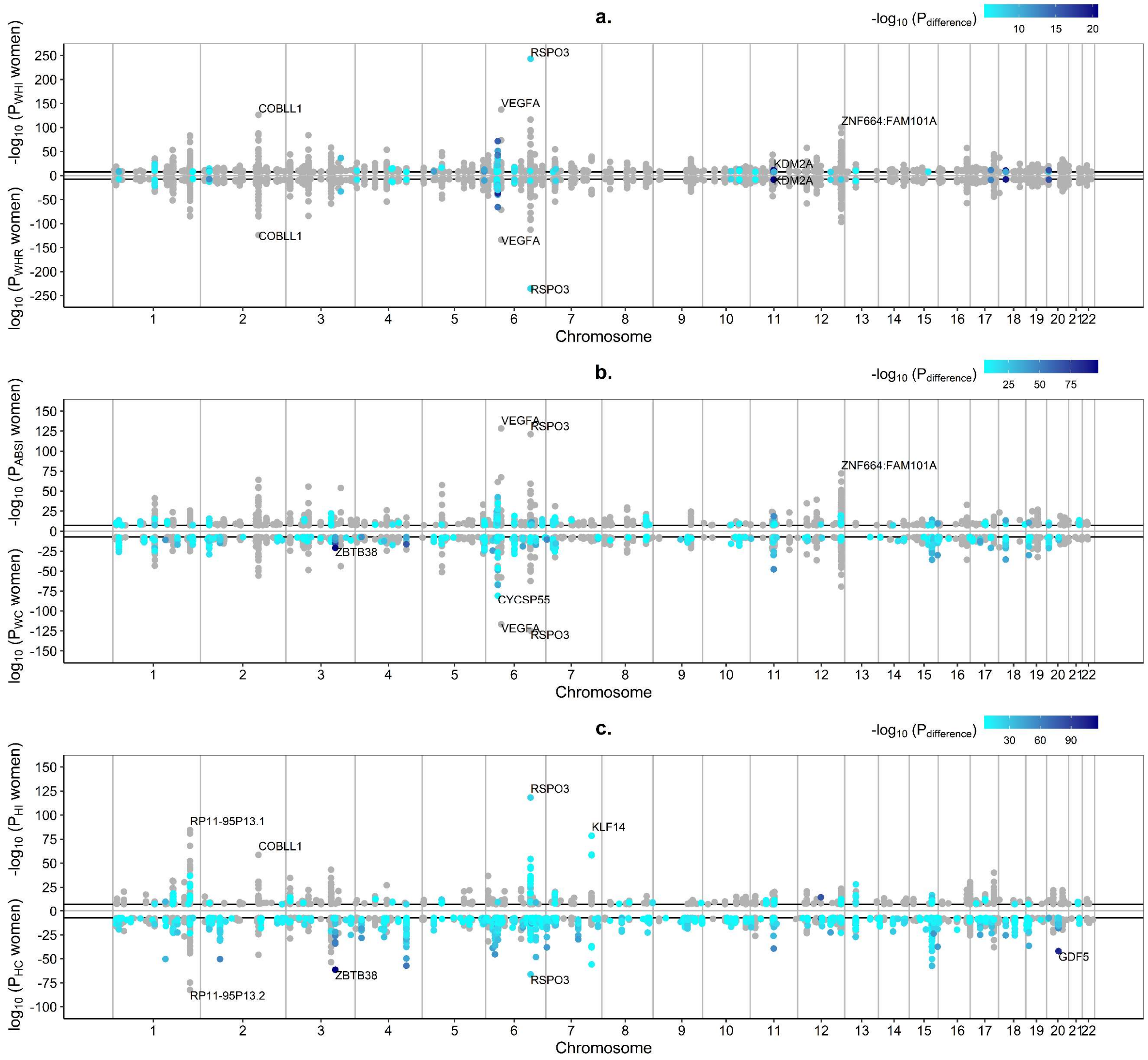

Supplementary Fig. S7 Differences between independent significant SNPs associated with allometric and traditional body-shape indices in women

(a) – independent significant single nucleotide polymorphisms (SNPs) identified in this study with FUMA v.1.3.6a (with genome-wide significance and in approximate linkage equilibrium with each other at $r^2<0.6$) for waist-to-hip index (**WHI**) calibrated for UK Biobank participants (n=1,988) and the waist-to-hip ratio (**WHR**) adjusted for body mass index (BMI) (n=1,959); **(b)** – independent significant SNPs for a body shape index (**ABSI**) calibrated for UK Biobank participants (n=1,273) and waist circumference (**WC**) adjusted for BMI (n=1,586); **(c)** – independent significant SNPs for hip index (**HI**) calibrated for UK Biobank participants (n=814) and hip circumference (**HC**) adjusted for BMI (n=2,008); $\mathbf{P_{WHI / WHR / ABSI / WC / HI / HC}}$ – p-values for body-shape indices derived from BOLT-LMM infinitesimal models; **horizontal lines** – genome-wide significance cut-off ($p=5*10^{-8}$); $\mathbf{P_{difference}}$ – p-values for difference in effect size from the alternative index; **Colour scale** – colour-marked were independent significant SNPs with $P_{difference}<5*10^{-6}$.

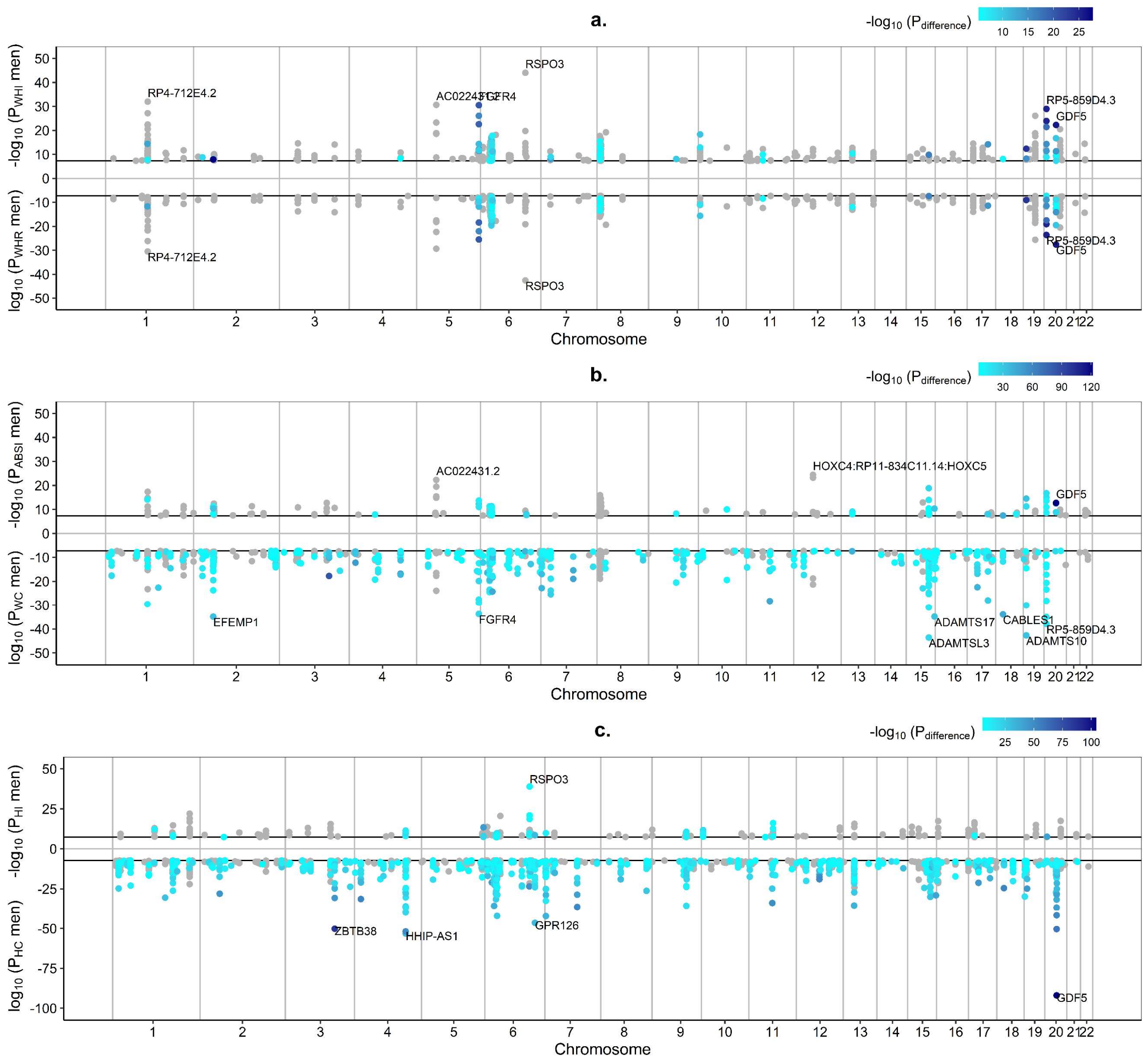

Supplementary Fig. S8 Differences between independent significant SNPs associated with allometric and traditional body-shape indices in men

(a) – independent significant single nucleotide polymorphisms (SNPs) identified in this study with FUMA v.1.3.6a (with genome-wide significance and in approximate linkage equilibrium with each other at $r^2<0.6$) for waist-to-hip index (**WHI**) calibrated for UK Biobank participants (n=617) and for the waist-to-hip ratio (**WHR**) adjusted for body mass index (BMI) (n=571); **(b)** – independent significant SNPs for a body shape index (**ABSI**) calibrated for UK Biobank participants (n=266) and for waist circumference (**WC**) adjusted for BMI (n=842); **(c)** – independent significant SNPs for hip index (**HI**) calibrated for UK Biobank participants (n=222) and for hip circumference (**HC**) adjusted for BMI (n=1400); $\mathbf{P_{WHI\,/\,WHR\,/\,ABSI\,/\,WC\,/\,HI\,/\,HC}}$ – p-values for body-shape indices derived from BOLT-LMM infinitesimal models; **horizontal lines** – genome-wide significance cut-off ($p=5*10^{-8}$); $\mathbf{P_{difference}}$ – p-values for difference in effect size from the alternative index; **Colour scale** – colour-marked were independent significant SNPs with $P_{difference}<5*10^{-6}$.

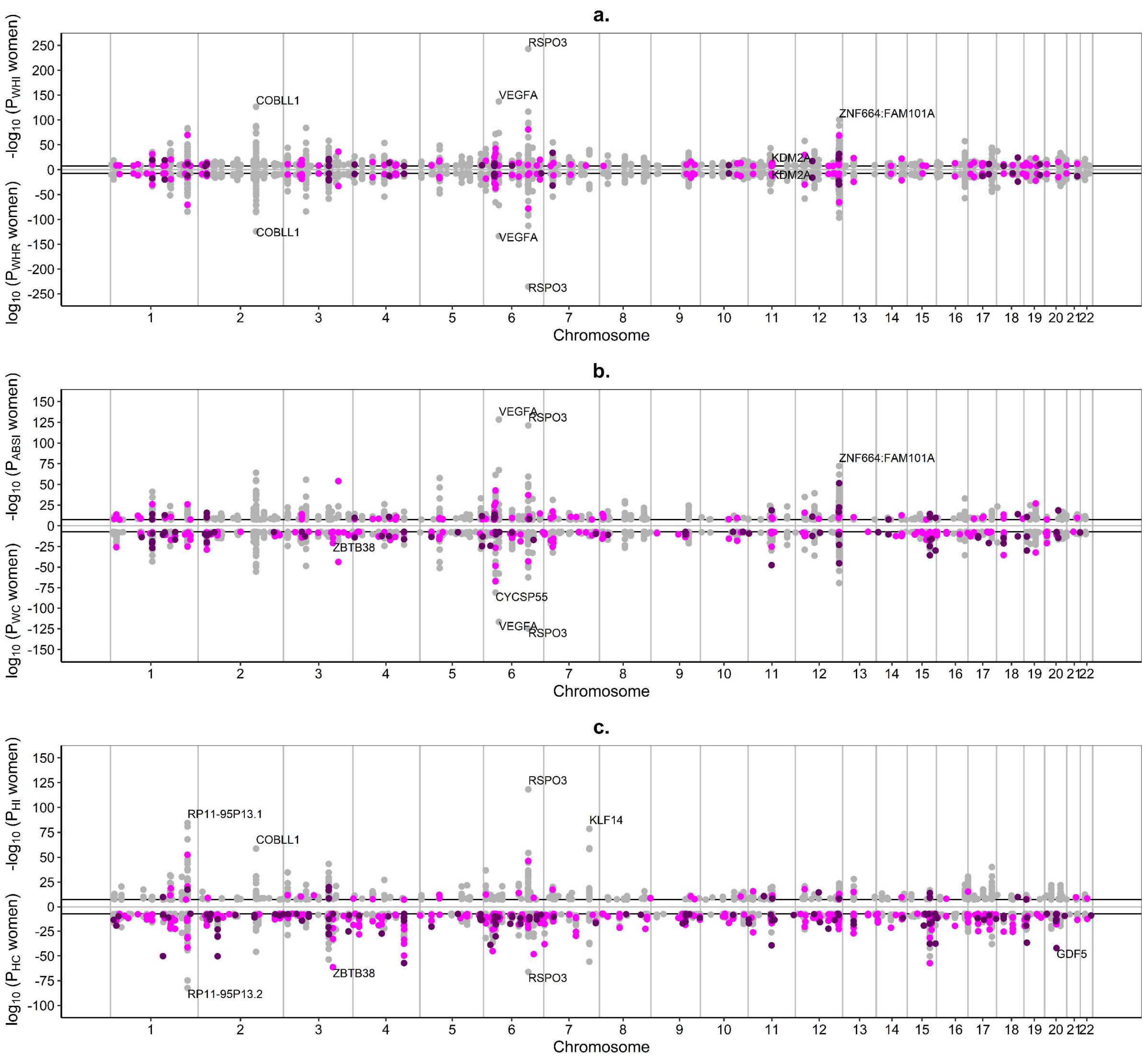

Supplementary Fig. S9 Independent significant SNPs in women previously reported in association with height

(a) – independent significant single nucleotide polymorphisms (SNPs) identified with FUMA v.1.3.6a for waist-to-hip index (**WHI**) calibrated for UK Biobank participants (n=1,988) and for the waist-to-hip ratio (**WHR**) adjusted for body mass index (BMI) (n=1,959); **(b)** – independent significant SNPs for a body shape index (**ABSI**) calibrated for UK Biobank participants (n=1,273) and for waist circumference (**WC**) adjusted for BMI (n=1,586); **(c)** – independent significant SNPs for hip index (**HI**) calibrated for UK Biobank participants (n=814) and for hip circumference (**HC**) adjusted for BMI (n=2,008); $\mathbf{P_{WHI / WHR / ABSI / WC / HI / HC}}$ – p-values for body-shape indices derived from BOLT -LMM infinitesimal models; **horizontal lines** – genome-wide significance cut-off ($p=5*10^{-8}$). Independent significant SNPs identified in this study with FUMA v.1.3.6a (with genome-wide significance and in approximate linkage equilibrium with each other at $r^2<0.6$) were matched against SNPs reported in the NHGRI-EBI GWAS Catalog [11] in association with height (https://www.ebi.ac.uk/gwas/home, accessed on 07/04/2021, catalogue sets EFO_0004339, EFO_0004302). ● (grey) – no reported association with height; ● (light magenta) – reported association with height for a candidate SNP in high linkage disequilibrium ($r^2\geq0.6$); ● (dark magenta) – reported association with height for the independent significant SNP.

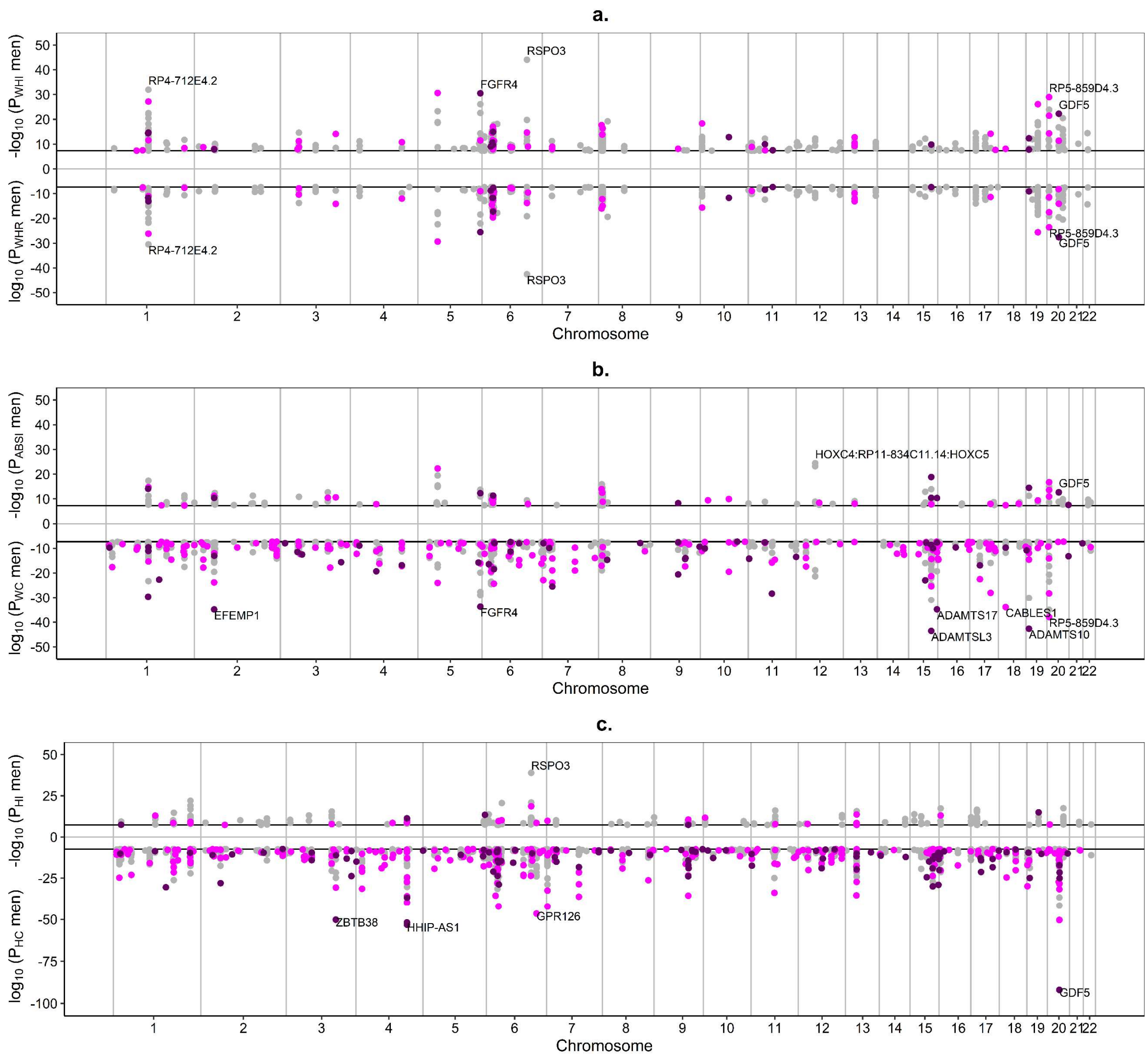

Supplementary Fig. S10 Independent significant SNPs in men previously reported in association with height

(a) – independent significant single nucleotide polymorphisms (SNPs) identified with FUMA v.1.3.6a (with genome-wide significance and in approximate linkage equilibrium with each other at r2<0.6) for waist-to-hip index (**WHI**) calibrated for UK Biobank participants (n=617) and for the waist-to-hip ratio (**WHR**) adjusted for body mass index (BMI) (n=571); (b) – independent significant SNPs for a body shape index (**ABSI**) calibrated for UK Biobank participants (n=266) and for waist circumference (**WC**) adjusted for BMI (n=842); (c) – independent significant SNPs for hip index (**HI**) calibrated for UK Biobank participants (n=222) and for hip circumference (**HC**) adjusted for BMI (n=1400); $\mathbf{P}_{\mathbf{WHI/WHR/ABSI/WC/HI/HC}}$ – p-values for body-shape indices derived from BOLT -LMM infinitesimal models; **horizontal lines** – genome-wide significance cut-off ($p=5*10^{-8}$). Independent significant SNPs identified in this study with FUMA v.1.3.6a (with genome-wide significance and in approximate linkage equilibrium with each other at $r^2<0.6$) were matched against SNPs reported in the NHGRI-EBI GWAS Catalog [11] in association with height (https://www.ebi.ac.uk/gwas/home, accessed on 07/04/2021, catalogue sets EFO_0004339, EFO_0004302).

● (grey) – no reported association with height; ● (light magenta) – reported association with height for a candidate SNP in high linkage disequilibrium ($r^2\geq0.6$); ● (dark magenta) – reported association with height for the independent significant SNP.

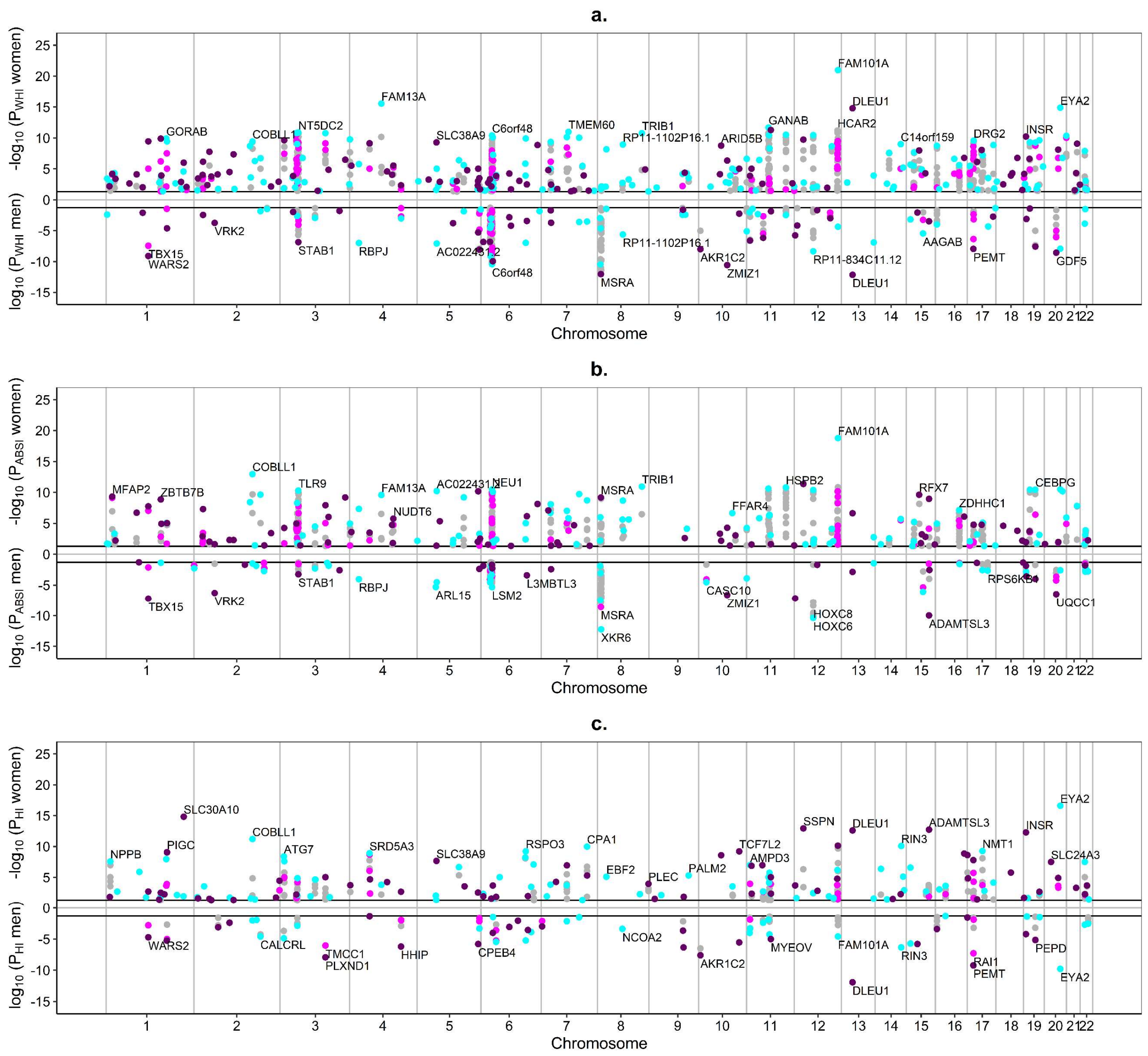

Supplementary Fig. S11 Significant genes identified for allometric body shape indices previously reported in association with height

(a) – significant genes for waist-to-hip index (**WHI**) calibrated for UK Biobank participants (759 in women, 303 in men); (b) – significant genes for a body shape index (**ABSI**) calibrated for UK Biobank participants (584 in women, 183 in men); (c) – significant genes for hip index (**HI**) calibrated for UK Biobank participants (299 for women, 106 for men); $\mathbf{P_{WHI\,/\,ABSI\,/\,HI}}$ – p-values for body-shape indices derived from MAGMA v1.08 employed in FUMA v1.3.6a, with Bonferroni correction for multiple comparisons of 19,088 genes; **horizontal lines** – nominal significance cut-off (p=0.05) for the adjusted p-values. Significant genes identified in this study within 250 kb of each other were consolidated in a genomic risk region represented by a lead gene (lowest p-value) and were matched against genes reported in the NHGRI-EBI GWAS Catalog [11] in association with height (catalogue sets EFO_0004339, EFO_0004302) (https://www.ebi.ac.uk/gwas/home, accessed on 07/04/2021).

● (grey) – significant gene with no reported association with height; ● (light magenta) – significant gene with reported association with height; ● (cyan) – lead gene with no reported association with height; ● (dark magenta) – lead gene with reported association with height.

Supplementary Table S1 Demographics and anthropometry of study participants

**Demographics**	**UK Biobank variable**	**Women**	**Men**
Cohort: n		219,872	186,825
UK BiLEVE Axiom Array: n (%)	22000-0.0	22,240 (10.1)	22,310 (11.9)
Age at baseline: mean (SD)	21003-0.0	56.7 (7.9)	57.1 (8.1)
**Anthropometric measures and traditional indices:** mean (SD)			
Height (cm)	50-0.0	162.6 (6.2)	175.9 (6.7)
Weight (kg)	21002-0.0	71.5 (13.9)	86.2 (14.3)
Body mass index (BMI) (kg/m^2)	21001-0.0	27.0 (5.1)	27.8 (4.2)
Waist circumference (WC) (cm)	48-0.0	84.6 (12.5)	97.1 (11.3)
Hip circumference (HC) (cm)	49-0.0	103.4 (10.3)	103.5 (7.6)
Waist-to-hip ratio (WHR)	1	0.82 (0.07)	0.94 (0.06)
**Allometric indices:** mean (SD)			
A Body Shape Index (ABSI)	2	73.9 (5.0)	79.8 (4.1)
$ABSI_{UKB}$	3	62.1 (4.1)	71.2 (3.7)
Hip Index (HI)	4	64.3 (2.5)	60.2 (2.2)
HI_{UKB}	5	81.4 (3.1)	46.4 (1.6)
Waist-to-hip index (WHI)	6	3.6 (0.3)	4.1 (0.2)
WHI_{UKB}	7	4.5 (0.3)	7.3 (0.4)

1 – calculated with variables 48-0.0 and 49-0.0; **2** – calculated with variables 48-0.0, 50-0.0 and 21002-0.0, using the published formula [ref. 5], which has been derived for participants in the National Health and Nutrition Examination Survey (NHANES); **3** – calculated with variables 48-0.0, 50-0.0 and 21002-0.0, using regression coefficients derived for UK Biobank participants included in the current study; **4** – calculated with variables 49-0.0, 50-0.0 and 21002-0.0, using the published formula [ref. 6], which has been derived for participants in NHANES; **5** – calculated with variables 49-0.0, 50-0.0 and 21002-0.0, using regression coefficients derived for UK Biobank participants included in the current study; **6** – calculated with variables 48-0.0, 49-0.0, 50-0.0 and 21002-0.0, using the regression coefficients from **7** rounded to the nearest proportion in the simplified formula $WHR*[Weight(kg)/Height^2(cm)]^{-1/4}$; **7** – calculated with variables 48-0.0, 49-0.0, 50-0.0 and 21002-0.0, using regression coefficients derived for UK Biobank participants included in the current study; **SD** – standard deviation.

Supplementary Table S2 Top lead SNPs associated with allometric body-shape indices and corresponding candidate SNPs reported in the NHGRI-EBI GWAS Catalog

SNP	CHR	Nearest genes	Beta	P-value	Allele	MAF	Consequences	r^2	CADD
**WHI_{UKB} women**									
rs72959041 [a]	6	*RSPO3*	0.217	$1.5*10^{-243}$	A/G	0.05	Intron, NCT	-	2.9
rs577721086	6	*RSPO3*	0.216	$8.7*10^{-243}$	C/T	0.05	UTR	>0.99	17.6
rs998584 [a]	6	*VEGFA*	0.070	$7.4*10^{-138}$	A/C	0.48	TFB-RR	-	12.4
rs13389219 [a]	2	*COBLL1*	-0.069	$5.2*10^{-127}$	T/C	0.39	NCT, TFB-RR	-	9.1
rs1128249	2	*COBLL1*	-0.069	$1.2*10^{-126}$	T/G	0.39	NCT, TFB-RR	>0.99	15.6
rs7978610 [a]	12	*ZNF664:FAM101A*	-0.064	$3.7*10^{-101}$	C/G	0.33	Intron, NCT, TFB-RR	-	10.5
rs11057401	12	*CCDC92*	-0.060	$5.2*10^{-87}$	A/T	0.31	ExonM, NCT, TFB-RR	0.89	25.3
rs2371767 [a]	3	*ADAMTS9-AS2*	-0.062	$1.5*10^{-84}$	C/G	0.27	NCT	-	5. 5
rs9860730	3	*ADAMTS9-AS2*	-0.058	$3.2*10^{-78}$	G/A	0.29	NCT	0.92	7.1
**WHI_{UKB} men**									
rs577721086 [a]	6	*RSPO3*	0.101	$9.6*10^{-45}$	C/T	0.05	UTR	-	17.6
rs6428789 [a]	1	*RP4-712E4.2*	-0.038	$1.1*10^{-32}$	A/C	0.43	Intergenic	-	8.5
rs465983 [a]	5	*AC022431.2*	0.043	$2.4*10^{-31}$	G/A	0.24	Intron	-	6.9
rs1966265 [a]	5	*FGFR4*	0.042	$3.2*10^{-31}$	A/G	0.25	ExonM, NMDt, NCT, TFB-RR	-	6.2
rs2145271 [a]	20	*RP5-859D4.3*	0.037	$1.1*10^{-29}$	T/A	0.36	Intergenic	-	11.5
**$ABSI_{UKB}$ women**									
rs998584 [a]	6	*VEGFA*	0.069	$5.6*10^{-129}$	A/C	0.48	TFB-RR	-	12.4
rs72959041 [a]	6	*RSPO3*	0.155	$8.3*10^{-122}$	A/G	0.05	Intron, NCT	-	2.9
rs7978610 [a]	12	*ZNF664,FAM101A*	-0.055	$6.7*10^{-73}$	C/G	0.33	Intron, NCT, TFB-RR	-	10.5
rs1128249 [b]	2	*COBLL1*	-0.049	$9.1*10^{-65}$	T/G	0.39	NCT, TFB-RR	-	15.6
rs10195252	2	*COBLL1*	-0.049	$4.6*10^{-63}$	C/T	0.41	NCT	0.94	0.67
rs551980123 [b]	6	*HMGA1*	0.117	$4.2*10^{-62}$	TG/T	0.04	Intron, NCT, TFB-RR	-	11.5
rs9689096	6	*CYCSP55*	0.097	$7.6*10^{-62}$	C/A	0.06	Intergenic	0.71	0.01
rs78114378	6	*CYCSP55*	0.110	$1.7*10^{-61}$	A/G	0.05	Intergenic	0.97	0.2
**$ABSI_{UKB}$ men**									
rs61921797 [b]	12	*HOXC4,[§],HOXC5*	0.033	$3.8*10^{-25}$	A/G	0.37	NCT, Intron, UTR	-	7.4
rs2071449	12	[&]*HOXC4,[§],HOXC5*	0.033	$4.0*10^{-25}$	A/C	0.36	Intron, NCT, TFB-RR	>0.99	2.3
rs754133	12	[&]*HOXC6,HOXC4,[§]*	0.033	$1.0*10^{-24}$	A/G	0.36	Intron, NCT, TFB-RR	0.95	17.1
rs10876528	12	[&]*HOXC6,HOXC4[§]*	0.033	$1.4*10^{-24}$	A/C	0.36	Intron, NCT	0.98	9.3
rs2077177	12	[&]*HOXC6,HOXC4, [§]*	0.03	$6.2*10^{-22}$	C/T	0.40	Intron, NCT, TFB-RR	0.80	6.4
rs157512 [b]	5	*AC022431.2*	0.036	$5.0*10^{-23}$	C/T	0.24	Intron	-	1.9
rs30351	5	*AC022431.1*	0.034	$6.8*10^{-22}$	G/A	0.25	Intergenic	0.90	0.5
rs7162542 [a]	15	*ADAMTSL3*	-0.028	$1.5*10^{-19}$	C/G	0.44	Intron, NCT, TFB-RR	-	2.0
rs4842838	15	*ADAMTSL3*	-0.025	$1.0*10^{-15}$	G/T	0.47	ExonM, NCT	0.72	9.0
rs2030839	15	*ADAMTSL3*	-0.025	$1.0*10^{-15}$	T/C	0.47	Intron, NCT, TFB-RR	0.72	8.5
rs8030379	15	*ADAMTSL3*	-0.025	$1.1*10^{-15}$	G/A	0.47	Intron, NCT	0.72	5.0
rs10802069 [a]	1	*TBX15*	-0.028	$4.5*10^{-18}$	T/C	0.39	Intron	-	16.9
rs2145271 [b]	20	*RP5-859D4.3*	0.027	$1.8*10^{-17}$	T/A	0.37	Intergenic	-	11.5
rs6054427	20	*RP5-859D4.3*	0.027	$2.5*10^{-17}$	G/A	0.38	Intergenic	0.93	18.4
rs979012	20	*RP5-859D4.3*	0.027	$3.2*10^{-17}$	T/C	0.37	Intergenic	>0.99	4.7
rs1884897	20	*RP5-859D4.3*	0.025	$1.2*10^{-14}$	A/G	0.37	TFB-RR	0.88	13.0
rs6107848	20	*CASC20*	0.025	$2.0*10^{-14}$	A/G	0.37	Intergenic	0.84	19.4

(continues)

SNP	CHR	Nearest genes	Beta	P-value	Allele	MAF	Consequences	r2	CADD
**HI_{UKB} women**									
rs72959041 [b]	6	RSPO3	-0.157	$6.3*10^{-119}$	A/G	0.05	Intron, NCT	-	2.9
rs141783576	6	RSPO3	-0.121	$5.5*10^{-100}$	C/G	0.07	UTR, TFB-RR	0.66	15.6
rs2605098 [a]	1	RP11-95P13.1	0.060	$3.9*10^{-85}$	A/G	0.34	Intergenic	-	0.1
rs748273	1	RP11-95P13.1	0.059	3.10E-78	A/G	0.31	Intergenic	0.83	8.3
rs4731702 [b]	7	KLF14	0.055	$3.4*10^{-79}$	T/C	0.49	Intergenic	-	0.1
rs13241538	7	KLF14	0.055	$5.8*10^{-78}$	C/G	0.49	Intergenic	>0.99	0.3
rs17789506	7	KLF14	0.055	$1.2*10^{-77}$	A/G	0.49	Intergenic	>0.99	3.1
rs13389219 [b]	2	COBLL1	0.049	$2.7*10^{-59}$	T/C	0.39	NCT, TFB-RR	-	9.1
rs10195252	2	COBLL1	0.047	$8.4*10^{-57}$	C/T	0.41	NCT	0.94	0.7
rs9840468 [b]	3	PLXND1	0.051	$5.9*10^{-44}$	G/A	0.20	NCT, TFB-RR	-	13.3
rs10804591	3	PLXND1	0.048	$2.0*10^{-41}$	C/A	0.22	TFB-RR	0.88	9.8
**HI_{UKB} men**									
rs72959041 [b]	6	RSPO3	-0.098	$1.3*10^{-39}$	A/G	0.05	Intron, NCT	-	2.9
rs141783576	6	RSPO3	-0.077	$9.8*10^{-35}$	C/G	0.07	UTR, TFB-RR	0.66	15.6
rs1415287 [b]	1	RP11-95P13.2	0.034	$1.1*10^{-22}$	T/C	0.30	TFB-RR	-	1.7
rs2820443	1	RP11-95P13.2	0.034	$1.9*10^{-22}$	C/T	0.30	TFB-RR	>0.99	13.0
rs998584 [c]	6	VEGFA	-0.030	$2.9*10^{-21}$	A/C	0.48	TFB-RR		12.4
rs6066114 [c]	20	EYA2	-0.029	$3.5*10^{-18}$	C/T	0.35	Intron, NMDt		3.2
rs113733630 [d]	16	TFAP4	0.032	$4.7*10^{-18}$	#/A	0.24	Intergenic	-	0.7
rs3747579	16	CORO7-PAM16:CORO7	-0.017	$1.3*10^{-6}$	C/T	0.28	ExonM, NMDt, UTR, Intron, NCT	-	27.8

Shown are the five highest-ranked top lead SNP per index and sex identified with FUMA v1.3.6a (each corresponding to an independent genomic risk locus), in ascending order of their p-value.

[a] – top lead SNP previously reported in association with the corresponding traditional body-shape index (see matching algorithm below, additional reported SNPs from the same genetic risk locus are shown in the grey rows only if their CADD is larger); [b] – top lead SNP in strong linkage disequilibrium (at $r^2 \geq 0.6$) with a previously reported candidate SNP (the grey rows show the previously reported SNPs from the LD block and when there is more than one, only those with higher CADD); [c] – novel top lead SNP, with no previously reported candidate SNPs included in the corresponding genetic risk locus; [d] – top lead SNP with a previously reported candidate SNP included in the corresponding genetic risk locus, but not in the same LD block (shown in the grey row); [#] – AGTTT; [&] – *RP11-834C11.12;* [§] – *RP11-834C11.14*; **$ABSI_{UKB}$** – a body shape index calibrated for UK Biobank participants; **Allele** – allele variants (minor allele first); **Beta** – regression coefficients reflecting the phenotype of the minor allele for the corresponding index, derived from BOLT-LMM infinitesimal models; **CADD** – Combined Annotation Dependent Depletion (deleteriousness) score; **ExonM** – exon missense; **Consequences** – correspond to annotation according to Ensembl Variance Effect Predictor (VEP) v.90 **HI_{UKB}** – hip index calibrated for UK Biobank participants; **MAF** – minor allele frequency; **NCT** – non-coding transcript; **NMDt** – nonsense-mediated mRNA decay (NMD) target; **P-value** – derived from BOLT-LMM infinitesimal models; **r^2** – maximum value of linkage disequilibrium with the top lead variant from [a/b/c/d] mapped to the same genetic region; **SNP** – single nucleotide polymorphism; **TFB-RR** – transcription factor

(TF) binding or regulatory region; **UTR** – untranslated region; **WHI_{UKB}** – waist-to-hip index calibrated for UK Biobank participants.

SNPs identified in the current study for allometric body-shape indices were matched against SNPs reported in the NHGRI-EBI GWAS Catalog [ref. 11] (https://www.ebi.ac.uk/gwas/home, accessed on 07/04/2021) in association with the corresponding traditional body-shape index (with or without adjustment for body mass index), i.e. the waist-to-hip ratio for WHI_{UKB} (catalogue sets EFO_0004343, EFO_0007788, EFO_0004302), waist circumference for $ABSI_{UKB}$ (EFO_0004342, EFO_0007789, EFO_0004302), or hip circumference for HI_{UKB} (EFO_0005093, EFO_0008039, EFO_0004302). Variants in locus *RP4-712E4.2* have previously been mapped to *AL*139420.2 and *WARS2*, in locus *RP5-859D4.3* to *LINC01713* and *CASC20,* in locus *RP11-95P13.1* to *AL356364.1* and *LYPLAL1-AS1,* in locus *RP11-95P13.2* to *SLC30A10, AL356364.1* and *ZC3H11B* and in locus *AC022431.2* to *C5orf67*.

Supplementary Table S3 Match of SNPs and genes identified in the current study against the NHGRI-EBI GWAS Catalog

Catalogue set	WHI_{UKB}	$WHR_{adj}BMI$	$ABSI_{UKB}$	$WC_{adj}BMI$	HI_{UKB}	$HC_{adj}BMI$
				**Women**		
**Independent significant SNPs** [a]						
Total number	1,988	1,959	1,273	1,586	814	2,008
$WHR_{adj}BMI$ or WHR [1]	729 (37)	718 (37)	475 (37) ***	472 (30) ***	236 (29) ***	279 (14) ***
$WC_{adj}BMI$ or WC [2]	292 (15)	270 (14)	278 (22) ***	478 (30) ***	87 (11)	253 (13)
$HC_{adj}BMI$ or HC [3]	41 (2.1)	41 (2.1)	33 (2.6)	57 (3.6)	40 (4.9)	121 (6.0)
VAT or fat distribution [4]	97 (4.9)	95 (4.8)	102 (8.0)	135 (8.5)	52 (6.4) *	196 (9.8) *
BMI, weight or adipose tissue [5]	133 (6.7)	128 (6.5)	115 (9.0)	167 (11)	57 (7.0) *	208 (10) *
Height [6]	132 (6.6)	127 (6.5)	103 (8.1) ***	227 (14) ***	52 (6.4) ***	440 (22) ***
**Significant genes** [b]						
Total number	759	748	584	707	299	923
$WHR_{adj}BMI$ or WHR [1]	342 (45)	337 (45)	251 (43)	270 (38)	126 (42) ***	219 (24) ***
$WC_{adj}BMI$ or WC [2]	190 (25)	177 (24)	172 (29) *	262 (37) *	68 (23)	195 (21)
$HC_{adj}BMI$ or HC [3]	50 (6.6)	49 (6.6)	35 (6.0)	63 (8.9)	33 (11)	90 (9.8)
VAT or fat distribution [4]	103 (14)	101 (14)	90 (15)	129 (18)	57 (19)	170 (18)
BMI, weight or adipose tissue [5]	172 (23)	169 (23)	136 (23)	187 (26)	89 (30)	231 (25)
Height [6]	196 (26)	188 (25)	141 (24) *	228 (32) *	96 (32) *	373 (40) *
				**Men**		
**Independent significant SNPs** [a]						
Total number	617	571	266	842	222	1,400
$WHR_{adj}BMI$ or WHR [1]	173 (28)	161 (28)	64 (24) ***	106 (13) ***	103 (46) ***	157 (11) ***
$WC_{adj}BMI$ or WC [2]	104 (17)	88 (15)	65 (24) *	283 (34) *	45 (20)	212 (15)
$HC_{adj}BMI$ or HC [3]	20 (3.2)	18 (3.2)	19 (7.1)	61 (7.2)	22 (9.9)	110 (7.9)
VAT or fat distribution [4]	76 (12)	69 (12)	62 (23) **	112 (13) **	28 (13)	181 (13)
BMI, weight or adipose tissue [5]	66 (11)	60 (11)	44 (17)	120 (14)	37 (17) *	147 (10) *
Height [6]	64 (10)	55 (9.6)	40 (15) ***	235 (28) ***	30 (14) ***	373 (27) ***
**Significant genes** [b]						
Total number	303	312	183	421	106	724
$WHR_{adj}BMI$ or WHR [1]	106 (35)	104 (33)	51 (28)	102 (24)	54 (51) ***	146 (20) ***
$WC_{adj}BMI$ or WC [2]	72 (24)	71 (23)	45 (25) *	162 (38) *	32 (30) *	150 (21) *
$HC_{adj}BMI$ or HC [3]	18 (5.9)	18 (5.8)	12 (6.6) *	51 (12) *	22 (21) *	86 (12) *
VAT or fat distribution [4]	48 (16)	51 (16)	41 (22)	84 (20)	21 (20)	152 (21)
BMI, weight or adipose tissue [5]	61 (20)	61 (20)	47 (26)	122 (29)	37 (35) *	175 (24) *
Height [6]	76 (25)	80 (26)	47 (26) ***	182 (43) ***	38 (36)	297 (41)

$ABSI_{UKB}$ – a body shape index calibrated for UK Biobank participants; **BMI** – body mass index; **$HC_{adj}BMI$** – hip circumference adjusted for body mass index; **HI_{UKB}** – hip index calibrated for UK Biobank participants; **SNP** – single nucleotide polymorphism; **VAT** – visceral adipose tissue; **$WC_{adj}BMI$** – waist circumference adjusted for BMI; **$WHR_{adj}BMI$** – waist-to-hip ratio adjusted for BMI; **WHI_{UKB}** – waist-to-hip index calibrated for UK Biobank participants. Association statistics were derived from BOLT-LMM infinitesimal models.

[a] – defined according to FUMA v1.3.6a, i.e. with genome-wide significance at $p<5*10^{-8}$ in the current study and in approximate linkage equilibrium with each other at $r^2<0.6$; a match was counted if any candidate SNP from the corresponding LD block (with p<0.05, within 1 Mb and in LD with the independent significant SNP at $r^2\geq0.6$) was reported in the examined catalogue set.

[b] – identified with MAGMA v1.08 employed in FUMA v1.3.6a at significance $P_{adj}<0.05$, incorporating Bonferroni correction for multiple comparisons for 19,088 protein-coding genes.

Independent significant SNPs and significant genes identified in the current study were matched against SNPs and genes reported in the NHGRI-EBI GWAS Catalog [ref. 11] (accessed on 07/04/2021, https://www.ebi.ac.uk/gwas/home) in association with anthropometric measures and indices.

Catalogue sets were as follows (**n** – number of variants; **s** – number of studies in the set):

[1] EFO_0004343 waist-to-hip ratio (n=4,740, s=87); EFO_0007788 BMI-adjusted waist-to-hip ratio (n=3,177, s=46);

[2] EFO_0004342 waist circumference (n=3,211, s=43); EFO_0007789 BMI-adjusted waist circumference (n=2744, s=15);

[3] EFO_0005093 hip circumference (HC) (n=690, s=11); EFO_0008039 BMI-adjusted hip circumference (n=374, s=2);

[4] EFO_0004341 body fat distribution (n=526, s=6); EFO_0004765 visceral adipose tissue measurement (n=360, s=26); EFO_0004767 visceral/subcutaneous adipose tissue ratio (n=54, s=12); EFO_0005106 body composition measurement (n=897, s=23);

[5] EFO_0004338 body weight (n=848, s=44); EFO_0004340 body mass index (BMI) (n=7,876, s=194); EFO_0004764 adipose tissue measurement (n=532, s=66); EFO_0004766 subcutaneous adipose tissue measurement (n=65, s=14); EFO_0005409 fat body mass (n=231, s=14); EFO_0007800 body fat percentage (n=298, s=16);

[6] EFO_0004339 body height (Height) (n=5,514, s=56);

In addition, entries from set EFO_0004302 anthropometric measurement (n=26,484, s=585) were allocated to the corresponding index.

Supplementary Table S4 Highest ranked lead genes associated with allometric body-shape indices

Lead gene	CHR	Start BP	Stop BP	kb	nGenes	nSNPs	P_{adj}	Catalog	Height	Cancer
**WHI_{UKB} women**										
FAM101A	12	122456328	124801570	2345	40	1148	$1.1*10^{-21}$	Proxy	No	No
ZNF664						90	$1.0*10^{-10}$	Yes	No	No
FAM13A	4	89441199	90037050	596	4	1391	$2.8*10^{-16}$	Yes	No	No
EYA2	20	45522263	45818492	296	1	954	$1.3*10^{-15}$	Yes	No	Yes
DLEU1	13	50655307	51298372	643	1	1807	$1.6*10^{-15}$	Yes	Yes	Yes
GANAB	11	62062754	62522660	460	23	29	$2.1*10^{-12}$	Yes	No	No
**WHI_{UKB} men**										
DLEU1	13	50655307	51298372	643	1	1807	$7.2*10^{-13}$	Yes	Yes	Yes
MSRA	8	9910778	11874043	1963	22	1662	$9.5*10^{-13}$	Proxy	Yes	Yes
PINX1						457	$7.7*10^{-5}$	Yes	No	No
ZMIZ1	10	80827792	81077276	249	1	817	$2.7*10^{-11}$	Yes	Yes	Yes
ERI1	8	8639864	9026646	387	4	501	$3.5*10^{-11}$	Novel	No	No
C6orf48	6	29908037	32732311	2824	69	27	$3.7*10^{-11}$	Proxy	No	No
EHMT2						62	$2.3*10^{-10}$	Yes	No	No
**$ABSI_{UKB}$ women**										
FAM101A	12	122456328	124801570	2345	33	1148	$1.6*10^{-19}$	Proxy	No	No
DNAH10OS						21	$1.5*10^{-11}$	Yes	No	No
COBLL1	2	165348322	165813035	465	3	619	$1.1*10^{-13}$	Yes	No	No
SSPN	12	26273924	26987131	713	2	622	$4.3*10^{-12}$	Yes	Yes	Yes
TRIB1	8	126102921	126451647	349	2	17	$1.2*10^{-11}$	Yes	No	Yes
HSPB2	11	111596632	112065528	469	15	10	$1.6*10^{-11}$	Proxy	No	No
PPP2R1B						40	$1.3*10^{-10}$	Yes	No	Yes
**$ABSI_{UKB}$ men**										
XKR6	8	9910778	11874043	1963	19	1144	$5.9*10^{-13}$	Novel	No	No
HOXC6	12	54377849	54450813	73	7	80	$4.1*10^{-11}$	Yes	No	No
ADAMTSL3	15	84114980	84915120	800	3	1120	$1.0*10^{-10}$	Yes	Yes	No
ERI1	8	8639864	9026646	387	4	501	$3.2*10^{-8}$	Novel	No	No
TBX15	1	119424669	119684294	260	2	310	$6.0*10^{-8}$	Yes	Yes	No
**HI_{UKB} women**										
EYA2	20	45522263	45818492	296	1	954	$2.6*10^{-17}$	Novel	No	Yes
SLC30A10	1	219857769	220132989	275	1	1006	$1.5*10^{-15}$	Novel	Yes	No
SSPN	12	26271959	26987131	715	3	622	$1.2*10^{-13}$	Novel	Yes	Yes
ADAMTSL3	15	83423114	84709594	1286	11	1120	$1.9*10^{-13}$	Yes	Yes	No
DLEU1	13	50655307	51298372	643	1	1807	$2.6*10^{-13}$	Yes	Yes	Yes
**HI_{UKB} men**										
DLEU1	13	50655307	51298372	643	1	1807	$1.2*10^{-12}$	Yes	Yes	Yes
EYA2	20	45522263	45818492	296	1	954	$1.6*10^{-10}$	Novel	No	Yes
PEMT	17	17407877	18084116	676	5	243	$5.9*10^{-10}$	Novel	Yes	No
PLXND1	3	129273018	129613419	340	2	185	$1.2*10^{-8}$	Yes	Yes	No
AKR1C2	10	4933796	5061223	127	2	190	$2.5*10^{-8}$	Novel	Yes	No

Shown are the five highest-ranked lead genes per index and sex in ascending order of their p-value. Significant SNPs were identified with MAGMA v1.08 employed in FUMA v1.3.6a and those

within 250 kb of each other were consolidated in genomic risk regions, represented by a lead gene (lowest p-value).

CHR – chromosome; **Start BP** – starting position of the earliest gene included in the genomic risk region; **Stop BP** – stopping position of the latest gene included in the genomic risk region; **kb** – length in kb pairs of the genomic risk region; **nGenes** – number of genes included in the genomic risk region; **nSNPs** – number of single nucleotide polymorphisms included in the lead gene; $\mathbf{P_{adj}}$ – gene-level association p-value from MAGMA, with Bonferroni correction for multiple comparisons of 19,088 genes; **Catalog** – match of any gene in the genomic risk region with a gene previously reported in association with the corresponding traditional body-shape index; **Height** – match of the gene with a gene previously reported in association with height; **Cancer** – match of the gene with a gene previously reported in association with cancer; **Yes** – previously reported lead gene (or previously reported gene, for match with cancer or height); **Proxy** – previously reported gene included in the genomic risk locus but not the lead gene (the grey rows show the previously reported gene with lowest p-value in the corresponding genomic risk region); **No** – not reported.

Significant genes were matched against genes reported in the NHGRI-EBI GWAS Catalog [ref. 11] (https://www.ebi.ac.uk/gwas/home, accessed on 07/04/2021) in association with the corresponding traditional body-shape index (with or without adjustment for body mass index) (column Catalog), i.e. the waist-to-hip ratio for WHI_{UKB} (catalogue sets EFO_0004343, EFO_0007788, EFO_0004302), waist circumference for $ABSI_{UKB}$ (EFO_0004342, EFO_0007789, EFO_0004302), or hip circumference for HI_{UKB} (EFO_0005093, EFO_0008039, EFO_0004302), or in association with height (for column Height) (EFO_0004339, EFO_0004302) or cancer (for column Cancer) (EFO_0000311).

Supplementary Table S5 Pearson's partial correlation coefficients between anthropometric measures and indices

WOMEN / MEN	Height	Weight	BMI	WHR	WHR$_{adj}$BMI	WHI	WHI$_{UKB}$	WC	WC$_{adj}$BMI	ABSI	ABSI$_{UKB}$	HC	HC$_{adj}$BMI	HI	HI$_{UKB}$
**Height**		0.30	-0.13	-0.07	-0.01	0.00	0.03	0.05	0.32	0.05	0.04	0.15	0.51	-0.04	0.01
**Weight**	0.42		0.88	0.43	0.01	-0.06	0.01	0.85	0.14	0.12	0.00	0.90	0.21	0.01	-0.01
**BMI**	-0.07	0.91		0.48	0.02	-0.06	0.00	0.86	0.01	0.11	-0.01	0.87	0.00	0.02	-0.02
**WHR**	-0.05	0.55	0.63		0.88	0.84	0.87	0.81	0.55	0.64	0.60	0.34	-0.28	-0.51	-0.34
**WHR$_{adj}$BMI**	-0.01	0.02	0.04	0.79		0.99	1.00	0.36	0.70	0.76	0.76	-0.18	-0.36	-0.42	-0.43
**WHI**	-0.02	0.10	0.12	0.84	0.99		1.00	0.44	0.70	0.77	0.77	-0.11	-0.35	-0.43	-0.42
**WHI$_{UKB}$**	0.04	0.02	0.01	0.78	1.00	0.99		0.35	0.72	0.77	0.78	-0.18	-0.32	-0.40	-0.42
**WC**	0.15	0.86	0.87	0.76	0.40	0.34	0.39		0.49	0.57	0.46	0.81	0.12	0.06	0.03
**WC$_{adj}$BMI**	0.42	0.21	0.01	0.71	0.80	0.80	0.81	0.49		0.95	0.95	0.12	0.22	0.04	0.05
**ABSI**	0.08	0.10	0.07	0.79	0.84	0.83	0.84	0.52	0.93		0.99	0.15	0.09	0.07	0.07
**ABSI$_{UKB}$**	0.05	0.02	-0.01	0.74	0.84	0.85	0.85	0.44	0.92	1.00		0.03	0.08	0.07	0.07
**HC**	0.29	0.87	0.79	0.25	-0.17	-0.24	-0.18	0.82	0.26	0.20	0.13		0.48	0.42	0.40
**HC$_{adj}$BMI**	0.57	0.25	-0.02	-0.34	-0.39	-0.39	-0.37	0.19	0.41	0.24	0.22	0.58		0.83	0.85
**HI**	-0.01	-0.30	-0.33	-0.38	-0.47	-0.46	-0.46	-0.19	0.18	0.20	0.23	0.19	0.76		1.00
**HI$_{UKB}$**	0.01	-0.01	-0.03	-0.40	-0.47	-0.46	-0.46	0.09	0.21	0.24	0.24	0.47	0.82	0.95	

Men – bottom-left half of panel; **Women** – top-right half of panel; **ABSI** – a body shape index; **BMI** – body mass index; **HC** – hip circumference; **HI** – hip index; **WC** – waist circumference; **WHI** – waist-to-hip index; **WHR** – waist-to-hip ratio; **…$_{adj}$BMI** – … adjusted for BMI;

Note: ABSI and HI were calculated using published regression coefficients [refs. 5, 6]; Indices marked with "**…$_{UKB}$**" were calculated using regression coefficients derived from UK Biobank data; WHI was calculated rounding the regression coefficients obtained from UK Biobanks data to whole numbers and simple fractions with the formula: WHI = WHR * [Weight (kg) / Height^2 (cm)]$^{-1/4}$. All anthropometric measures correspond to the baseline visit. Pearson's partial correlation coefficients were derived with adjustment for age at the baseline visit.

Supplementary Table S6 Regression coefficients of models used to calibrate allometric body-shape indices for UK Biobank participants

	Women				Men			
	Estimate	Standard Error	t-value	R^2	Estimate	Standard Error	t-value	R^2
**Waist circumference** (cm) **[48-0.0]** for **ABSI$_{UKB}$***								
Intercept	-2.78104	0.00340	-819	0.784	-2.64309	0.00335	-790	0.795
Weight (kg)	0.71354	0.00080	892		0.69306	0.00082	844	
Height (m)	-0.88703	0.00386	-230		-0.83902	0.00343	-245	
**Hip circumference** (cm) **[49-0.0]** for **HI$_{UKB}$**								
Intercept	4.39844	0.01111	396	0.836	3.83776	0.01117	343	0.759
Weight (kg)	0.49002	0.00047	1048		0.40345	0.00055	727	
Height (cm)	-0.36311	0.00226	-161		-0.19217	0.00232	-83	
**Waist-to-hip ratio [48-0.0 / 49-0.0]** for **WHI$_{UKB}$**								
Intercept	1.51062	0.02123	71	0.229	1.98816	0.01749	114	0.377
Weight (kg)	0.22352	0.00089	250		0.28961	0.00087	334	
Height (cm)	-0.52393	0.00432	-121		-0.64685	0.00363	-178	

ABSI – a body shape index; **HI** – hip index; **WHI** – waist-to-hip index; **Weight** – at baseline; **Height** – at baseline; Measures were used in the units specified in brackets; **t-value** – Wald test statistics (+/-1.96 corresponds to significance level p=0.05); **R^2** – proportion explained variability; * – the values for ABSI were additionally multiplied by 1000, in order to obtain numbers in the magnitude of waist circumference; **[…]** – code corresponding to the UK Biobank variable(s) used to generate the index. Each of the log-transformed body-shape indices was regressed on log-transformed weight and log-transformed height.

Supplementary Table S7 Genetic variant exclusions defining the genetic relationship matrix

		WOMEN (n=219,872)					MEN (n=186,825)				
	Loaded	GT	Removed variants			Used	GT	Removed variants			Used
CHR	variants	rate	--geno	--hwe	--maf	variants	rate	--geno	--hwe	--maf	variants
1	63,487	0.9742	8,500	2,653	26,837	25,497	0.9737	8,494	2,464	26,957	25,572
2	61,966	0.9675	9,188	2,536	25,011	25,231	0.9672	91,68	2,327	25,131	25,340
3	52,300	0.9691	7,603	1,964	21,344	21,389	0.9687	7,580	1,804	21,440	21,476
4	47,443	0.9731	6,742	2,034	18,720	19,947	0.9728	6,709	1,858	18,847	20,029
5	46,314	0.9585	7,100	1,992	18,170	19,052	0.9584	7,085	1,815	18,302	19,112
6	53,695	0.9752	7,847	2,305	20,089	23,454	0.9745	7,826	2,142	20,173	23,554
7	42,722	0.9719	6,038	1,870	17,059	17,755	0.9716	6,048	1,732	17,137	17,805
8	38,591	0.9739	5,157	1,699	15,377	16,358	0.9737	5,144	1,556	15,495	16,396
9	34,310	0.9733	4,890	1,525	13,465	14,430	0.9728	4,890	1,384	13,544	14,492
10	38,308	0.9701	5,579	1,661	15,147	15,921	0.9697	5,559	1,546	15,208	15,995
11	40,824	0.9671	6,029	1,656	17,108	16,031	0.9667	6,027	1,508	17,189	16,100
12	37,302	0.9739	5,108	1,509	15,143	15,542	0.9735	5,100	1,364	15,231	15,607
13	26,806	0.9422	5,121	1,020	9,622	11,043	0.9421	5,118	947	9,652	11,089
14	25,509	0.9738	3,599	1,053	10,386	10,471	0.9734	3,595	961	10,438	10,515
15	24,467	0.9763	3,279	1,038	9,628	10,522	0.9758	3,283	972	9,649	10,563
16	28,960	0.9727	4,015	1,230	11,852	11,863	0.9722	4,021	1,128	11,893	11,918
17	28,835	0.9555	5,114	1,081	11,470	11,170	0.9548	5,113	1,004	11,513	11,205
18	21,962	0.9716	3,089	891	8,132	9,850	0.9713	3,083	815	8,189	9,875
19	26,186	0.9676	4,536	1,208	10,827	9,615	0.9666	4,528	1,111	10,896	9,651
20	19,959	0.9709	2,545	791	7,906	8,717	0.9707	2,549	711	7,954	8,745
21	11,342	0.9750	1,579	494	4,218	5,051	0.9745	1,578	454	4,239	5,071
22	12,968	0.9730	1,666	621	4,932	5,749	0.9727	1,663	558	4,960	5,787
**Total**	**784,256**		**114,324**	**32,831**	**312,443**	**324,658**		**114,161**	**30,161**	**314,037**	**325,897**

CHR – chromosome; GT rate – genotyping rate; --geno – genotype missingness <0.015; --maf – minor allele frequency MAF>5%; --hwe – Hardy-Weinberg exact test p>1*10^{-6}.

Supplementary Table S8 Bayesian mixed-model analysis BOLT-LMM quality control

**WOMEN**	**WHI_{UKB}**	**$WHR_{adj}BMI$**	**$ABSI_{UKB}$**	**$WC_{adj}BMI$**	**HI_{UKB}**	**$HC_{adj}BMI$**
**Heretability**						
Estimated (pseudo-)heritability: h^2_g	0.258	0.253	0.211	0.244	0.178	0.273
Approximate standard error: 316/N	0.001	0.001	0.001	0.001	0.001	0.001
**Variance parameters**						
sigma^2_K	0.251	0.245	0.203	0.238	0.177	0.267
logDelta	1.057	1.083	1.316	1.132	1.532	0.980
f	$4.6*10^{-5}$	$5.5*10^{-5}$	$-2.0*10^{-4}$	$6.3*10^{-5}$	$-6.1*10^{-5}$	$1.7*10^{-3}$
**Mixed model associations statistics** (inflation of LINREG chisq stats estimated using MLMe as reference)						
SNPs after outlier window removal [a]	319,769	319,769	321,169	321,114	321,788	321,016
Intercept of LD Score regression (ref stats)	1.230 (0.025)	1.225 (0.025)	1.183 (0.020)	1.213 (0.022)	1.156 (0.017)	1.202 (0.021)
Estimated attenuation	0.233 (0.050)	0.229 (0.053)	0.215 (0.055)	0.236 (0.029)	0.267 (0.029)	0.175 (0.027)
Intercept of LD Score regression (cur stats)	1.235 (0.024)	1.239 (0.024)	1.198 (0.021)	1.265 (0.022)	1.276 (0.016)	1.533 (0.024)
Calibration factor (ref/cur) (inf model) [b]	0.996 (0.003)	0.989 (0.003)	0.987 (0.002)	0.959 (0.003)	0.906 (0.003)	0.784 (0.006)
Linear regression intercept inflation [c]	1.0041	1.0113	1.0130	1.0425	1.1040	1.2757
Proportion variance explained (inf model) [b]	0.092	0.089	0.068	0.088	0.048	0.108
**Calibration stats: mean and lambdaGC** (over SNPs used in GRM)						
Mean linear regression [c] (324,658 good SNPs)	2.028	2.034	1.876	1.969	1.735	2.502
λ_{GC} linear regression model [c]	1.515	1.521	1.467	1.559	1.516	2.004
Mean BOLT-LMM infinitesimal model [b] (324,658 good SNPs)	2.102	2.095	1.899	1.952	1.621	2.219
λ_{GC} BOLT-LMM infinitesimal model [b]	1.522	1.512	1.468	1.519	1.374	1.596
**MEN**	**WHI_{UKB}**	**$WHR_{adj}BMI$**	**$ABSI_{UKB}$**	**$WC_{adj}BMI$**	**HI_{UKB}**	**$HC_{adj}BMI$**
**Heretability**						
Estimated (pseudo-)heritability: h^2_g	0.170	0.162	0.149	0.225	0.145	0.276
Approximate standard error: 316/N	0.002	0.002	0.002	0.002	0.002	0.002
**Variance parameters**						
sigma^2_K	0.162	0.155	0.138	0.217	0.144	0.271
logDelta	1.587	1.640	1.745	1.234	1.771	0.962
f	$-4.8*10^{-5}$	$1.7*10^{-3}$	$2.4*10^{-4}$	$9.3*10^{-5}$	$7.7*10^{-4}$	$1.4*10^{-3}$

Continues on next page

**MEN**	**WHI_{UKB}**	**$WHR_{adj}BMI$**	**$ABSI_{UKB}$**	**$WC_{adj}BMI$**	**HI_{UKB}**	**$HC_{adj}BMI$**
**Mixed model associations statistics** (inflation of LINREG chisq stats estimated using MLMe as reference)						
SNPs after outlier window removal [a]	323,858	323,858	323,858	323,858	323,858	323,123
Intercept of LD Score regression (ref stats)	1.104 (0.016)	1.099 (0.015)	1.106 (0.014)	1.151 (0.021)	1.117 (0.013)	1.168 (0.022)
Estimated attenuation	0.154 (0.049)	0.147 (0.053)	0.204 (0.046)	0.203 (0.036)	0.279 (0.033)	0.163 (0.043)
Intercept of LD Score regression (cur stats)	1.108 (0.015)	1.101 (0.015)	1.171 (0.013)	1.323 (0.021)	1.189 (0.015)	1.410 (0.025)
Calibration factor (ref/cur) (inf model) [b]	0.996 (0.002)	0.998 (0.002)	0.945 (0.003)	0.871 (0.004)	0.939 (0.003)	0.829 (0.007)
Linear regression [c] intercept inflation	1.00363	1.00187	1.05871	1.14861	1.06515	1.20666
Proportion variance explained (inf model) [b]	0.042	0.039	0.035	0.070	0.031	0.104
**Calibration stats: mean and lambdaGC** (over SNPs used in GRM)						
Mean linear regression [c] (325,897 good SNPs)	1.668	1.663	1.600	1.923	1.487	2.275
λ_{GC} linear regression model [c]	1.383	1.373	1.420	1.651	1.387	1.841
Mean BOLT-LMM infinitesimal model [b] (325,897 good SNPs)	1.679	1.675	1.520	1.749	1.420	2.081
λ_{GC} BOLT-LMM infinitesimal model [b]	1.390	1.380	1.344	1.448	1.312	1.551

$ABSI_{UKB}$ – a body shape index calibrated for UK Biobank participants, i.e. calculated using allomeric regression coefficients derived from UK Biobank data; **$HC_{adj}BMI$** – hip circumference adjusted for body mass index (BMI); **HI_{UKB}** – hip index calibrated for UK Biobank participants; **$WC_{adj}BMI$** – waist circumference adjusted for BMI; **WHI_{UKB}** – waist-to-hip index calibrated for UK Biobank participants; **$WHR_{adj}BMI$** – waist-to-hip ratio adjusted for BMI; λ_{GC} – genomic inflation factor based on the coreset of variants used in the genetic relationship matrix (GRM); [a] – variants (single nucleotide polymorphisms, SNPs) passing filters before outlier removal (322,638 out of 324,658 for women, 323,858 out of 325,897 for men); masking windows around outlier SNPs (chisq > 219.9 for women, chisq > 186.8 for men); [b] – BOLT-LMM using a Gaussian SNP effect prior, equivalent to the standard “infinitesimal” mixed model; [c] – linear regression using 20 principal component covariates.

Supplementary Table S9 Candidate SNPs identified with FUMA

(separate file *TABS9_snps.zip* for file *TABS9_snps.csv*)

Independent significant single nucleotide polymorphisms (SNPs) were variants with genome wide significance ($p \leq 5*10^{-8}$) and in approximate linkage equilibrium with each other ($r^2<0.6$ within 1 Mb distance). Candidate SNPs were variants with nominal significance $p \leq 0.05$, within 1 Mb distance and in linkage disequilibrium at $r^2 \geq 0.6$ with an independent significant SNP (based on FUMA v1.3.6a output per sex and index). Note that for all annotations gene symbol Mar-01 should be MARCH1.

Index: ABSIUKB – a body shape index calibrated for UK Biobank participants, i.e. calculated using allomeric regression coefficients derived from UK Biobank data; **HC** – hip circumference adjusted for body mass index (BMI); **HIUKB** – hip index calibrated for UK Biobank participants; **WC** – waist circumference adjusted for BMI; **WHI** – waist-to-hip index calibrated for UK Biobank participants; **WHR** – waist-to-hip ratio adjusted for BMI;

uniqID – unique identifier generated by FUMA with chromosome, position, and alleles in alphabetical order;

rsID – user generated unique identifier provided as input for FUMA, comprising chromosome, position, minor allele, major allele;

chr – chromosome number;

pos – position on the chromosome;

non_effect_allele – the major allele;

effect_allele – the minor allele, with frequency <0.5 determined in the dataset (note that for variants with frequency close to 0.5 the minor allele may differ between men and women);

MAF – minor allele frequency;

INFO – imputation quality score (INFO>0.9 for all candidate SNPs);

gwasP – p-value from BOLT-LMM infinitesimal model for the corresponding body-shape index;

beta / **se** – regression coefficient and standard error from BOLT-LMM infinitesimal model for the corresponding body-shape index;

r^2 – the maximum r^2 of the SNP with one of the independent significant SNP;

IndSigSNP – rsID of the independent significant SNP corresponding to the maximum r^2;

GenomicLocus – number of the corresponding genomic risk locus (further details in Supplementary Table S9);

nearestGene – nearest gene annotated with ANNOVAR implemented in FUMA;

dist – distance to the nearest gene from FUMA (SNPs located in the gene body or 1kb up- or down-stream of TSS or TES have 0);

func – functional consequence of the SNP on the gene obtained from ANNOVAR;

exonic_func – functional consequence of an exonic SNP on the gene obtained from ANNOVAR;

CADD – Combined Annotation Dependent Depletion (deleteriousness) score, computed based on 63 annotations;

RDB – RegulomeDB categorical score (from 1a to 7, with 1a being the highest score, indicating that the SNP has the most biological evidence to be regulatory element);

minChrState – The minimum 15-core chromatin state across 127 tissue/cell type;

commonChrState – the most common 15-core chromatin state across 127 tissue/cell types;

posMapFilt – whether the SNP was used for positional mapping or not. 1 is used, otherwise 0.

SNP – rs identifier (when unavailable, replaced with ID generated from Variance Effect Predictor (VEP) v.90);

gcSNP – rs identifier as for SNP, but when unavailable replaced with chromosome:position (used for matching with NHGRI-EBI GWAS Catalog;

NEAREST_GENES – nearest gene(s) annotated with VEP;

NEAREST_DIST – distance to nearest gene from VEP;

Damaging – damaging variant according to VEP;

CONSEQUENCES – biological consequences according to VEP.

Catalog – match with SNPs reported in the NHGRI-EBI GWAS Catalog (https://www.ebi.ac.uk/gwas/home, accessed on 07/04/2021) in association with the corresponding traditional body-shape index (with or without adjustment for body mass index, BMI), i.e. the waist-to-hip ratio for WHIUKB and WHR (catalogue sets EFO_0004343, EFO_0007788, EFO_0004302), waist circumference for ABSIUKB and WC (EFO_0004342, EFO_0007789, EFO_0004302), or hip circumference for HIUKB and HC (EFO_0005093, EFO_0008039, EFO_0004302) (1 – Yes, 0 – No);

WHR – match with SNPs reported in association with WHR or $WHR_{adj}BMI$ (1/0);

WC – match with SNPs reported for WC or $WC_{adj}BMI$ (1/0);

HC – match with SNPs reported for HC or $HC_{adj}BMI$ (1/0);

Height – match with SNPs reported for height (EFO_0004339, EFO_0004302) (1 – Yes, 0 – No);

Cancer – match with SNPs reported for cancer (EFO_0000331) (1 – Yes, 0 – No);

SexDiff – p-value for sex difference of effect size $p_{sex}<5*10^{-6}$;

IndexDiff – p-value for difference of effect size between the corresponding allometric and traditional index $p_{difference}<5*10^{-6}$.

Supplementary Table S10 Results from FUMA and MAGMA

(separate file *TABS10_FUMA_MAGMA.xlsx*)

<u>IndSigSNPs</u> – independent significant single nucleotide polymorphisms (SNPs) with genome wide significance ($p \leq 5*10^{-8}$) and in approximate linkage equilibrium with each other ($r^2<0.6$ within 1 Mb distance) (based on FUMA v.1.3.6a output per sex and index).

Index: ABSIUKB – a body shape index calibrated for UK Biobank participants, i.e. calculated using allomeric regression coefficients derived from UK Biobank data; **HC** – hip circumference adjusted for body mass index (BMI); **HIUKB** – hip index calibrated for UK Biobank participants; **WC** – waist circumference adjusted for BMI; **WHI** – waist-to-hip index calibrated for UK Biobank participants; **WHR** – waist-to-hip ratio adjusted for BMI;

No – number of the independent significant SNP per index (ordered by chromosome and position);

GenomicLocus – number of genomic risk locus to which the SNP was allocated (details for genomic loci are shown in tab GenomicRiskLoci;

uniqID – unique identifier generated by FUMA with chromosome, position, and alleles in alphabetical order;

rsID – user generated unique identifier provided as input for FUMA, comprising chromosome, position, minor allele, major allele;

chr – chromosome number;

pos – position on the chromosome;

p – p-value from BOLT-LMM infinitesimal model for the corresponding body-shape index;

nSNPs – number of candidate SNPs clumped with the independent significant SNP (with nominal significance $p \leq 0.05$, within 1 Mb distance and in linkage disequilibrium at $r^2 \geq 0.6$);

SNP – rs identifier (when unavailable, replaced with ID generated from Variance Effect Predictor (VEP) v.90);

gcSNP – rs identifier as for SNP, but when unavailable replaced with chromosome:position (used for matching with NHGRI-EBI GWAS Catalog;

nearestGene – nearest gene annotated with ANNOVAR implemented in FUMA;

dist – distance to the nearest gene from FUMA;

NEAREST_GENES – nearest gene(s) annotated with VEP;

NEAREST_DIST – distance to nearest gene from VEP;

Catalog – match of any candidate SNP in the LD block (clump) of the independent significant SNP (i.e. in high linkage equilibrium, $r^2 \geq 0.6$) with SNPs reported in the NHGRI-EBI GWAS Catalog (https://www.ebi.ac.uk/gwas/home, accessed on 07/04/2021) in association with the corresponding traditional body-shape index (with or without adjustment for body mass index, BMI), i.e. the waist-to-hip ratio for WHIUKB and WHR (catalogue sets EFO_0004343, EFO_0007788, EFO_0004302), waist circumference for ABSIUKB and WC (EFO_0004342, EFO_0007789, EFO_0004302), or hip circumference for HIUKB and HC (EFO_0005093, EFO_0008039, EFO_0004302) (1 – Yes, 0 – No);

CatalogDir – match with SNPs reported for the corresponding traditional body-shape index (2 – direct match of the independent significant SNP, 1 – match of a candidate SNP in the clump, 0 – no match for any candidate SNP in the clump);

WHR – match of any candidate SNP in the clump with SNPs reported in association with WHR or $WHR_{adj}BMI$ (1/0);

WC – match for any candidate SNP in the clump with SNPs reported for WC or $WC_{adj}BMI$ (1/0);

HC – match for any candidate SNP in the clump with SNPs reported for HC or $HC_{adj}BMI$ (1/0);

Height – match for any candidate SNP in the clump with SNPs reported for height (EFO_0004339, EFO_0004302) (1 – Yes, 0 – No);

HeightDir – match with SNPs reported for height (2 – direct match of the independent significant SNP, 1 – match of a candidate SNP in the clump, 0 – no match for any candidate SNP in the clump);

Cancer – match for any candidate SNP in the clump with SNPs reported for cancer (EFO_0000311) (1 – Yes, 0 – No);

CancerDir – match with SNPs reported for cancer (2 – direct match of the independent significant SNP, 1 – match of a candidate SNP in the clump, 0 – no match for any candidate SNP in the clump);

SexDiff – p-value for sex difference of effect size for the independent significant SNP $p_{sex}<5*10^{-6}$ (1 – Yes, 0 – No);

IndexDiff – p-value for difference of effect size between the corresponding allometric and traditional index for the independent significant SNP $p_{difference}<5*10^{-6}$ (1/0);

SexDiff_P – p-value for sex difference of effect size for the independent significant SNP;

IndexDiff_P – p-value for difference of effect size between the corresponding allometric and traditional index for the independent significant SNP;

ABSIHIDiff_P – p-value for difference of effect size between $ABSI_{UKB}$ and HI_{UKB} for the independent significant SNP.

leadSNPs – independent significant SNPs within 1 Mb distance of each other and with $r^2 \geq 0.1$ were further clumped and were represented by a lead SNP (the one with the lowest p-value) (based on FUMA output per sex and index). Column headings as per IndSigSNPs tab, with information corresponding to the lead SNP, except the following:

nIndSigSNPs – number of independent significant SNPs in the clump;

IndSigSNPs – rsID of the independent significant SNPs in the clump, separated with (;);

Catalog – match with SNPs reported in association with the corresponding traditional body-shape index for any candidate SNP in the clump;

CatalogDir – match with SNPs reported for the corresponding traditional body-shape index (2 – direct match of the lead SNP, 1 – match of a candidate SNP in the clump, 0 – no match);

Height – marked as 1 if any candidate SNP in the LD block of the lead SNP (i.e. in high linkage equilibrium, $r^2 \geq 0.6$) has been reported in association with height;

HeightDir – match with SNPs reported for height (2 – direct match of the lead SNP, 1 – match of any candidate SNP in the LD block of the lead SNP, 0 – no match for any candidate SNP in the LD block of the lead SNP);

Cancer – marked as 1 if any candidate SNP in the LD block of the lead SNP has been reported in association with cancer;

CancerDir – match with SNPs reported for cancer (2 – direct match of the lead SNP, 1 – match of any candidate SNP in the LD block of the lead SNP, 0 – no match for any candidate SNP in the LD block of the lead SNP).

GenomicRiskLoci – lead SNPs within 250 kb distance of each other were further clumped into non-overlapping independent significant genetic risk loci, represented by a locus lead SNP (the one with the lowest p-value) (based on FUMA output per sex and index). Column headings as per IndSigSNPs and leadSNPs tabs, with information corresponding to the locus lead SNP, except the following:

start – position of the earliest candidate SNP in the locus;

end – position of the earliest candidate SNP in the locus;

nLeadSNPs – number of lead SNPs in the clump;

LeadSNPs – rsID of the lead SNPs in the clump, separated with semi-colon (;);

Catalog – match with SNPs reported in the NHGRI-EBI GWAS Catalog in association with the corresponding traditional body-shape index for any candidate SNP in the locus clump;

CatalogDir – match with SNPs reported for the corresponding traditional body-shape index (2 – direct match of the locus lead SNP, 1 – match of a candidate SNP in the locus clump, 0 – no match);

Height – marked as 1 if any candidate SNP in the LD block of the locus lead SNP (i.e. in high linkage equilibrium, $r^2 \geq 0.6$) has been reported in association with height;

HeightDir – match with SNPs reported for height (2 – direct match of the locus lead SNP, 1 – match of any candidate SNP in the LD block of the locus lead SNP, 0 – no match for any candidate SNP in the LD block of the locus lead SNP);

Cancer – marked as 1 if any candidate SNP in the LD block of the locus lead SNP has been reported in association with cancer;

CancerDir – match with SNPs reported for cancer (2 – direct match of the locus lead SNP, 1 – match of any candidate SNP in the LD block of the locus lead SNP, 0 – no match for any candidate SNP in the LD block of the locus lead SNP).

magma.genes – all genes used in MAGMA v.1.08 implemented in FUMA v1.3.6a. Significance for the corresponding body-shape index and sex is determined by $p_{adj}<0.05$, after Bonferroni correction for 19,088 genes of the corresponding unadjusted p-value (P_INDEX_Sex). The output is based on MAGMA, which uses the complete distribution of variants with high imputation quality INFO>0.9 and minor allele frequency MAF>0.01.

N – gene number (1 to 19,088);

GENE – Ensembl v92 identification number;

CHR – chromosome number;

START/STOP – annotation boundaries of the gene, including a 1 kb window at each end;

NSPS – number of SNPs annotated to the gene found in the data;

NPARAM – number of parameters used in the model by MAGMA;

SYMBOL – Ensembl gene symbol;

ZSTAT – the Z-value for the gene, calculated by MAGMA based on its (permutation) p-value, used as the measure of gene association in the gene-level analyses;

P – the unadjusted p-value calculated by MAGMA for the gene, based on a SNP-wide mean model using user supplied p-values (for all SNPs annotated to the gene), obtained from BOLT-LMM infinitesimal models for the corresponding body-shape index (note that Bonferroni correction for 19,088 genes has to be applied to calculate the adjusted p-value (p_{adj}), which determines significance at a nominal level p<0.05);

Annotation of ZSTAT and P – column headings are annotated with index and sex as follows:
ABSIUKB – a body shape index calibrated for UK Biobank participants, i.e. calculated using

allomeric regression coefficients derived from UK Biobank data; **HC** – hip circumference adjusted for body mass index (BMI); **HIUKB** – hip index calibrated for UK Biobank participants; **WC** – waist circumference adjusted for BMI; **WHI** – waist-to-hip index calibrated for UK Biobank participants; **WHR** – waist-to-hip ratio adjusted for BMI; **0** – in women; **1** – in men;

WHR – match with gene symbols for variants reported in the NHGRI-EBI GWAS Catalog in association with WHR or $WHR_{adj}BMI$ (1 – Yes, 0 – No);

WC – match with gene symbols for variants reported in association with WC or $WC_{adj}BMI$ (1/0);

HC – match with gene symbols for variants reported in association with HC or $HC_{adj}BMI$ (1/0);

Fat – match with gene symbols for variants reported in association with body composition, fat distribution and visceral fat: catalogue sets EFO_0004341, EFO_0004765, EFO_0004767, EFO_0005106, EFO_0004302 (1/0);

BMI – match with gene symbols for variants reported in association with BMI, weight, subcutaneous adipose tissue and body fat: catalogue sets EFO_0004338, EFO_0004340, EFO_0004764, EFO_0004766, EFO_0005409, EFO_0007800, EFO_0004302 (1/0);

Height – match with gene symbols for variants reported in association with height: catalogue sets EFO_0004339 and EFO_0004302 (1/0);

Cancer – match with gene symbols for variants reported in association with cancer: catalogue set EFO_0000331 (1/0).

magma.regions – significant genes identified in MAGMA for the corresponding body-shape index with gene boundaries within 250 kb of each other were clumped in genomic risk regions represented by a lead gene with the lowest p-value. This output incudes all significant genes per body-shape index and sex, with columns corresponding to the magma.genes tab, except for:

MEDIAN – median between the annotation boundaries of the significant gene (START/STOP);

REGION – region number;

START_MIN – annotation boundary START of the gene with the earliest position in the region;

STOP_MAX – annotation boundary STOP of the gene with the latest position in the region;

P_MIN – p-value of the lead gene in the region;

REG_GENE – Ensembl identification number of the lead gene for the region;

REG_SYMBOL – symbol of the lead gene in the region;

REG_MEDIAN – median between the annotation boundaries of the region (START_MIN to STOP_MAX);

REG_COUNT – number of significant genes clumped in the region;

REG_CATALOG – match with gene symbols for variants reported in the NHGRI-EBI GWAS Catalog in association with the corresponding traditional body-shape index for any significant gene included in the region (1 – Yes, 0 – No);

REG_CATALOG_DIR – match with genes reported for the corresponding traditional body-shape index (2 – direct match of the lead gene, 1 – match of any significant gene included in the region, 0 – no match).

REG_HEIGHT_DIR – match with genes reported for height (1 – direct match of the lead gene, 0 – no match or match only of a clumped significant gene, not the lead gene).

REG_CANCER_DIR – match with genes reported for cancer (1 – direct match of the lead gene, 0 – no match or match only of a clumped significant gene, not the lead gene).

Columns Sex, Index, CHR, REGION, START_MIN, STOP_MAX, P_MIN and all columns marked with REG can be compacted to unique values for the regions per index and sex.

magma.gsa – MAGMA gene-set analysis output (includes all gene sets). Significance for the corresponding body-shape index and sex is determined by $p_{adj}<0.05$, after Bonferroni correction for 15,485 gene sets of the corresponding unadjusted p-value (P_INDEX_Sex).

FULL_NAME – name of the gene set in four categories: curated gene sets (including the name of the public database, e.g. KEGG, Biocarta, Reactome etc.) and the three gene ontology categories: biological pathway (GO-BP), cellular component (GO-CC) and molecular function (GO-MF);

NGENES – the number of genes in the data included in the set;

BETA – the regression coefficient for the gene-set calculated with MAGMA;

BETA_STD – the semi-standardized regression coefficient, corresponding to the predicted change in Z-value given a change of one standard deviation in the predictor gene set (i.e. BETA divided by the variable's standard deviation), calculated by MAGMA;

SE – the standard error of the regression coefficient;

P – the unadjusted p-value calculated by MAGMA for the gene set, based on competitive gene-set analysis (note that Bonferroni correction for 15,485 gene sets has to be applied to calculate the adjusted p-value (p_{adj}), which determines significance at a nominal level $p<0.05$).

Annotation of BETA, BETA_STD, SE and P – column headings are annotated with index and sex as in **magma.genes** tab;

magma.eqtl – MAGMA output for associations with expression Quantitative Trait Loci (eQTL) from the Genotype-Tissue Expression (GTEx) v8.0 database (includes all examined individual tissues). Significance for the corresponding body-shape index and sex is determined by $p_{adj}<0.05$, after Bonferroni correction for 54 tissues of the corresponding unadjusted p-value (P_INDEX_Sex). A total of 17,299 genes were used in the analysis for each tissue.

FULL_NAME – name of the individual tissue;

NGENES – the number of genes in the data included in the set;

BETA – the regression coefficient for the tissue calculated with MAGMA;

BETA_STD – the semi-standardized regression coefficient;

SE – the standard error of the regression coefficient;

P – the unadjusted p-value calculated by MAGMA for the tissue, based on competitive gene property analysis (note that Bonferroni correction for 54 tissues has to be applied to calculate the adjusted p-value (p_{adj}), which determines significance at a nominal level $p<0.05$).

Annotation of BETA, BETA_STD, SE and P – column headings are annotated with index and sex as in **magma.genes** tab;

anthropometry_traits – rules for assignment of specific anthropometric category for traits included in the general catalogue set EFO_0004302 anthropometric measurements.

N – number of the reported trait;

Trait.s – name of the reported trait in the NHGRI-EBI GWAS Catalog;

Freq – frequency of the reported trait in catalog set EFO_0004302;

ReSet – name of the assigned individual anthropometric category defined in the study.

efo_traits – details of the NHGRI-EBI GWAS Catalog sets used for matching in the study (https://www.ebi.ac.uk/gwas/home, accessed on 07/04/2021).

EFO – catalogue set number;

Trait – anthropometric trait defined in the study;

Name – name of the set in the NHGRI-EBI GWAS Catalog;

Assoc – number of variants included in the catalogue set;

Studies – number of studies included in the catalogue set;

Set – anthropometric category used for matching: 1 – waist-to-hip ratio; 2 – waist circumference; 3 – hip circumference; 4 – (Fat) body composition, fat distribution and visceral adipose tissue; 5 – BMI, weight, body fat, subcutaneous adipose tissue; 6 – height; 7 – the general anthropometric measurements catalogue set, which was distributed between the other anthropometric categories).

Note that for all annotations gene symbol Mar-01 should be MARCH1.

Supplementary Table S11 GWAS Summary statistics for all candidate SNPs

(separate file *TABS11_gwas.bolt.snps.zip*, including file *TABS11a_gwas.bolt.snps.f.csv* for women and file *TABS11b_gwas.bolt.snps.m.csv* for men)

Independent significant single nucleotide polymorphisms (SNPs) identified with FUMA v.1.3.6a were variants with genome wide significance ($p \leq 5*10^{-8}$) and in approximate linkage equilibrium with each other ($r^2<0.6$ within 1 Mb distance). Candidate SNPs were variants with nominal significance $p \leq 0.05$, within 1 Mb distance and in linkage disequilibrium at $r^2 \geq 0.6$ with an independent significant SNP. The output in these files is based on BOLT-LMM infinitesimal models. Note that for all annotations, gene symbol Mar-01 should be MARCH1.

rsID – identity number constructed from Chromosome, Position (base pairs), A1 allele and A2 allele;

SNP – single nucleotide polymorphism (rs number or when missing chromosome, position and alleles provided by Variant Effect Predictor (VEP), v90);

CHR – chromosome number;

BP – position on chromosome (base pairs);

A1 – minor allele (allele frequency determined in the dataset <0.5, note that for alleles with frequency near 0.5 the minor allele may differ between men and women);

A2 – major allele;

MAF – frequency of A1 allele in the examined population;

INFO – imputation quality factor (INFO>0.9 for all SNPs used in FUMA);

BETA – regression coefficient (corresponding to A1 allele) from BOLT-LMM infinitesimal model; **SE** – standard error from BOLT-LMM infinitesimal model;

CHISQ_BOLT_LMM_INF – χ^2 statistics from BOLT-LMM infinitesimal model;

P_BOLT_LMM_INF – p-value from BOLT-LMM infinitesimal model;

Annotation of BETA, SE, CHISQ and P: ABSIUKB – a body shape index calibrated for UK Biobank participants, i.e. calculated using allomeric regression coefficients derived from UK Biobank data; **HC** – hip circumference adjusted for body mass index (BMI); **HIUKB** – hip index calibrated for UK Biobank participants; **WC** – waist circumference adjusted for BMI; **WHIUKB** – waist-to-hip index calibrated for UK Biobank participants; **WHR** – waist-to-hip ratio adjusted for BMI.

Damaging / CONSEQUENCES – provided by VEP;

NEAREST_DIST – smallest distance in BP to the nearest gene;

NEAREST_GENES – location with the nearest protein-coding transcription start site within a window of 200 kb from the position of the examined variant (if more than one location satisfied this requirement, these were separated with a coma).
